# ImmUQBench: a benchmark on uncertainty quantification of protein immunogenicity prediction

**DOI:** 10.1093/oxfimm/iqag003

**Published:** 2026-03-03

**Authors:** Alif Bin Abdul Qayyum, Amir Hossein Rahmati, Xiaoning Qian, Byung-Jun Yoon

**Affiliations:** Department of Electrical and Computer Engineering, Texas A&M University, College Station, TX 77843, United States; Department of Electrical and Computer Engineering, Texas A&M University, College Station, TX 77843, United States; Department of Electrical and Computer Engineering, Texas A&M University, College Station, TX 77843, United States; Department of Computer Science and Engineering, Texas A&M University, College Station, TX 77843, United States; Computing and Data Sciences, Brookhaven National Laboratory, Upton, NY 11973, United States; Department of Electrical and Computer Engineering, Texas A&M University, College Station, TX 77843, United States; Computing and Data Sciences, Brookhaven National Laboratory, Upton, NY 11973, United States

**Keywords:** Uncertainty Quantification (UQ), Bayesian Neural Network (BNN), variational inference, Protein Language Model (PLM), multi-modal representation learning, immunogenicity prediction

## Abstract

Discovering antigen proteins, capable of eliciting desired immune responses, is of paramount importance in developing immunogenic therapeutics for combating various diseases, particularly autoimmune disorders, infectious diseases, as well as cancers. Despite recent advances in artificial intelligence (AI) and machine learning (ML), accurate and generalizable immunogenicity prediction remains challenging due to limited labeled data and model over-simplifications. Uncertainty quantification (UQ) approaches are commonly used to address the aforementioned challenges when applying AI/ML methods with limited training data, aiming to reduce the risk of catastrophic errors. This study aims to systematically evaluate the performance of UQ methods for antigen immunogenicity prediction and to establish a benchmark for assessing model reliability in data-scarce setting. We here present *ImmUQBench*, a comprehensive benchmark that compares several well-known UQ methods for antigen immunogenicity prediction tasks. The benchmark assesses models in terms of predictive accuracy, calibration, and robustness under both in and out of distribution settings, providing standardized evaluation framework. Our evaluation reveals that different UQ strategies exhibit varying capabilities in capturing predictive uncertainty and maintaining robustness. This work yields critical insights into the performance and reliability of various UQ methods when applied to immunogenicity data, helping to identify which methods offer the most trustworthy predictions. *ImmUQBench* provides a unified platform for assessing UQ approaches in immunogenicity prediction, facilitating the development of more trustworthy AI/ML models for therapeutic antigen design. By offering insights into the strengths and limitations of existing UQ methods, our work facilitates more effective and reliable immunogenic therapeutic discovery.

## Introduction

Immunogenicity refers to the ability of a pathogen to provoke host immune responses. Identifying which pathogen proteins are likely to trigger an immune response is an absolutely vital step when developing new protein-based immunogenic therapeutics, e.g. vaccines [1, 2]. This process, often called *immunogenicity prediction*, is a key task that helps scientists anticipate and manage potential problems before a therapeutic even reaches clinical trials. Deep learning models have significantly advanced this task by enabling scalable and accurate predictions [3, 4]. However, their effectiveness is often hindered by the scarcity of labeled data and a mismatch between the task complexity and model assumptions, leading to suboptimal performance and limited generalizability, particularly when designing for broad viral efficacy.

The advent of large language models (LLMs) marks a pivotal advancement in natural language processing (NLP), fundamentally reshaping its capabilities [5–7]. This progress has, in turn, facilitated the emergence of general-purpose computational tools within the field of biology. In particular, the adaptation of language modeling techniques to proteins has led to the emergence of powerful protein language models (PLMs), which have demonstrated strong performance on a variety of downstream tasks [8–10]. These models frequently surpass traditional approaches and offer improved generalization capabilities.

Despite their empirical success, both traditional ML prediction models and PLM integrated DL models designed for downstream tasks often exhibit overconfident predictions and are prone to generating hallucinated outputs [11, 12], raising concerns about their reliability and trustworthiness in sensitive applications, e.g. safety and efficacy related therapeutic design. To mitigate these limitations, the machine learning community has increasingly turned to uncertainty quantification (UQ) techniques. Broadly, UQ methods fall into two categories: Bayesian approaches [13–15], which provide a principled probabilistic framework but can be computationally intensive or impractical, and non-Bayesian approaches [16–18], which are often more tractable and performant but lack strong theoretical guarantees.

**Figure 1:**
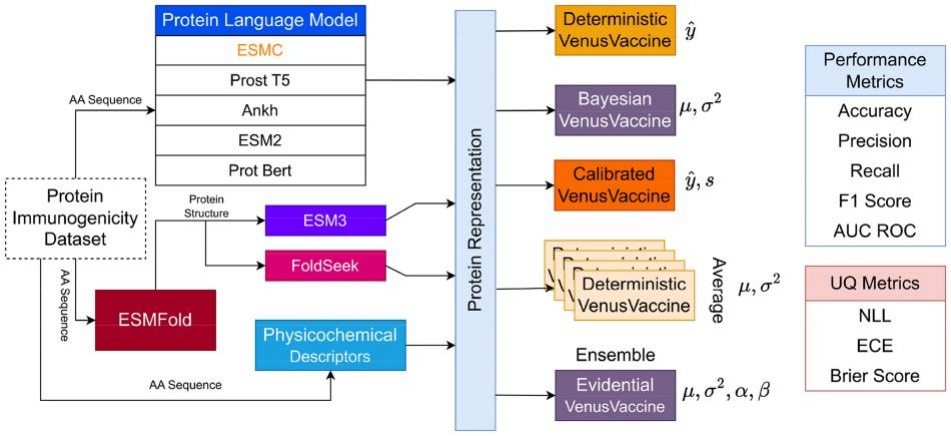
Benchmarking protein immunogenicity prediction. We adopt VenusVaccine [19] as the backbone architecture as an exemplar using both sequence and structure level representations, along with physico-chemical descriptors for immunogenicity prediction. We evaluate different uncertainty quantification strategies on the corresponding uncertainty-aware model variants according to both predictive and UQ metrics.

Despite the increasing importance of UQ in immunogenicity prediction, there is no comprehensive study comparing different UQ techniques for varied downstream applications. In this work, we fill this void by introducing **ImmUQBench**, a benchmark designed to systematically evaluate a diverse set of UQ methods in the context of immunogenicity prediction. We compare both Bayesian and non-Bayesian methods across various experimental scenarios, including in-distribution and out-of-distribution settings, to assess their predictive accuracy, uncertainty estimation quality, and robustness to distributional shifts. Additionally, we examine the impact of different protein sequence encoding schemes, highlighting robustness to alternate encodings that express the same underlying sequence information. To ensure a concise and focused presentation, all results in the main manuscript, unless otherwise specified are obtained using ESM-Cambrian [9] as the PLM. We adopt ESM-Cambrian because it demonstrates an overall superior empirical predictive performance and represents the most recent generation among the PLMs considered. Results based on the other PLMs are provided in the supplementary material. Figure 1 provides a detailed illustration of the **ImmUQBench** framework. Throughout all our experiments, although there is not a clear winner (as expected) among UQ techniques, almost all methods outperformed their deterministic alternative across nearly all metrics.

To the best of our knowledge, **ImmUQBench** is the first comprehensive benchmark to assess UQ methods for immunogenicity prediction on three distinct immunogenic protein data sources, an essential step in therapeutic design, including vaccine development. We briefly summarize our contributions below:

- *Pioneering Benchmark in Immunogenicity*: We introduce **ImmUQBench**, a benchmark for uncertainty quantification in immunogenicity prediction.
- *Extensive Evaluation of UQ across Several Data Distributions*: We systematically evaluate a wide range of Bayesian and non-Bayesian UQ approaches on three distinct immunogenic data sources, both across in-distribution and out-of-distribution scenarios.
- *Evaluation of Various Data Representation and Model Ablation*: We provide insights through extensive experiments and ablation studies to support antigen design and broadly effective therapeutic development.

The remainder of this paper is organized as follows: Section *UQ for Deep Neural Networks* reviews existing UQ approaches for deep neural network models; Section *ImmUQBench* presents the setup of ImmUQBench; Section *Experiments* describes the experimental settings and results; Section *Related Works* reviews related works; and Section *Conclusion & Limitations* concludes the study.

## UQ for deep neural networks

As Deep Learning models have become widely adopted across a broad range of tasks, their trustworthiness and reliability of their prediction have become essential and critically important, as they struggle to distinguish between in and out-of-distribution datasets [20, 21] as well as being sensitive to domain shift [22]. This is particularly important in safety critical tasks where data is often scarce and wrong predictions can lead to severe consequences. Hence, it is essential that these models express their uncertainty when confronting out-of-distribution data. Different approaches have been developed and utilized for uncertainty quantification that can be broadly categorized into Bayesian methods [13–15, 23–28] and non-Bayesian methods [16–18, 29–32].

In this work, for each category of UQ methods, we consider widely used and representative approaches.

### Bayesian methods

We begin with Monte Carlo (MC)-Dropout [15], which interprets dropout as a form of approximate Bayesian inference in deep Gaussian processes. This interpretation allows dropout to capture epistemic uncertainty by maintaining stochasticity at test time. Specifically, model predictions are obtained by performing multiple stochastic forward passes with dropout enabled and computing the predictive mean and variance from these passes. Formally, the predictive mean is approximated by averaging outputs over *T* stochastic passes, while the variance is classically estimated as the sample variance augmented by a model precision term $\tau^{-1}$, where τ corresponds to the assumed observation noise precision that can be identified by dropout rate, dataset size, and weight decay. In this work, we omit the $\tau^{-1}$ term and use the empirical variance of MC dropout predictions as a measure of model uncertainty. This enables tractable Bayesian approximation without incurring additional test-time complexity.

Next, we consider variational Bayesian last layer (VBLL) [33], which provides an efficient sampling-free approach to Bayesian modeling by maintaining a posterior only over the final layer of a neural network. By casting the training objective as a deterministic variational bound, VBLL introduces minimal overhead and is easily integrated into existing architectures, yielding principled uncertainty estimates without requiring stochastic forward passes.

We also include stochastic variational deep kernel learning (SVDKL) [34], which synergizes the representation power of deep neural networks with the non-parametric flexibility of Gaussian processes. SVDKL extends deep kernel learning (DKL) to classification and multi-task learning using a scalable variational inference framework. This allows for training on large-scale datasets via stochastic gradients, and supports more expressive covariance structures compared to prior DKL models.

SWAG [35] (stochastic weight averaging-Gaussian) is another Bayesian approximation technique that constructs a Gaussian posterior over network weights by leveraging the trajectory of stochastic gradient descent (SGD). It estimates the posterior mean via the running average of SGD iterates and approximates the covariance using both a low-rank approximation based on recent deviations from the mean and a diagonal component derived from the second moment. This results in a scalable approach to uncertainty estimation that enables Bayesian model averaging through weight sampling at test time.

Stochastic gradient Langevin dynamics (SGLD) [36] is another scalable Bayesian learning algorithm designed for large datasets, combining stochastic optimization with principles from Langevin dynamics. At each iteration, it performs a gradient-based update similar to stochastic gradient descent (SGD) but injects carefully calibrated Gaussian noise into the parameter updates. Under appropriate conditions on the step size and noise variance, the sequence of parameter samples produced by SGLD asymptotically follows the true Bayesian posterior, allowing for uncertainty quantification without the heavy computational cost of traditional Markov chain Monte Carlo methods.

Finally, we use the Laplace approximation (LA) [26], which approximates the posterior distribution over model weights with a Gaussian centered at the maximum a posteriori (MAP) estimate. This is achieved by performing a second-order Taylor expansion of the log-posterior, resulting in a Gaussian with covariance given by the inverse Hessian of the log-posterior evaluated at the MAP point. Formally, the posterior is approximated as $p(w|D)\approx N(\hat{w},H^{-1})$, where H is the Hessian of the negative log posterior and $\hat{w}$ is the MAP. This method provides a fast and principled estimate of uncertainty without requiring sampling during inference.

### Non-Bayesian methods

Among non-Bayesian approaches, we consider Deep Ensembles [30], which train an ensemble of *M* neural networks with different initializations. Each model outputs a probabilistic prediction, and the ensemble prediction is computed by averaging these outputs. This ensemble captures both model and data uncertainty and has been shown to outperform many Bayesian approximations in terms of calibration and robustness. Each network is typically trained using proper scoring rules such as negative log-likelihood to ensure meaningful probabilistic outputs.

We also evaluate evidential deep learning (EDL) [37], which explicitly models predictive uncertainty by placing a Dirichlet distribution over class probabilities. Rather than producing point estimates via softmax, the network outputs non-negative “evidence” values that parameterize the Dirichlet. This allows the model to represent both aleatoric and epistemic uncertainty in a unified framework. The loss function combines the Bayes risk (under an L2 norm) with a KL-divergence term that regularizes the model to prevent overconfident predictions, enabling uncertainty-aware classification from a single forward pass.

Finally, as all the aforementioned methods ultimately aim to produce well-calibrated and reliable predictive distributions, we also include Temperature Scaling (TS) [20] as a baseline for comparison. Temperature scaling is a post hoc calibration technique that adjusts the confidence of a classifier by optimizing a single non-negative scalar parameter T>0 on a validation set, typically using negative log-likelihood as the objective. By dividing the logits by *T* before applying the softmax, this method effectively increases the entropy of the predictive distribution, leading to better-calibrated output probabilities without altering the model’s accuracy.

## ImmUQBench

In this work, we focus on investigating UQ approaches in identifying whether proteins—originating from humans, bacteria, or viruses—are immunogenic. This task can be cast as a binary classification problem, where the model is trained to predict whether a given protein (or peptide segment) is an immunogenic antigen: an immunogenic antigen is an antigen that is capable of eliciting immune response when exposed to human immune system.

### Immunogenicity

Immunogenicity is linked to the therapeutic use of proteins and can result in serious clinical outcomes, including reduced treatment effectiveness or potentially life-threatening complications. Naturally, determining the cause of immunogenicity in biologic therapies is a necessary pursuit [38]. Particularly, immunogenicity prediction has become a central component in reverse vaccinology aiming to identify antigens that are capable of eliciting immune responses resulting in the formation of memory cells within the host organism [2, 19].

Researchers are increasingly focused on fast and precise prediction of immunogenic antigens for vaccine development, as this approach minimizes costs and associated risks, while supporting safe and effective responses to infectious disease threats. [39] use a simple linear scoring function to calculate immunogenicity score. DeepImmuno [3] introduces two deep learning models aimed at modeling T-cell immunity, which is crucial for the development of cancer immunotherapies and vaccines. Specifically, DeepImmuno-CNN predicts immunogenicity, while DeepImmuno-GAN generates immunogenic peptides. TRAP [40] presents a robust deep learning framework for predicting CD8^+^ T-cell epitopes from both pathogenic and self-peptides. It also estimates the immunogenic potential of MHC-I peptides by providing a prediction score along with a confidence measure. Some current methods also consider using physiochemical properties of amino acids for immunogenicity prediction.

As our core objective is to integrate and evaluate different UQ approaches for immunogenicity prediction, we adopt VenusVaccine [19], a cutting-edge multi-modal deep learning framework. Leveraging a dual-attention mechanism, VenusVaccine integrates sequence, structural, and physicochemical information to effectively interpret immunogenicity.

### Protein language models (PLMs)

The adaptation of LLMs—the advent of which marked a major shift in natural language processing (NLP)—to protein sequences has resulted in the emergence of advanced protein language models (PLMs) [10, 41, 42]. This adaptation—hence modeling of protein sequences—was enabled by equating words with amino acids and interpreting the entire protein sequences as sentences [43, 44]. Via self-supervised learning, generic PLMs are often pre-trained on large datasets of amino acid sequences, which then due to learning contextual residue representations [41, 42], they can serve as feature extractors for a wide-range of protein tasks, such as prediction of structure, binding residues, sub-cellular localization, and fold classification.

### Problem setup

Here, we formalize the problem, incorporating multi-modal information from protein sequences, structures, and physicochemical properties. Following [19], sequence and structure embeddings are extracted from pre-trained protein language models (PLMs). These embeddings are passed through the dual-attention module of VenusVaccine, which summarizes them into a unified representation:


$$H=\text{DualAtt}(E_{\text{seq}},E_{\text{strc}}),$$

where


$$E_{\text{seq}}={\text{PLM}}_{\text{seq}}(x)\in R^{L\times d},\;E_{\text{strc}}={\text{PLM}}_{\text{strc}}(x)\in R^{L\times d}$$

denote the sequence and structure embeddings of the amino acid sequence x of length *L*, respectively, and *d* is the embedding dimension. The attention output *H*, along with the sequence and physicochemical features, is concatenated and passed to a classifier:


$$\hat{y}=f_{\theta }(Z),\;Z=\text{concat}(E_{\text{seq}},E_{\text{pc}},H),\;\hat{y}\in \{0,1\},$$

where $f_{\theta }$ is a deterministic classifier parameterized by θ.

Bayesian Methods: In this work, for evaluating Bayesian methods, we treat θ as a random variable to enable uncertainty estimation and to evaluate the performance of different uncertainty quantification methods. Thus, the predictive distribution is given by:


$$\hat{y}=E_{\theta ∼p_{\Theta }(\theta )}[f_{\theta }(Z)].$$

In ImmUQBench, we have implemented MC-Dropout [15], SWAG [35], DVBLL [33], SVDKL [34], and LA [26].

As non-Bayesian methods employ varied and often method-specific mechanisms for uncertainty estimation, a general predictive formulation analogous to the Bayesian case is not readily available. Hence, we briefly outline the evaluation formulation for Deep Ensembles and EDL. In addition, we describe TS which is a widely-used calibration technique for adjusting predicted probabilities.

Deep Ensemble: By training *M* neural networks independently, we estimate the uncertainty. Specifically, each model outputs a prediction, and the ensemble predictive distribution is computed as the average,


$$\hat{y}=\frac{1}{M}\sum_{m=1}^{M}f_{\theta^{m}}(Z).$$

The diversity among the members captures the uncertainty. Instead of training the same model with different initialization or data shuffling, we employed different data representation for different models in the ensemble. Each ensemble consists of five models, each with similar architecture following VenusVaccine [19]. However, the amino acid sequence level encoding, $E_{\text{seq}}$, for different model in the ensemble comes from different PLMs. The 5 PLMs used in this work are: ESM-Cambrian [9], ProstT5 [45], Ankh [46], ESM-2 [47], and Prot-Bert [48].

EDL: In a binary classification, EDL models the class probability as a Beta distribution, $\text{Beta}(\alpha_{1},\alpha_{2})$. Considering the network outputs the evidence parameters, $\alpha_{i}=e_{i}+1,i\in \{1,2\}$, where $e_{i}>0$, the predictive probability for class *i* is given by,


$$\hat{y}=E[p]=\frac{\alpha_{i}}{\alpha_{1}+\alpha_{2}}.$$

Uncertainty is then captured through the variance of the Beta distribution.

TS: To improve the calibration of predicted probabilities, TS introduces a scalar temperature parameter T>0, which is optimized on a validation set by minimizing the negative log-likelihood. This adjustment rescales the logit outputs to produce softer probability distributions with T>1. That is, larger values of *T* spread probability mass more evenly across classes, increasing the entropy. Specifically, given the logit vector z, the calibrated probabilities are computed as:


$$q=\sigma \left(\frac{z}{T}\right),$$

where σ denotes the softmax function. TS adjusts confidence levels without affecting the model’s accuracy, making it a simple yet effective post hoc calibration method.

## Experiments

### Uncertainty evaluation metrics

We assess uncertainty quantification using three established metrics: Expected Calibration Error (ECE), negative log-likelihood (NLL), and the Brier score, which have been commonly employed in the literature. ECE and Brier scores are considered as calibration metrics while NLL is mostly regarded as an indicator of overconfidence.

A calibrated model is the one whose predicted probabilities match the empirical frequency of the output [49]. A well-calibrated model can prevent wrong decisions in case of high uncertainty. ECE is used to assess calibration. Particularly by partitioning predictions into *M* equally-spaced bins based on their prediction confidence, ECE can be calculated as [20, 49],


$$\mathrm{ECE}=\sum_{m=1}^{M}\frac{B_{m}}{N}|\text{acc}(B_{m})-\text{conf}(B_{m})|,$$

with *N* indicating the size of the dataset, and $\text{acc}(B_{m})=1/|B_{m}|$  $\sum_{i\in B_{m}}I({\hat{y}}_{i}=y_{i})$ and $\text{conf}(B_{m})=1/|B_{m}|\sum_{i\in B_{m}}P({\hat{y}}_{i})$ the average accuracy and confidence in bin $B_{m}$ with size $\left|B_{m}\right|$ respectively.

Calibration can also be evaluated by the Brier score, which is a proper scoring rule and a widely accepted tool in the context of uncertainty quantification due to its ability in assessing the quality of probabilistic predictions [49, 50]. Especially, it captures how correct a model is and if it expresses proper confidence levels, by measuring mean squared difference between predicted probabilities and predictions. For a binary class, it is,


$$\mathrm{Brier}=\frac{1}{N}\sum_{i=1}^{N}(y_{i}-P({\hat{y}}_{i}){)}^{2}$$

On the other hand, NLL is often used to detect overconfidence. It is computed as the negative log-probability assigned to the true label,


$$\mathrm{NLL}=\sum_{i=1}^{N}-\text{log}\;P({\hat{y}}_{i}=y_{i})$$

When a model is overconfident in an incorrect prediction, it assigns a high probability to the wrong class. Therefore, the log loss becomes very large, that results in a high NLL.

### Dataset

In this study, we use ImmunoDB, an immunogenicity database comprising 7216 labeled antigens derived from three distinct sources: bacteria, viruses, and humans [19]. Each antigen is labeled as either immunogenic (positive) or non-immunogenic (negative). The dataset is constructed through a combination of literature curation, database mining, and bioinformatics filtering, with most positive samples originating from previously published studies. To ensure quality, redundant sequences and samples from tail regions were filtered out. This process resulted in three curated subsets: Immuno-Virus, Immuno-Bacteria, and Immuno-Tumor. Owing to its rigorous quality control, diverse species coverage, and comprehensive sourcing, ImmunoDB provides a valuable benchmark for evaluating the robustness and generalizability of immunogenicity prediction models. To the best of our knowledge, at the time this study was conducted, it represents the most extensive labeled antigen resource available for this task. For the sake of simplicity, we will interchangeably use Immuno-Virus and Virus for the rest of this paper (similar policy for Bacteria and Tumor).

### Backbone architecture

In this work, we use VenusVaccine [19], a supervised deep learning model for immunogenicity prediction, as the backbone in our experiments. The model integrates sequence, structural, and physicochemical information using a dual attention mechanism. It encodes protein sequences with pretrained PLMs and represents structures at both atomic and peptide levels using FoldSeek [51] and ESM-3 [52], respectively. Handcrafted physicochemical descriptors are also included to enhance biological relevance.

The model employs a hierarchical cross-attention framework that fuses sequence and structure representations at multiple scales, enabling rich interaction across modalities. Attention pooling then compresses amino acid-level features into a protein-level vector by highlighting key regions, which is used for final binary classification of immunogenicity.

### Experimental settings

Unless otherwise specified, all reported results are performance statistics over five independent runs. For training the models, we followed the similar techniques and hyperparameters adopted in VenusVaccine [19]. For DVBLL, LA, and SVDKL, we modified the last MLP segment of the original VenusVaccine architecture by adding an extra linear layer and converted this extra linear layer as the probabilistic segment. This was aimed at promoting stable training while maintaining reasonable computational costs. The additional linear layer has dimension 64.

For BNNs, we obtained 64 MC sample predictions. All reported results in this work, except Tables 1–3, utilize protein sequence embeddings derived from the ESM-Cambrian protein language model [9]. It should be emphasized that the ensemble model also uses sequence embeddings extracted from all five different PLMs including ESM-Cambrian.

**Table 1:** ECE of different models with sequence level features from different PLMs.

Dataset	Model	ESMC	ProstT5	Ankh	ESM2	Prot Bert
Virus	Deterministic	0.0344${}_{\pm 0.0085}$	0.0457${}_{\pm 0.0170}$	0.0442${}_{\pm 0.0241}$	0.0313${}_{\pm 0.0084}$	0.0513${}_{\pm 0.0027}$
	TS	0.0354${}_{\pm 0.0087}$	0.0420${}_{\pm 0.0123}$	0.0390${}_{\pm 0.0202}$	**0.0293** ${}_{\pm 0.0077}$	0.0497${}_{\pm 0.0049}$
	LA	**0.0301** ${}_{\pm 0.0071}$	0.0382${}_{\pm 0.0103}$	**0.0370** ${}_{\pm 0.0106}$	0.0578${}_{\pm 0.0175}$	0.0451${}_{\pm 0.0148}$
	DVBLL	0.0514${}_{\pm 0.0151}$	0.0550${}_{\pm 0.0118}$	0.0450${}_{\pm 0.0282}$	0.0656${}_{\pm 0.0412}$	**0.0392** ${}_{\pm 0.0134}$
	EDL	0.1385${}_{\pm 0.0606}$	0.0657${}_{\pm 0.0180}$	0.1063${}_{\pm 0.0077}$	0.1539${}_{\pm 0.0424}$	0.1031${}_{\pm 0.0189}$
	SWAG	0.1313${}_{\pm 0.0227}$	0.0993${}_{\pm 0.0206}$	0.1577${}_{\pm 0.0280}$	0.1439${}_{\pm 0.0146}$	0.1157${}_{\pm 0.0326}$
	MCD	0.0344${}_{\pm 0.0094}$	0.0454${}_{\pm 0.0171}$	0.0449${}_{\pm 0.0219}$	0.0316${}_{\pm 0.0091}$	0.0492${}_{\pm 0.0030}$
	DKL	0.0398${}_{\pm 0.0116}$	0.0820${}_{\pm 0.0492}$	0.0839${}_{\pm 0.0851}$	0.0445${}_{\pm 0.0285}$	0.0926${}_{\pm 0.0655}$
	SGLD	0.0341${}_{\pm 0.0062}$	**0.0362** ${}_{\pm 0.0054}$	0.0442${}_{\pm 0.0128}$	0.0559${}_{\pm 0.0084}$	0.0487${}_{\pm 0.0042}$
Bacteria	Deterministic	0.0957${}_{\pm 0.0358}$	0.1101${}_{\pm 0.0338}$	0.1102${}_{\pm 0.0109}$	0.0696${}_{\pm 0.0228}$	0.0876${}_{\pm 0.0342}$
	TS	0.0862${}_{\pm 0.0385}$	0.0966${}_{\pm 0.0325}$	0.0954${}_{\pm 0.0127}$	0.0569${}_{\pm 0.0224}$	0.0806${}_{\pm 0.0296}$
	LA	0.1049${}_{\pm 0.0251}$	0.0954${}_{\pm 0.0312}$	0.0976${}_{\pm 0.0337}$	0.0994${}_{\pm 0.0382}$	0.1002${}_{\pm 0.0119}$
	DVBLL	0.1105${}_{\pm 0.0483}$	0.1033${}_{\pm 0.0553}$	0.0694${}_{\pm 0.0334}$	0.0963${}_{\pm 0.0219}$	0.0764${}_{\pm 0.0159}$
	EDL	**0.0655** ${}_{\pm 0.0140}$	0.0977${}_{\pm 0.0732}$	0.1202${}_{\pm 0.0361}$	0.1083${}_{\pm 0.0341}$	0.1016${}_{\pm 0.0596}$
	SWAG	0.1083${}_{\pm 0.0325}$	0.0966${}_{\pm 0.0393}$	0.1250${}_{\pm 0.0460}$	0.0886${}_{\pm 0.0280}$	0.1127${}_{\pm 0.0350}$
	MCD	0.0956${}_{\pm 0.0359}$	0.1086${}_{\pm 0.0326}$	0.1085${}_{\pm 0.0113}$	0.0668${}_{\pm 0.0222}$	0.0860${}_{\pm 0.0330}$
	DKL	0.0862${}_{\pm 0.0187}$	0.0553${}_{\pm 0.0102}$	0.1098${}_{\pm 0.0136}$	0.1139${}_{\pm 0.0505}$	0.0723${}_{\pm 0.0225}$
	SGLD	0.1256${}_{\pm 0.0179}$	**0.0443** ${}_{\pm 0.0091}$	**0.0433** ${}_{\pm 0.0085}$	**0.0383** ${}_{\pm 0.0024}$	**0.0357** ${}_{\pm 0.0062}$
Tumor	Deterministic	0.0753${}_{\pm 0.0263}$	0.1796${}_{\pm 0.0642}$	0.0994${}_{\pm 0.0444}$	0.1217${}_{\pm 0.0110}$	0.1532${}_{\pm 0.0787}$
	TS	0.0781${}_{\pm 0.0124}$	0.1644${}_{\pm 0.0499}$	0.1000${}_{\pm 0.0371}$	0.0952${}_{\pm 0.0163}$	0.1421${}_{\pm 0.0718}$
	LA	0.0979${}_{\pm 0.0213}$	0.1689${}_{\pm 0.0602}$	0.1805${}_{\pm 0.0228}$	0.1233${}_{\pm 0.0430}$	0.1131${}_{\pm 0.0375}$
	DVBLL	0.1333${}_{\pm 0.0406}$	0.2191${}_{\pm 0.0299}$	0.1314${}_{\pm 0.0496}$	0.1297${}_{\pm 0.0470}$	0.1420${}_{\pm 0.0277}$
	EDL	0.1219${}_{\pm 0.0125}$	**0.0717** ${}_{\pm 0.0121}$	0.1164${}_{\pm 0.0184}$	0.0832${}_{\pm 0.0151}$	**0.0642** ${}_{\pm 0.0177}$
	SWAG	0.1275${}_{\pm 0.0085}$	0.1196${}_{\pm 0.0113}$	0.1265${}_{\pm 0.0276}$	0.1252${}_{\pm 0.0553}$	0.1191${}_{\pm 0.0194}$
	MCD	**0.0747** ${}_{\pm 0.0241}$	0.1792${}_{\pm 0.0611}$	0.1033${}_{\pm 0.0404}$	0.1140${}_{\pm 0.0153}$	0.1541${}_{\pm 0.0799}$
	DKL	0.1135${}_{\pm 0.0240}$	0.1611${}_{\pm 0.0487}$	0.1194${}_{\pm 0.0755}$	0.1516${}_{\pm 0.0636}$	0.1341${}_{\pm 0.0840}$
	SGLD	0.2037${}_{\pm 0.0282}$	0.0848${}_{\pm 0.0124}$	**0.0628** ${}_{\pm 0.0157}$	**0.0782** ${}_{\pm 0.0156}$	0.0871${}_{\pm 0.0155}$

**Table 2:** NLL Of different models with sequence level features from different PLMs.

Dataset	Model	ESMC	ProstT5	Ankh	ESM2	Prot Bert
Virus	Deterministic	0.2559${}_{\pm 0.0225}$	0.3066${}_{\pm 0.0329}$	0.3046${}_{\pm 0.0557}$	0.2616${}_{\pm 0.0135}$	0.2886${}_{\pm 0.0125}$
	TS	0.2525${}_{\pm 0.0209}$	0.2923${}_{\pm 0.0197}$	0.2889${}_{\pm 0.0383}$	**0.2599** ${}_{\pm 0.0130}$	0.2819${}_{\pm 0.0096}$
	LA	**0.2486** ${}_{\pm 0.0079}$	0.2877${}_{\pm 0.0476}$	0.2790${}_{\pm 0.0217}$	0.2951${}_{\pm 0.0276}$	0.2909${}_{\pm 0.0233}$
	DVBLL	0.2915${}_{\pm 0.0460}$	0.3207${}_{\pm 0.0375}$	0.4686${}_{\pm 0.1857}$	0.3474${}_{\pm 0.1118}$	0.2873${}_{\pm 0.0203}$
	EDL	0.3825${}_{\pm 0.1314}$	0.2771${}_{\pm 0.0127}$	0.3024${}_{\pm 0.0112}$	0.3794${}_{\pm 0.0571}$	0.3043${}_{\pm 0.0222}$
	SWAG	0.3189${}_{\pm 0.0260}$	0.3054${}_{\pm 0.0288}$	0.3600${}_{\pm 0.0432}$	0.3304${}_{\pm 0.0167}$	0.3129${}_{\pm 0.0312}$
	MCD	0.2549${}_{\pm 0.0224}$	0.3017${}_{\pm 0.0291}$	0.2995${}_{\pm 0.0495}$	0.2612${}_{\pm 0.0131}$	0.2868${}_{\pm 0.0113}$
	DKL	0.3039${}_{\pm 0.0361}$	0.3984${}_{\pm 0.0862}$	0.3670${}_{\pm 0.1566}$	0.2937${}_{\pm 0.0698}$	0.3644${}_{\pm 0.1261}$
	SGLD	0.2855${}_{\pm 0.0105}$	**0.2737** ${}_{\pm 0.0099}$	**0.2517** ${}_{\pm 0.0056}$	0.2829${}_{\pm 0.0046}$	**0.2750** ${}_{\pm 0.0028}$
Bacteria	Deterministic	0.4961${}_{\pm 0.0485}$	0.6113${}_{\pm 0.1188}$	0.5823${}_{\pm 0.0598}$	0.4501${}_{\pm 0.0352}$	0.5059${}_{\pm 0.0734}$
	TS	0.4784${}_{\pm 0.0382}$	0.5545${}_{\pm 0.0785}$	0.5268${}_{\pm 0.0371}$	0.4375${}_{\pm 0.0250}$	0.4807${}_{\pm 0.0442}$
	LA	0.5780${}_{\pm 0.1031}$	0.5275${}_{\pm 0.0489}$	0.5599${}_{\pm 0.1258}$	0.5091${}_{\pm 0.0832}$	0.5550${}_{\pm 0.0812}$
	DVBLL	0.5663${}_{\pm 0.1132}$	0.6550${}_{\pm 0.1951}$	0.6513${}_{\pm 0.1136}$	0.5986${}_{\pm 0.0949}$	0.5104${}_{\pm 0.0439}$
	EDL	**0.4380** ${}_{\pm 0.0145}$	0.4917${}_{\pm 0.0786}$	0.5189${}_{\pm 0.0452}$	0.5436${}_{\pm 0.1234}$	0.4599${}_{\pm 0.0673}$
	SWAG	0.4950${}_{\pm 0.0613}$	0.5153${}_{\pm 0.0792}$	0.4980${}_{\pm 0.0402}$	0.4705${}_{\pm 0.0617}$	0.4697${}_{\pm 0.0254}$
	MCD	0.4932${}_{\pm 0.0461}$	0.5968${}_{\pm 0.1070}$	0.5725${}_{\pm 0.0605}$	0.4484${}_{\pm 0.0324}$	0.5000${}_{\pm 0.0661}$
	DKL	0.5011${}_{\pm 0.0296}$	0.5074${}_{\pm 0.0235}$	0.5370${}_{\pm 0.0317}$	0.5357${}_{\pm 0.0542}$	0.4797${}_{\pm 0.0234}$
	SGLD	0.6303${}_{\pm 0.0585}$	**0.4608** ${}_{\pm 0.0036}$	**0.4452** ${}_{\pm 0.0020}$	**0.4329** ${}_{\pm 0.0016}$	**0.4213** ${}_{\pm 0.0030}$
Tumor	Deterministic	0.4930${}_{\pm 0.0225}$	1.0011${}_{\pm 0.3935}$	0.5210${}_{\pm 0.0778}$	0.5398${}_{\pm 0.0154}$	0.6798${}_{\pm 0.2349}$
	TS	0.4919${}_{\pm 0.0217}$	0.8615${}_{\pm 0.2806}$	0.5042${}_{\pm 0.0612}$	**0.5196** ${}_{\pm 0.0157}$	0.6256${}_{\pm 0.1756}$
	LA	**0.4886** ${}_{\pm 0.0374}$	0.7930${}_{\pm 0.2498}$	0.7586${}_{\pm 0.0333}$	0.6226${}_{\pm 0.1342}$	0.5894${}_{\pm 0.0641}$
	DVBLL	0.5827${}_{\pm 0.1026}$	0.9336${}_{\pm 0.1359}$	0.6086${}_{\pm 0.1190}$	0.6288${}_{\pm 0.1167}$	0.6521${}_{\pm 0.1097}$
	EDL	0.5244${}_{\pm 0.0179}$	0.6015${}_{\pm 0.0335}$	0.6289${}_{\pm 0.0464}$	0.5639${}_{\pm 0.0223}$	0.5975${}_{\pm 0.0156}$
	SWAG	0.5070${}_{\pm 0.0303}$	0.5694${}_{\pm 0.0377}$	0.6111${}_{\pm 0.0713}$	0.5556${}_{\pm 0.0514}$	0.5669${}_{\pm 0.0406}$
	MCD	0.4932${}_{\pm 0.0208}$	0.9715${}_{\pm 0.3642}$	0.5175${}_{\pm 0.0741}$	0.5311${}_{\pm 0.0158}$	0.6710${}_{\pm 0.2239}$
	DKL	0.5526${}_{\pm 0.0384}$	0.6982${}_{\pm 0.1190}$	0.6275${}_{\pm 0.0775}$	0.5972${}_{\pm 0.0541}$	0.5727${}_{\pm 0.0530}$
	SGLD	0.8403${}_{\pm 0.1110}$	**0.5572** ${}_{\pm 0.0076}$	**0.4611** ${}_{\pm 0.0085}$	0.5275${}_{\pm 0.0061}$	**0.5389** ${}_{\pm 0.0020}$

**Table 3:** Brier score of different models with sequence level features from different PLMs.

Dataset	Model	ESMC	ProstT5	Ankh	ESM2	Prot Bert
Virus	Deterministic	0.0746${}_{\pm 0.0062}$	0.0852${}_{\pm 0.0047}$	0.0844${}_{\pm 0.0084}$	0.0796${}_{\pm 0.0047}$	0.0821${}_{\pm 0.0010}$
	TS	0.0743${}_{\pm 0.0061}$	0.0842${}_{\pm 0.0044}$	0.0833${}_{\pm 0.0072}$	**0.0794** ${}_{\pm 0.0047}$	0.0822${}_{\pm 0.0015}$
	LA	**0.0725** ${}_{\pm 0.0025}$	0.0845${}_{\pm 0.0105}$	0.0855${}_{\pm 0.0092}$	0.0852${}_{\pm 0.0039}$	0.0823${}_{\pm 0.0063}$
	DVBLL	0.0775${}_{\pm 0.0088}$	0.0876${}_{\pm 0.0105}$	0.1504${}_{\pm 0.0811}$	0.1100${}_{\pm 0.0419}$	0.0792${}_{\pm 0.0048}$
	EDL	0.1154${}_{\pm 0.0543}$	**0.0772** ${}_{\pm 0.0035}$	0.0824${}_{\pm 0.0037}$	0.1125${}_{\pm 0.0263}$	0.0845${}_{\pm 0.0065}$
	SWAG	0.0914${}_{\pm 0.0098}$	0.0852${}_{\pm 0.0102}$	0.1078${}_{\pm 0.0204}$	0.0962${}_{\pm 0.0093}$	0.0911${}_{\pm 0.0106}$
	MCD	0.0745${}_{\pm 0.0062}$	0.0849${}_{\pm 0.0047}$	0.0842${}_{\pm 0.0081}$	0.0796${}_{\pm 0.0047}$	0.0821${}_{\pm 0.0010}$
	DKL	0.0892${}_{\pm 0.0113}$	0.1232${}_{\pm 0.0325}$	0.1174${}_{\pm 0.0608}$	0.0853${}_{\pm 0.0208}$	0.1096${}_{\pm 0.0408}$
	SGLD	0.0852${}_{\pm 0.0017}$	0.0836${}_{\pm 0.0030}$	**0.0765** ${}_{\pm 0.0018}$	0.0854${}_{\pm 0.0013}$	**0.0819** ${}_{\pm 0.0009}$
Bacteria	Deterministic	0.1503${}_{\pm 0.0124}$	0.1642${}_{\pm 0.0113}$	0.1521${}_{\pm 0.0028}$	0.1403${}_{\pm 0.0095}$	0.1510${}_{\pm 0.0055}$
	TS	0.1484${}_{\pm 0.0130}$	0.1601${}_{\pm 0.0103}$	0.1483${}_{\pm 0.0029}$	0.1383${}_{\pm 0.0081}$	0.1485${}_{\pm 0.0048}$
	LA	0.1487${}_{\pm 0.0073}$	0.1656${}_{\pm 0.0141}$	0.1565${}_{\pm 0.0063}$	0.1499${}_{\pm 0.0169}$	0.1509${}_{\pm 0.0080}$
	DVBLL	0.1587${}_{\pm 0.0148}$	0.1685${}_{\pm 0.0158}$	0.2024${}_{\pm 0.0378}$	0.1480${}_{\pm 0.0082}$	0.1424${}_{\pm 0.0034}$
	EDL	**0.1376** ${}_{\pm 0.0055}$	0.1597${}_{\pm 0.0348}$	0.1696${}_{\pm 0.0193}$	0.1840${}_{\pm 0.0542}$	0.1472${}_{\pm 0.0271}$
	SWAG	0.1376${}_{\pm 0.0111}$	**0.1460** ${}_{\pm 0.0074}$	0.1488${}_{\pm 0.0188}$	**0.1358** ${}_{\pm 0.0078}$	0.1447${}_{\pm 0.0098}$
	MCD	0.1502${}_{\pm 0.0124}$	0.1636${}_{\pm 0.0112}$	0.1515${}_{\pm 0.0029}$	0.1402${}_{\pm 0.0093}$	0.1506${}_{\pm 0.0056}$
	DKL	0.1546${}_{\pm 0.0056}$	0.1653${}_{\pm 0.0093}$	0.1592${}_{\pm 0.0117}$	0.1601${}_{\pm 0.0204}$	0.1488${}_{\pm 0.0065}$
	SGLD	0.1623${}_{\pm 0.0040}$	0.1473${}_{\pm 0.0006}$	**0.1406** ${}_{\pm 0.0015}$	0.1373${}_{\pm 0.0007}$	**0.1327** ${}_{\pm 0.0013}$
Tumor	Deterministic	0.1675${}_{\pm 0.0090}$	0.2255${}_{\pm 0.0248}$	0.1687${}_{\pm 0.0231}$	0.1703${}_{\pm 0.0065}$	0.2040${}_{\pm 0.0574}$
	TS	0.1674${}_{\pm 0.0090}$	0.2197${}_{\pm 0.0222}$	0.1661${}_{\pm 0.0209}$	**0.1672** ${}_{\pm 0.0067}$	0.1981${}_{\pm 0.0508}$
	LA	**0.1632** ${}_{\pm 0.0128}$	0.2221${}_{\pm 0.0245}$	0.2006${}_{\pm 0.0173}$	0.1841${}_{\pm 0.0169}$	0.1892${}_{\pm 0.0146}$
	DVBLL	0.1753${}_{\pm 0.0133}$	0.2441${}_{\pm 0.0222}$	0.1925${}_{\pm 0.0410}$	0.1859${}_{\pm 0.0220}$	0.2014${}_{\pm 0.0226}$
	EDL	0.1727${}_{\pm 0.0074}$	0.2069${}_{\pm 0.0150}$	0.2192${}_{\pm 0.0220}$	0.1909${}_{\pm 0.0093}$	0.2054${}_{\pm 0.0071}$
	SWAG	0.1652${}_{\pm 0.0099}$	0.1880${}_{\pm 0.0115}$	0.1809${}_{\pm 0.0139}$	0.1827${}_{\pm 0.0172}$	**0.1828** ${}_{\pm 0.0070}$
	MCD	0.1676${}_{\pm 0.0086}$	0.2246${}_{\pm 0.0243}$	0.1680${}_{\pm 0.0230}$	0.1698${}_{\pm 0.0069}$	0.2031${}_{\pm 0.0559}$
	DKL	0.1800${}_{\pm 0.0140}$	0.2232${}_{\pm 0.0270}$	0.2052${}_{\pm 0.0151}$	0.2043${}_{\pm 0.0241}$	0.1931${}_{\pm 0.0237}$
	SGLD	0.2379${}_{\pm 0.0193}$	**0.1862** ${}_{\pm 0.0010}$	**0.1530** ${}_{\pm 0.0032}$	0.1806${}_{\pm 0.0029}$	0.1833${}_{\pm 0.0007}$

### ID (in-distribution) results

**Figure 2:**
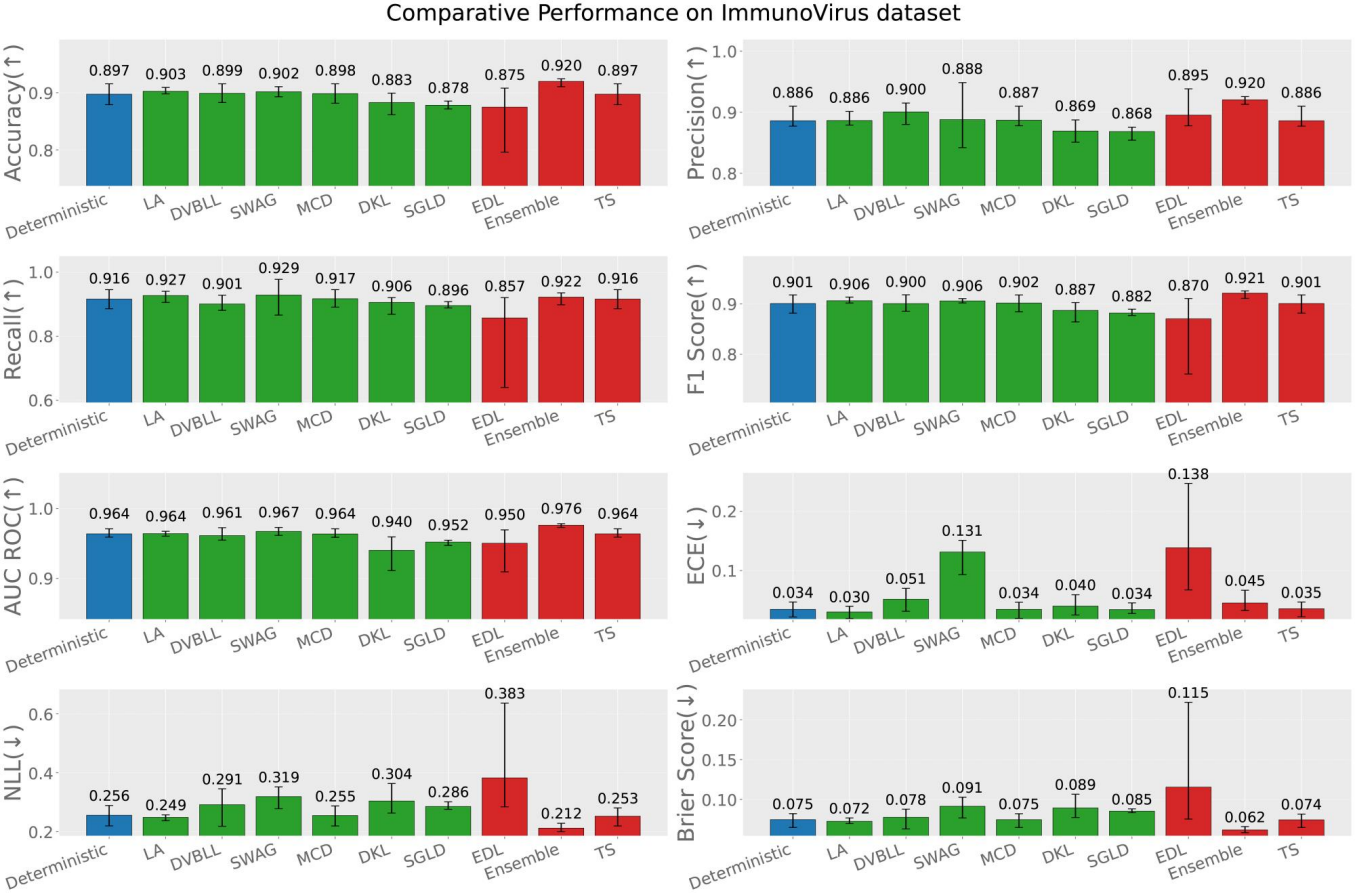
In-Distribution results for Immuno-Virus dataset. For the predictive metrics (accuracy, precision, recall, F1-Score, AUC ROC), the optimal performance indicates the higher the better; whereas for the UQ metrics (ECE, NLL, brier score), the optimal performance indicates the lower the better. The values on each bar plotted on all the subplots show the mean of the values, and the error bars show the range of values obtained from all the different experimental seeds. The deterministic, Bayesian, and non-Bayesian methods are plotted in blue, green and red, respectively.

**Figure 3:**
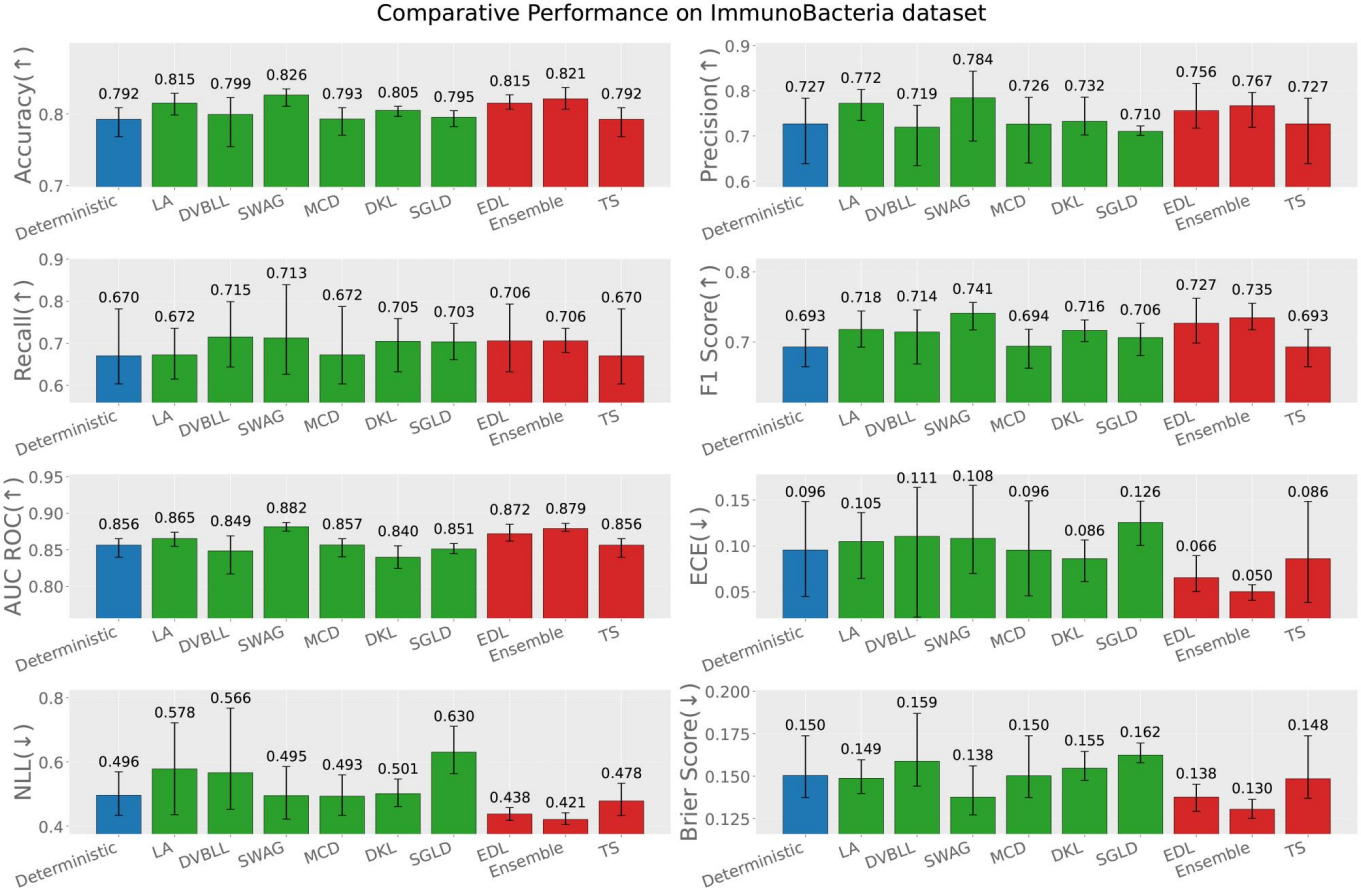
In-Distribution results for Immuno-Bacteria dataset. For the predictive metrics (accuracy, precision, recall, F1-Score, AUC ROC), the optimal performance indicates the higher the better; whereas for the UQ metrics (ECE, NLL, brier score), the optimal performance indicates the lower the better. The values on each bar plotted on all the subplots show the mean of the values, and the error bars show the range of values obtained from all the different experimental seeds. The deterministic, Bayesian, and non-Bayesian methods are plotted in blue, green, and red, respectively.

**Figure 4:**
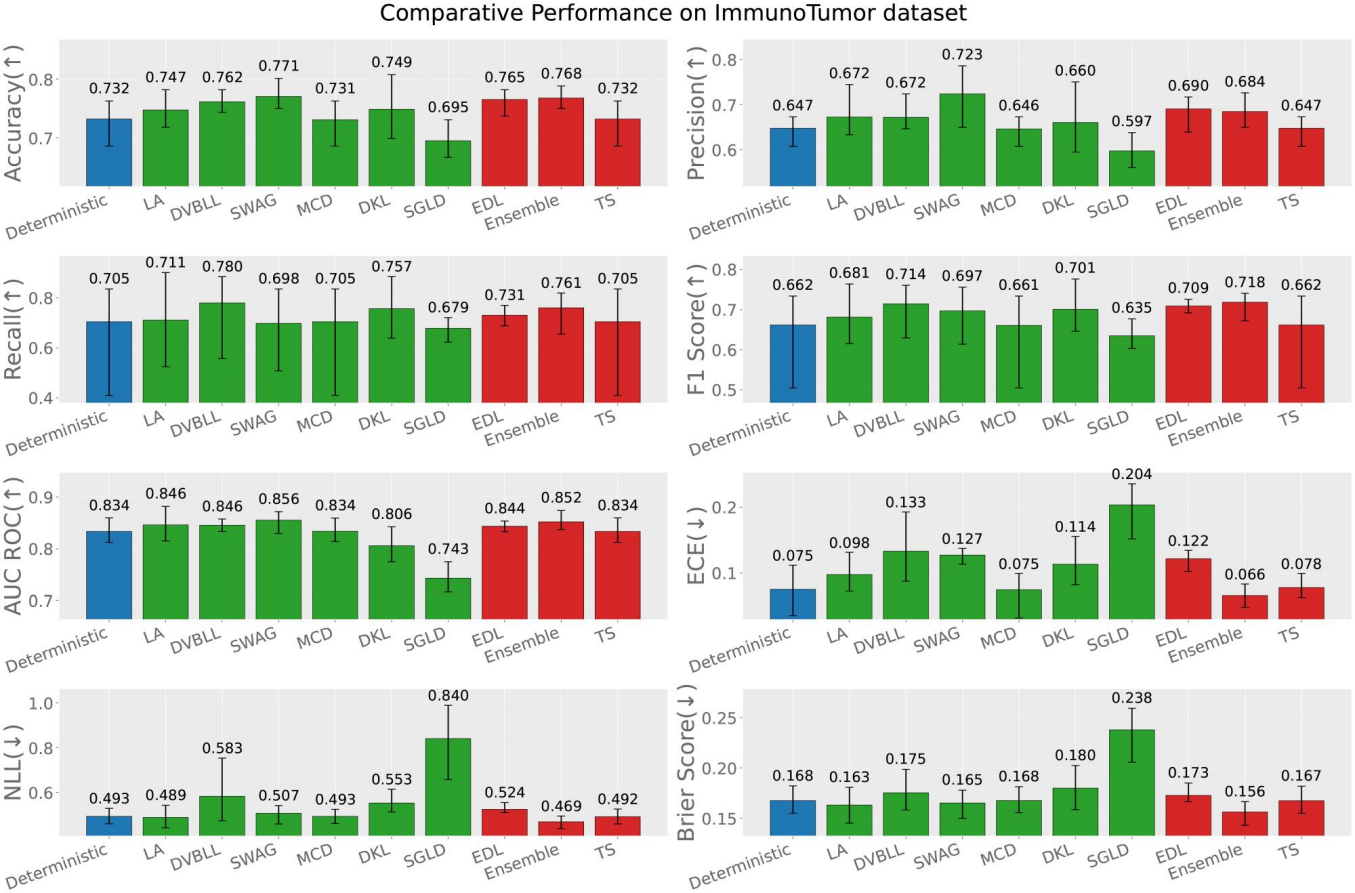
In-Distribution results for Immuno-Tumor dataset. For the predictive metrics (accuracy, precision, recall, F1-Score, AUC ROC), the optimal performance indicates the higher the better; whereas for the UQ metrics (ECE, NLL, brier score), the optimal performance indicates the lower the better. The values on each bar plotted on all the subplots show the mean of the values, and the error bars show the range of values obtained from all the different experimental seeds. The deterministic, Bayesian, and non-Bayesian methods are plotted in blue, green and red, respectively.

In-distribution evaluation refers to the scenario where the train and test sets both originate from the same immunogenic data source, e.g. virus, bacteria or tumor (test data source = train data source). Figures 2–4 rank the performance of different models according the performance across different predictive and UQ metrics.

For each of the three immunogenic datasets, generally UQ methods showed superior performances compared to the deterministic model across nearly all performance and uncertainty metrics. The superior performance is more obvious in Bacteria dataset, underscoring the benefits of uncertainty-aware modeling. A more detailed explanation is provided in the following.

For Immuno-Virus dataset, the Ensemble model outperforms other models in terms of all metrics except Recall and ECE where SWAG and LA yield the best performance and calibrated predictions respectively.

In evaluating the Immuno-Bacteria dataset, SWAG stands out for its dominant predictive performance, achieving the best results across all relevant metrics. However, in terms of uncertainty quantification, the Ensemble model provides the most reliable uncertainty quantification, as reflected in its leading ECE, NLL, and Brier score results.

For the Immuno-Tumor dataset, SWAG, and Ensemble consistently demonstrate the most effective predictive performance, achieving the highest scores across all performance metrics. However, regarding uncertainty quantification, the Ensemble model, similar to Bacteria dataset, showed the superior performance over all uncertainty metrics.

**Figure 5:**
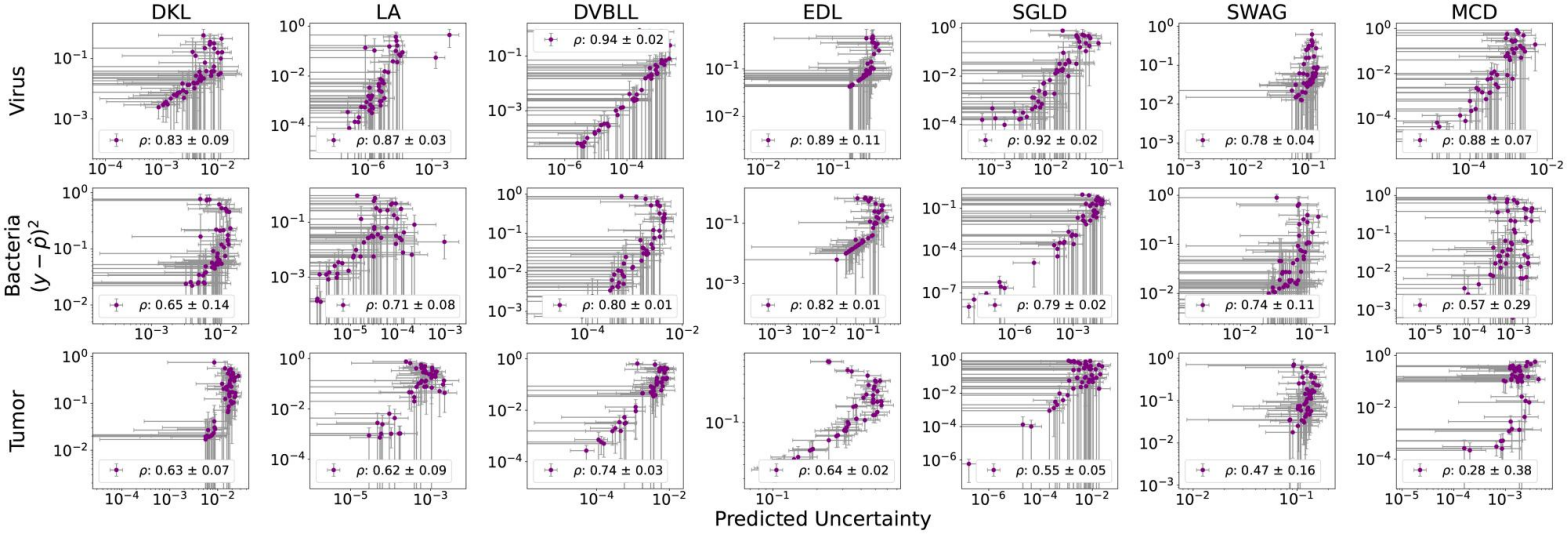
Spearman correlation, ρ between squared error and the predicted uncertainty. In nearly all methods the predicted uncertainty has a high correlation with error, with DVBLL, EDL, and LA demonstrating a more consistent performance across datasets.

Finally, we have examined the correlation between the squared error (the difference between the true label and the predicted probability) and the predicted uncertainties for each probabilistic method. Figure 5 summarizes the Spearman correlations across all models. Overall, predicted uncertainties are strongly correlated with prediction error for most methods and datasets, with the exception of the Tumor dataset, where SWAG and MCD exhibit noticeably weaker correlations. The Virus dataset shows the highest correlations across all models, indicating that uncertainty estimates are most reliable in this setting. On the Bacteria dataset, correlations remain high for nearly all methods (except MCD), but are consistently lower than those observed for Virus. The Tumor dataset yields the weakest correlations overall, highlighting a greater challenge in aligning uncertainty estimates with predictive accuracy. Among the methods, MCD is particularly sensitive to the dataset: while it achieves strong correlations on Virus, its performance degrades substantially on Tumor. This underscores how the quality of the posterior approximation can depend on the dataset, potentially due to the restricted expressiveness of the variational family. In contrast, LA, DVBLL, and EDL show a more consistent performance across all datasets. Interestingly, these methods consistently show outstanding predictive performance, but they do not necessarily achieve the best UQ performance as reported in Figs. 2–4.

In summary, among all datasets, the Ensemble model consistently showed a superior uncertainty quantification capability, while having comparable performance to the best performing models. Also, over all metrics, probabilistic models outperformed their deterministic alternative as expected.

### OOD (out-of-distribution) results

**Figure 6:**
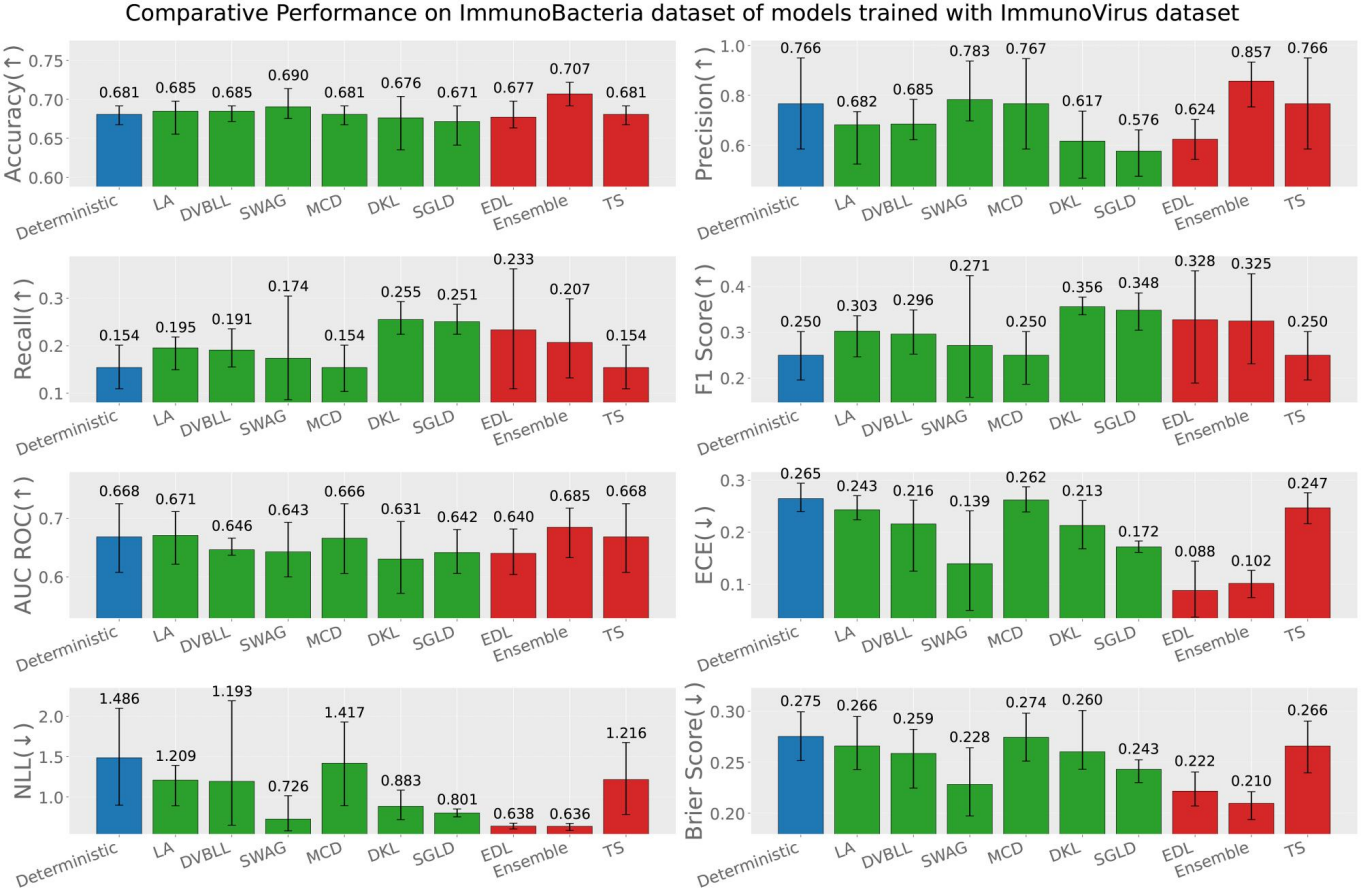
Out-of-distribution results on Immuno-Bacteria dataset of models trained with Immuno-Virus dataset. For the predictive metrics (accuracy, precision, recall, F1-Score, AUC ROC), the optimal performance indicates the higher the better; whereas for the UQ metrics (ECE, NLL, brier score), the optimal performance indicates the lower the better. The values on each bar plotted on all the subplots show the mean of the values, and the error bars show the range of values obtained from all the different experimental seeds. The deterministic, Bayesian, and non-Bayesian methods are plotted in blue, green, and red, respectively.

**Figure 7:**
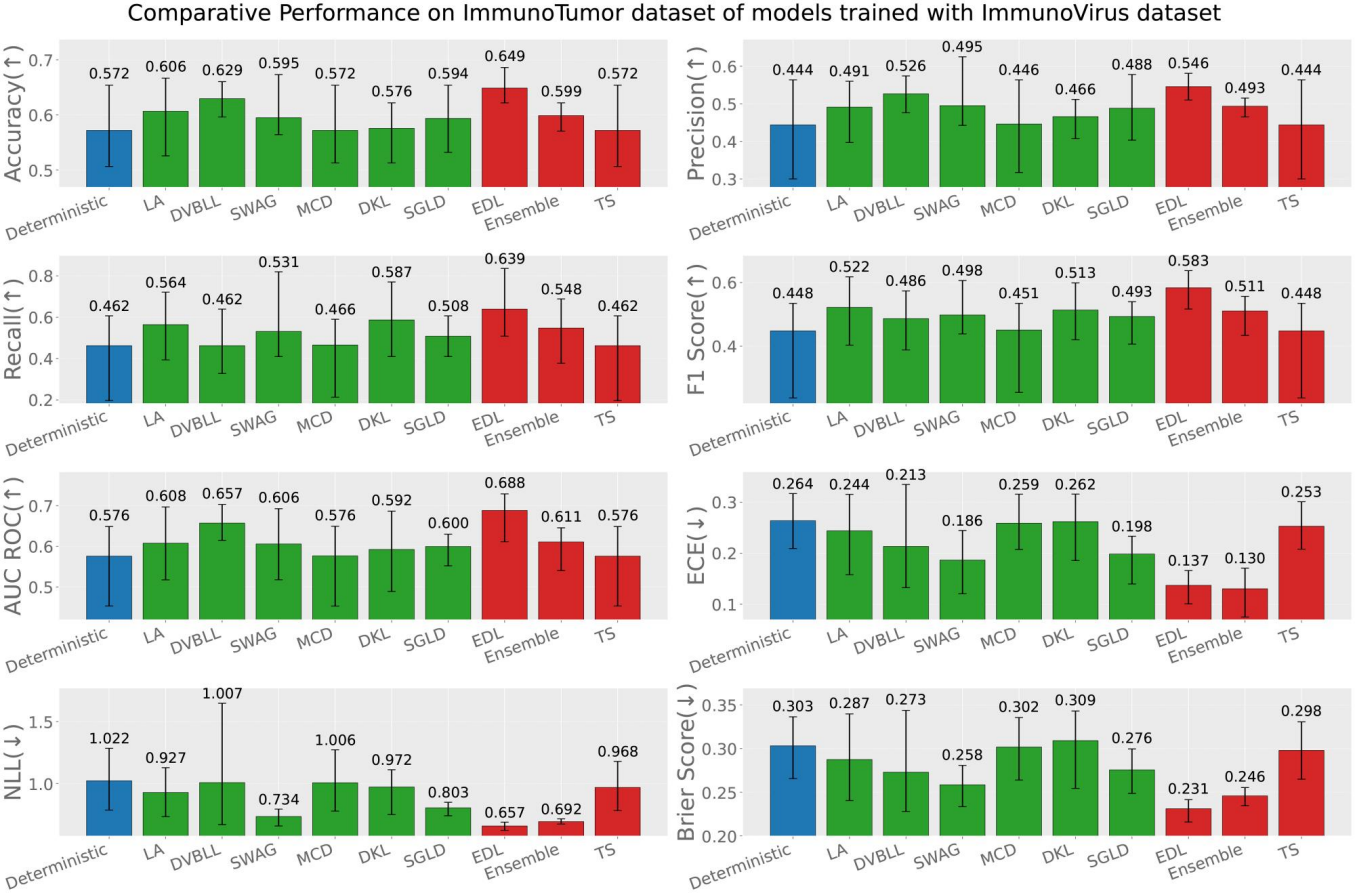
Out-of-distribution results on Immuno-Tumor dataset of models trained with Immuno-Virus dataset. For the predictive metrics (accuracy, precision, recall, F1-Score, AUC ROC), the optimal performance indicates the higher the better; whereas for the UQ metrics (ECE, NLL, brier score), the optimal performance indicates the lower the better. The values on each bar plotted on all the subplots show the mean of the values, and the error bars show the range of values obtained from all the different experimental seeds. The deterministic, Bayesian, and non-Bayesian methods are plotted in blue, green, and red, respectively.

**Figure 8:**
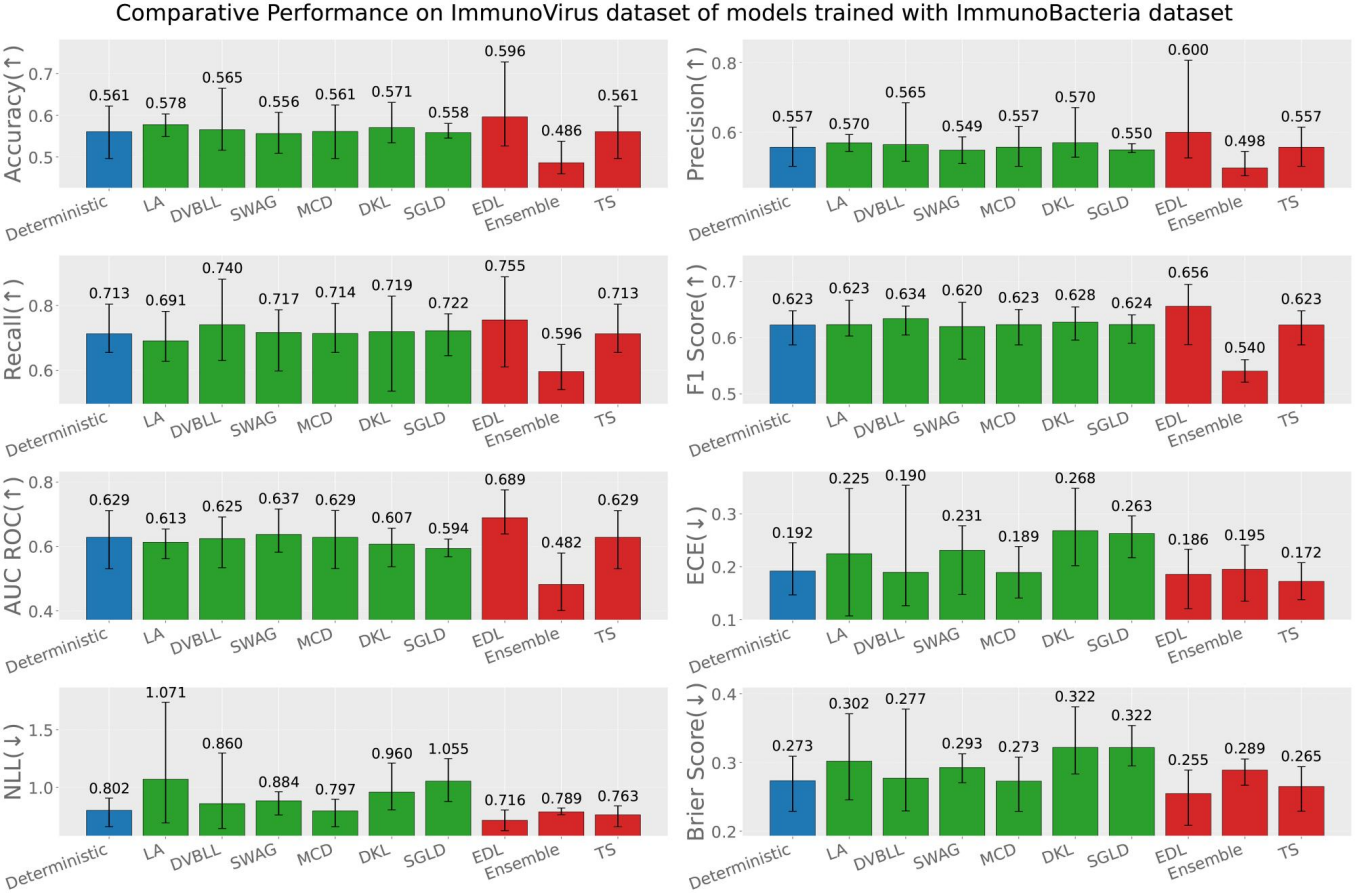
Out-of-distribution results on Immuno-Virus dataset of models trained with Immuno-Bacteria dataset. For the predictive metrics (accuracy, precision, recall, F1-Score, AUC ROC), the optimal performance indicates the higher the better; whereas for the UQ metrics (ECE, NLL, brier score), the optimal performance indicates the lower the better. The values on each bar plotted on all the subplots show the mean of the values, and the error bars show the range of values obtained from all the different experimental seeds. The deterministic, Bayesian, and non-Bayesian methods are plotted in blue, green, and red, respectively.

**Figure 9:**
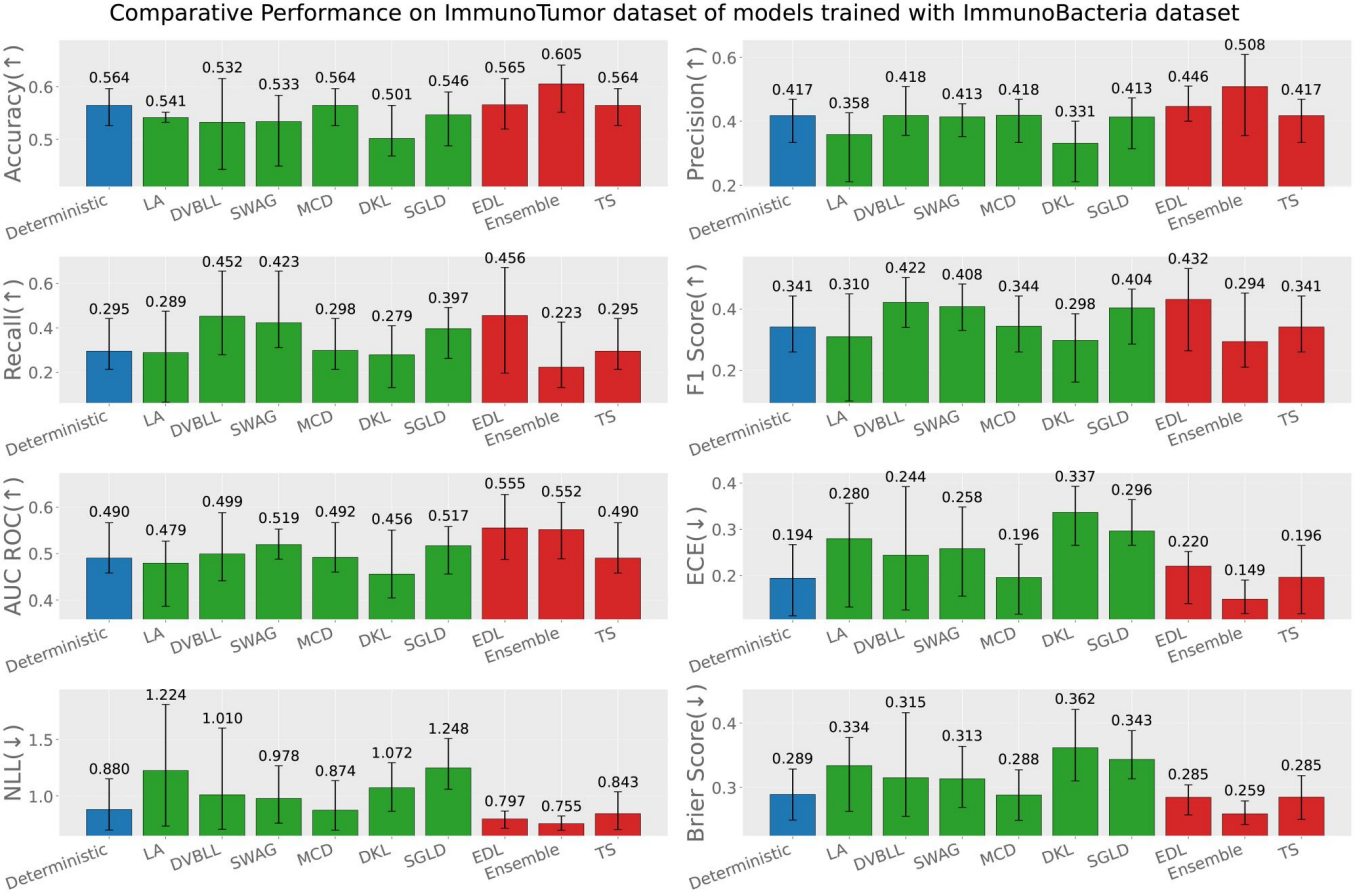
Out-of-distribution results on Immuno-Tumor dataset of models trained with Immuno-Bacteria dataset. For the predictive metrics (accuracy, precision, recall, F1-Score, AUC ROC), the optimal performance indicates the higher the better; whereas for the UQ metrics (ECE, NLL, brier score), the optimal performance indicates the lower the better. The values on each bar plotted on all the subplots show the mean of the values, and the error bars show the range of values obtained from all the different experimental seeds. The deterministic, Bayesian, and non-Bayesian methods are plotted in blue, green, and red, respectively.

**Figure 10:**
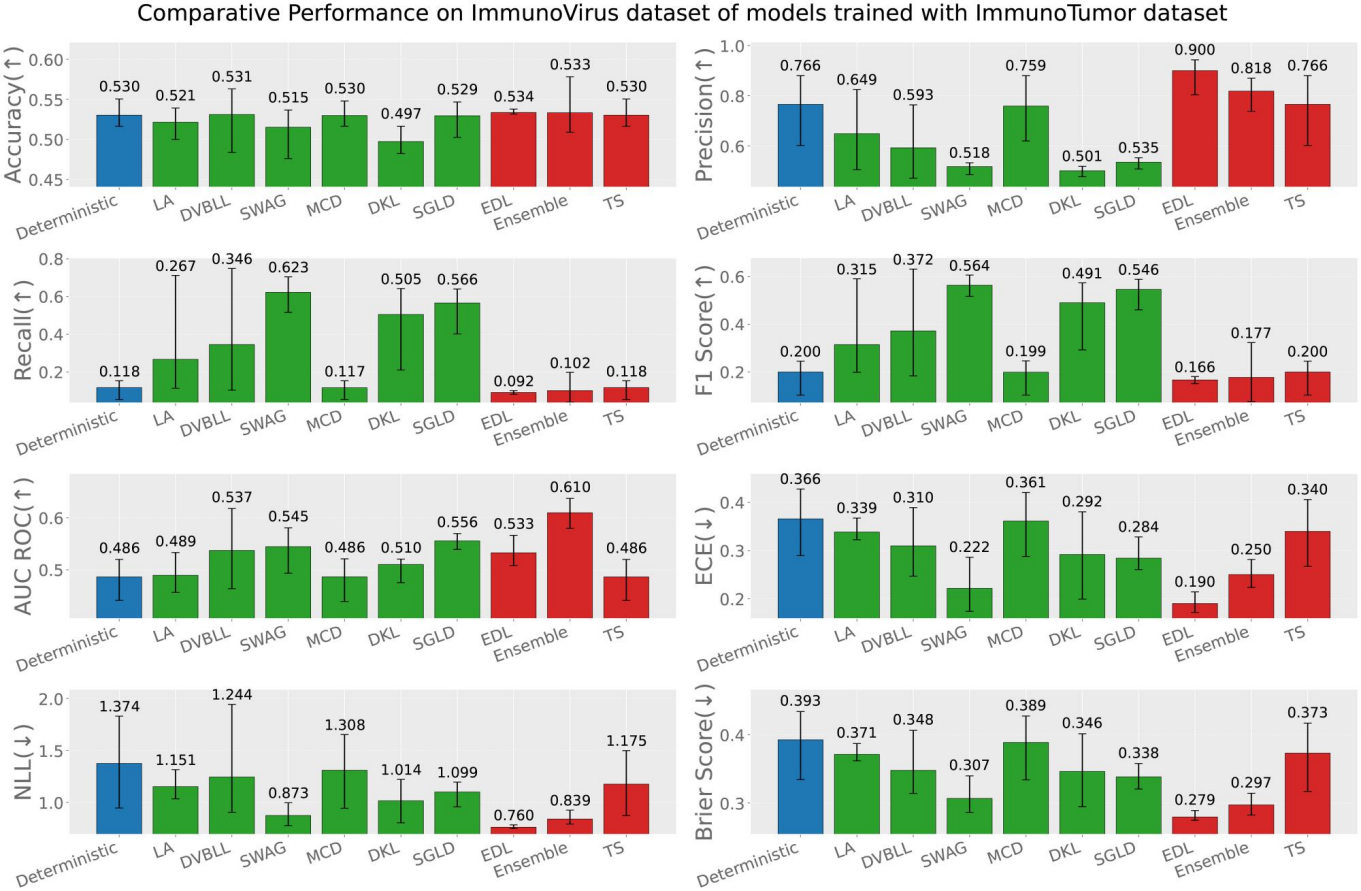
Out-of-distribution results on Immuno-Virus dataset of models trained with Immuno-Tumor dataset. For the predictive metrics (accuracy, precision, recall, F1-Score, AUC ROC), the optimal performance indicates the higher the better; whereas for the UQ metrics (ECE, NLL, brier score), the optimal performance indicates the lower the better. The values on each bar plotted on all the subplots show the mean of the values, and the error bars show the range of values obtained from all the different experimental seeds. The deterministic, Bayesian, and non-Bayesian methods are plotted in blue, green, and red, respectively.

**Figure 11:**
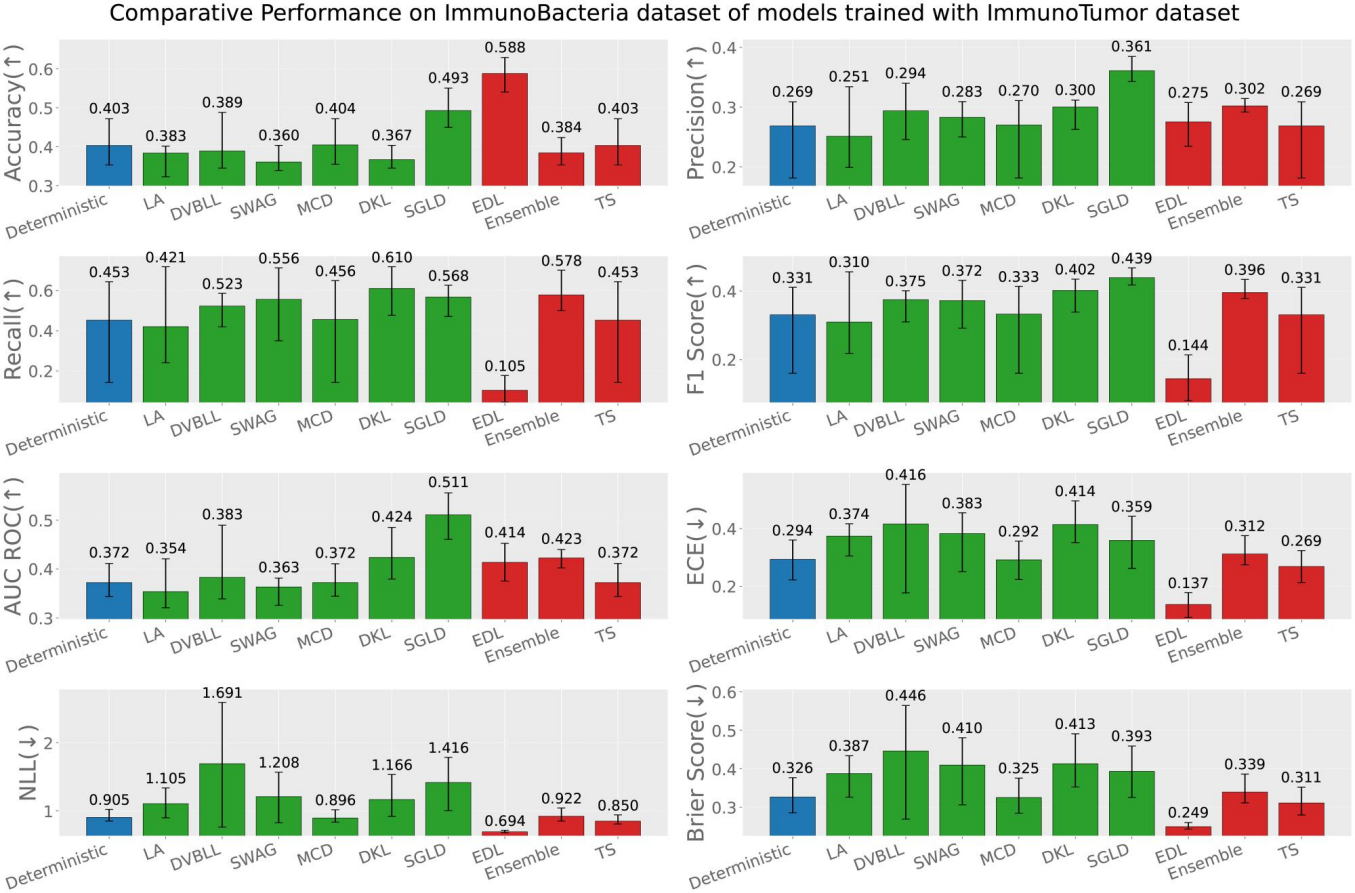
Out-of-distribution results on Immuno-Bacteria dataset of models trained with Immuno-Tumor dataset. For the predictive metrics (accuracy, precision, recall, F1-Score, AUC ROC), the optimal performance indicates the higher the better; whereas for the UQ metrics (ECE, NLL, brier score), the optimal performance indicates the lower the better. The values on each bar plotted on all the subplots show the mean of the values, and the error bars show the range of values obtained from all the different experimental seeds. The deterministic, Bayesian, and non-Bayesian methods are plotted in blue, green, and red, respectively.

Apart from the evaluation of models on test datasets, trained on respective data sources; we also report the out-of-distribution evaluation results based on the following three settings (test data source ≠ train data source). Figures 6–11 show the evaluative results for different OOD evaluative scenarios with visualizations which show the rank of different models based on their respective performance on that metric. Values displayed on top of each bar show the mean of values and the error bars show the range of values obtained from all the different experimental seeds.

**Generalization performance of models trained on immuno-virus dataset**: Figs. 6 and 7 show the evaluation of models on Immuno-Bacteria and Immuno-Tumor datasets accordingly, while they were trained on Immuno-Virus dataset. Overall, the results underscore the superiority of probabilistic models, with most such methods achieving better scores than deterministic alternatives on almost all performance and uncertainty metrics. In detail, Ensemble and SGLD show superior predictive performance on Bacteria dataset under OOD setting, while EDL and Ensemble outperform other methods concerning the uncertainty quantification metrics. Interestingly, unlike the ID setting, Ensemble model lagged behind other methods in both predictive and uncertainty metrics on Tumor dataset under OOD setting, except for ECE, where it outperformed other methods. EDL shows strong uncertainty quantification depicted by its superior performance on uncertainty metrics, while with respect to predictive performance, EDL, SVDKL, and DVBLL outperform other methods depending on the metric. In summary, under OOD setting, when the models are trained on Immuno-Virus dataset, generally EDL, SWAG, and Ensemble show a superior performance to other models.

**Generalization performance of models trained on immuno-bacteria dataset**: Figs. 8 and 9 show the evaluation of models on Immuno-Virus and Immuno-Tumor datasets accordingly, while they were trained on Immuno-Bacteria dataset. In both datasets, EDL and Ensemble consistently show a superior performance over other methods. Particularly, in Virus dataset, EDL outperforms all other methods with respect to all performance and uncertainty metrics except for ECE where TS has the best performance. On Tumor dataset, Ensemble and EDL outperform all methods with respect to performance metrics, depending on the metric. Concerning the uncertainty quantification, Ensemble has the best uncertainty quantification performance, evidenced by its superior performance across all uncertainty metrics.

**Generalization performance of models trained on immuno-tumor dataset**: Figs. 10 and 11 show the evaluation of models on Immuno-Virus and Immuno-Bacteria datasets accordingly, while they were trained on Immuno-Tumor dataset. On both datasets, EDL consistently shows a superior uncertainty quantification performance depicted over all uncertainty metrics. However, concerning the predictive performance, it is more difficult to draw any conclusions about the best performing model. In detail, on Virus dataset, Ensemble, SWAG and EDL show a superior performance over other methods depending on the metric. On Bacteria dataset, SGLD more consistently show a better predictive performance, outperforming other methods with respect to most metrics but Accuracy and Recall, where EDL and SVDKL outperform other methods respectively.

**Summary on OOD performance**: in summary, Ensemble and EDL show a greater consistency and robustness in uncertainty quantification under OOD setting. Concerning the predictive performance, although EDL, SWAG, and Ensemble depict a superior performance on different datasets, there is no method clearly outperforming others under OOD setting. However, the clear conclusion to draw from these results is that the deterministic model is almost always outperformed by probabilistic methods, highlighting its limited generalizability and reliability.

### PLM embedding comparison

Our ImmUQBench platform facilitates the evaluation and comparison of not only different UQ strategies, but also different model components for immunogenicity prediction. As part of this, we evaluate embeddings extracted from different protein language models that have been developed and improved by different research groups.

Tables 1–3 show the comparative performance among models with different PLMs for extracting amino acid sequence embeddings.

Across all three immunogenic datasets, and for nearly every performance metric considered, deterministic models consistently demonstrated inferior performance compared to other models. The only exception to this trend was observed on the Immuno-Virus dataset when protein sequences were embedded using the ESM-2 PLM, concerning the ECE and Brier score metric, and on the Immuno-Tumor dataset with ESM-2 embedded sequences, concerning NLL and Brier score. Among the various models investigated, SGLD consistently emerged as a strong performer. Plus SGLD, SWAG, EDL, and LA also exhibited competitive results, suggesting their potential for accurate immunogenicity prediction. To summarize, advantages over uncertainty-aware models over deterministic models persist irrespective of the protein language model used, underscoring compatibility with advances in protein representation learning.

### Discussion

All the abovementioned experimental results consistently show that uncertainty-aware models outperform deterministic baselines in immunogenicity prediction, offering gains in both predictive accuracy and calibration. These improvements were observed across all in-distribution datasets and persisted across different protein language model embeddings, suggesting that the benefits of UQ are largely independent of the upstream sequence representation. Enhanced calibration, most notably achieved by SWAG, EDL, LA, and Ensemble, has particular relevance in high-stakes biomedical applications, where overconfident errors can result in costly experimental misallocation or safety risks.

Performance differences became more nuanced across the out-of-distribution evaluation scenarios. EDL and Ensemble demonstrated strong calibration robustness under distribution shift, whereas SWAG, SGLD, and Ensembles along EDL often achieved higher predictive performance in specific scenarios. This indicates a trade-off between reliability and sharpness that should be aligned with downstream objectives.

Overall, empirical findings establish UQ as a means of achieving more reliable and effective immunogenicity prediction and provide actionable guidance for selecting model–task configurations in both experimental and clinical settings.

## Related works

**Protein language models:** Advances in deep learning and the emergence of large language models, including specialized protein language models (PLMs), have revolutionized computational biology by offering accurate, generalizable, and scalable solutions to complex downstream tasks such as vaccinology, drug discovery, immunogenicity prediction, and therapeutic design. DeepNetBim [4] employs a hybrid architecture combining convolutional neural networks with attention mechanisms to integrate sequence features and network centrality metrics for predicting HLA–peptide binding affinity and immunogenicity. ImmugenX [53] introduces a modular PLM-based pipeline to predict immunogenic CD8^+^ epitopes, a task central to personalized immunotherapy. DeepHLApan [54] uses bi-directional GRUs with attention to jointly model HLA–peptide binding and immunogenicity for neoantigen discovery. UnifyImmun [55] adopts a transformer-based framework with dual encoders and cross-attention to simultaneously model HLA–peptide and peptide–TCR interactions, offering improved generalization and interpretability.

**Uncertainty quantification**: Despite the success of deep learning and large language models (LLMs) across numerous domains, these models often suffer from overconfident predictions and, in the case of LLMs, hallucinations. This has motivated the development of uncertainty quantification (UQ) techniques to assess the reliability of model outputs. Subspace Inference [56] constructs Bayesian posteriors in low-dimensional subspaces of model parameters, enabling efficient inference and calibrated uncertainty estimates. Contextual Dropout [57] learns data-dependent dropout probabilities, offering both improved predictive performance and uncertainty estimation. Laplace-LoRA [58] applies Laplace approximation over low-rank adaptation parameters in a post hoc manner, allowing for efficient posterior estimation after fine-tuning. BLoB [59] formulates a Bayesian low-rank adaptation framework by jointly estimating mean and covariance during fine-tuning. Contextual LoRA [60] further extends this by incorporating contextual uncertainty modules that dynamically adjust aleatoric uncertainty on a per-sample basis.

**Existing benchmarks**: Several benchmark studies have been developed to evaluate the performance of PLMs across a wide range of biological tasks, offering insights into their generalization and transfer learning capabilities. However, few benchmarks have explicitly investigated their behavior under uncertainty or assessed their reliability in critical biomedical applications. PEER [8] provides a comprehensive multi-task evaluation framework across protein function, localization, structure, and molecular interaction tasks, comparing traditional methods and PLMs. PETA [61] evaluates 13 PLMs with varying vocabulary sizes and tokenization strategies across 15 downstream tasks, shedding light on the impact of subword versus. amino-acid-level tokenization on PLM performance. The authors of [62] benchmarked multiple UQ methods on protein fitness regression tasks under various distributional shifts, revealing key trade-offs between calibration, accuracy, and data efficiency.

## Conclusion & limitations

**Conclusion**: In this study, we have introduced ImmUQBench, a new benchmark for evaluating a range of uncertainty quantification (UQ) methods on the task of immunogenicity prediction. This benchmark demonstrates that UQ methods deliver benefits extending beyond calibration, consistently enhancing predictive performance across diverse datasets and embedding strategies. By leveraging multi-modal information and incorporating it through a state-of-the-art backbone model, our benchmark enables comprehensive evaluation under both in-distribution and out-of-distribution settings. Our results demonstrate that most UQ methods consistently outperform the deterministic baseline across various metrics. In particular, Ensemble, EDL, SWAG, and Laplace Approximation (LA) exhibit superior performance in terms of predictive accuracy, uncertainty estimation, and generalization. While some UQ methods occasionally underperform relative to the deterministic model, overall, UQ-based approaches yield more robust and calibrated predictions, especially in out-of-distribution scenarios. ImmUQBench—to the best of our knowledge, the first UQ benchmark for immunogenicity prediction—offers a valuable resource for developing more reliable and uncertainty-aware antigen design tools.

**Limitations & future work**: Our proposed ImmUQBench provides a targeted evaluation of selected Bayesian and non-Bayesian UQ methods. However, it does not explore the broader design space, including variations in backbone architectures—e.g. PLGDL which integrates both Protein Language Models and Geometric Deep Learning models [63]—or uncertainty propagation beyond the prediction head—e.g. integrate uncertainty in protein representation [64] into prediction of protein properties. Also, the current benchmark only addresses epistemic uncertainty; the consideration of uncertainty within protein representations and its effect on immunogenicity prediction still remains an open research endeavor.

## Supplementary Material

iqag003_Supplementary_Data

## Data Availability

Data and code implementations can be accessed at https://github.com/alifbinabdulqayyum/ImmUQBench.

## References

[1] Doneva N , Doytchinova I, Dimitrov I. Predicting immunogenicity risk in biopharmaceuticals. Symmetry 2021;13:388.

[2] Adu-Bobie J , Capecchi B, Serruto D et al Two years into reverse vaccinology. Vaccine 2003;21:605–10. 12531326 10.1016/s0264-410x(02)00566-2

[3] Li G , Iyer B, Prasath VBS et al Deepimmuno: deep learning-empowered prediction and generation of immunogenic peptides for t-cell immunity. Brief Bioinform 2021;22:bbab160. 34009266 10.1093/bib/bbab160 PMC8135853

[4] Yang X , Zhao L, Wei F, Li J. DeepNetBim: deep learning model for predicting HLA-epitope interactions based on network analysis by harnessing binding and immunogenicity information. BMC Bioinform 2021;22:231.May 10.1186/s12859-021-04155-y PMC8097772 33952199

[5] Touvron H , Lavril T, Izacard G et al Llama: Open and efficient foundation language models, 2023.

[6] Devlin J , Chang M-W, Lee K et al Bert: pre-training of deep bidirectional transformers for language understanding, 2019.

[7] Brown TB , Mann B, Ryder N et al Language models are few-shot learners, 2020.

[8] Xu M , Zhang Z, Lu J et al Peer: a comprehensive and multi-task benchmark for protein sequence understanding, 2022.

[9] ESM Team. ESM Cambrian: revealing the mysteries of proteins with unsupervised learning. https://evolutionaryscale.ai/blog/esm-cambrian, December 2024. EvolutionaryScale Website.

[10] Ferruz N , Schmidt S, Höcker B. ProtGPT2 is a deep unsupervised language model for protein design. Nat Commun 2022;13:4348.July 35896542 10.1038/s41467-022-32007-7 PMC9329459

[11] Gawlikowski J , Tassi CRN, Ali M et al A survey of uncertainty in deep neural networks, 2022.

[12] Shorinwa O , Mei Z, Lidard J et al A survey on uncertainty quantification of large language models: taxonomy, open research challenges, and future directions, 2025.

[13] Chen T , Fox EB, Guestrin C. Stochastic gradient Hamiltonian Monte Carlo, 2014.

[14] Ding N , Fang Y, Babbush R et al Bayesian sampling using stochastic gradient thermostats. In *Proceedings of the 28th International Conference on Neural Information Processing Systems - Volume 2*, NIPS’14, p.3203–3211. Cambridge, MA: MIT Press, 2014.

[15] Gal Y , Ghahramani Z. Dropout as a bayesian approximation: representing model uncertainty in deep learning. In: Balcan MF, Weinberger KQ (ed.), *Proceedings of the 33rd International Conference on Machine Learning*, volume 48 of *Proceedings of Machine Learning Research*. New York, NY: PMLR, 20–22 Jun 2016, 1050–1059.

[16] Amini A , Schwarting W, Soleimany A, Rus D. Deep evidential regression, 2020.

[17] Cortes-Gomez S , Patiño CM, Byun Y et al Utility-directed conformal prediction: a decision-aware framework for actionable uncertainty quantification. In *The Thirteenth International Conference on Learning Representations*, 2025.

[18] Jain S , Liu G, Mueller J et al Maximizing overall diversity for improved uncertainty estimates in deep ensembles, 2020.

[19] Li S , Tan Y, Ke S et al Immunogenicity prediction with dual attention enables vaccine target selection, 2025.

[20] Guo C , Pleiss G, Sun Y et al On calibration of modern neural networks, 2017.

[21] Gordon Wilson A , Izmailov P. Bayesian deep learning and a probabilistic perspective of generalization, 2022.

[22] Ovadia Y , Fertig E, Ren J et al Can you trust your model’s uncertainty? evaluating predictive uncertainty under dataset shift, 2019.

[23] Kristiadi A , Hein M, Hennig P. Being bayesian, even just a bit, fixes overconfidence in relu networks, 2020.

[24] Goan E , Fookes C. Bayesian neural networks: an introduction and survey. Springer International Publishing, 2020, 45–87.

[25] Ritter H , Botev A, Barber D. A scalable laplace approximation for neural networks. In *International Conference on Learning Representations*, 2018.

[26] MacKay DJC. A practical Bayesian framework for backpropagation networks. Neural Comput 1992;4:448–72.

[27] Lee J , Humt M, Feng J et al Estimating model uncertainty of neural networks in sparse information form, 2020.

[28] Salakhutdinov R , Mnih A. Bayesian probabilistic matrix factorization using Markov chain Monte Carlo. In *Proceedings of the 25th International Conference on Machine Learning*, ICML ’08, p.880–887. New York, NY: Association for Computing Machinery, 2008.

[29] Zhang J , Kailkhura B, Yong T et al Mix-n-match: ensemble and compositional methods for uncertainty calibration in deep learning, 2020.

[30] Lakshminarayanan B , Pritzel A, Blundell C. Simple and scalable predictive uncertainty estimation using deep ensembles, 2017.

[31] Bao W , Yu Q, Kong Y. Evidential deep learning for open set action recognition, 2021.

[32] Nikolas Angelopoulos A , Bates S, Jordan M et al Uncertainty sets for image classifiers using conformal prediction. In *International Conference on Learning Representations*, 2021.

[33] Harrison J , Willes J, Snoek J. Variational Bayesian last layers, 2024.

[34] Wilson AG , Hu Z, Salakhutdinov R et al Stochastic variational deep kernel learning, 2016.

[35] Maddox W , Garipov T, Izmailov P et al A simple baseline for Bayesian uncertainty in deep learning, 2019.

[36] Welling M , Teh YW. Bayesian learning via stochastic gradient langevin dynamics. In *Proceedings of the 28th International Conference on International Conference on Machine Learning*, ICML’11, p.681–688, Madison, WI: Omnipress, 2011.

[37] Sensoy M , Kaplan L, Kandemir M. Evidential deep learning to quantify classification uncertainty, 2018.

[38] Schellekens H. Bioequivalence and the immunogenicity of biopharmaceuticals. Nat Rev Drug Discov 2002;1:457–62. 12119747 10.1038/nrd818

[39] Calis JJA , Maybeno M, Greenbaum JA et al Properties of mhc class i presented peptides that enhance immunogenicity. PLoS Comput Biol 2013;9:e1003266. 24204222 10.1371/journal.pcbi.1003266 PMC3808449

[40] Lee CH , Huh J, Buckley PR et al A robust deep learning workflow to predict CD8 + t-cell epitopes. Genome Med 2023;15:70. 37705109 10.1186/s13073-023-01225-z PMC10498576

[41] Rives A , Meier J, Sercu T et al Biological structure and function emerge from scaling unsupervised learning to 250 million protein sequences. Proc Natl Acad Sci USA 2021;118:e2016239118. 33876751 10.1073/pnas.2016239118 PMC8053943

[42] Heinzinger M , Elnaggar A, Wang Y et al Modeling aspects of the language of life through transfer-learning protein sequences. BMC Bioinf 2019;20:723. 10.1186/s12859-019-3220-8 PMC6918593 31847804

[43] Vig J , Madani A, Varshney LR et al Bertology meets biology: interpreting attention in protein language models, 2021.

[44] Rao R , Meier J, Sercu T et al Transformer protein language models are unsupervised structure learners. In *International Conference on Learning Representations*, 2021.

[45] Heinzinger M , Weissenow K, Sanchez JG et al Bilingual language model for protein sequence and structure. NAR Genom Bioinform 2024;6:lqae150.11 39633723 10.1093/nargab/lqae150 PMC11616678

[46] Elnaggar A , Essam H, Salah-Eldin W et al Ankh: optimized protein language model unlocks general-purpose modelling, 2023.

[47] Lin Z , Akin H, Rao R et al Evolutionary-scale prediction of atomic-level protein structure with a language model. Science 2023;379:1123–30. 36927031 10.1126/science.ade2574

[48] Elnaggar A , Heinzinger M, Dallago C et al ProtTrans: toward understanding the language of life through self-supervised learning. IEEE Trans Pattern Anal Mach Intell 2022;44:7112–27. 34232869 10.1109/TPAMI.2021.3095381

[49] Murphy KP , Probabilistic Machine Learning: An Introduction. MIT Press, 2022.

[50] Brier GW. Verification of forecasts expressed in terms of probability. Monthly Weather Rev 1950;78:1–3.

[51] van Kempen M , Kim SS, Tumescheit C et al Fast and accurate protein structure search with foldseek. Nat Biotechnol 2024;42:243–6. February 37156916 10.1038/s41587-023-01773-0 PMC10869269

[52] Hayes T , Rao R, Akin H et al Simulating 500 million years of evolution with a language model. Science 2025;387:850–8. 39818825 10.1126/science.ads0018

[53] O’Brien H , Salm M, Morton LT et al A modular protein language modelling approach to immunogenicity prediction. PLoS Comput Biol 2024;20:e1012511. 39527593 10.1371/journal.pcbi.1012511 PMC11581412

[54] Wu J , Wang W, Zhang J et al DeepHLApan: a deep learning approach for neoantigen prediction considering both HLA-peptide binding and immunogenicity. Front Immunol 2019;10:2559. 31736974 10.3389/fimmu.2019.02559 PMC6838785

[55] Yu C , Fang X, Liu H. A unified cross-attention model for predicting antigen binding specificity to both hla and tcr molecules, 2025.

[56] Izmailov P , Maddox WJ, Kirichenko P et al Subspace inference for bayesian deep learning, 2019.

[57] Fan X , Zhang S, Tanwisuth K et al Contextual dropout: an efficient sample-dependent dropout module, 2021.

[58] Yang AX , Robeyns M, Wang X et al Bayesian low-rank adaptation for large language models, 2024.

[59] Wang Y , Shi H, Han L et al Blob: Bayesian low-rank adaptation by backpropagation for large language models, 2025.

[60] Rahmati AH , Jantre S, Zhang W et al C-lora: contextual low-rank adaptation for uncertainty estimation in large language models, 2025.

[61] Tan Y , Li M, Zhou Z et al PETA: evaluating the impact of protein transfer learning with sub-word tokenization on downstream applications. J Cheminform 2024;16:92. 39095917 10.1186/s13321-024-00884-3 PMC11297785

[62] Greenman KP , Amini AP, Yang KK. Benchmarking uncertainty quantification for protein engineering. PLoS Comput Biol 2025;21:e1012639. January 39775201 10.1371/journal.pcbi.1012639 PMC11741572

[63] Zai X , Zhao Y, Wang X et al Integrating protein language and geometric deep learning models for enhanced vaccine antigen prediction. Nat Commun 2025;17:1033. 41423641 10.1038/s41467-025-67778-2 PMC12847940

[64] Prabakaran R , Bromberg Y. Quantifying uncertainty in protein representations across models and task. *bioRxiv*, 2025. 10.1038/s41592-026-03028-7 PMC13076229 41922570

