# Supplementary material for: ImmUQBench: a benchmark on uncertainty quantification of protein immunogenicity prediction
Source: Oxf Open Immunol. 2026 Mar 3;7(1):iqag003. doi: 10.1093/oxfimm/iqag003 (PMC12996882; doi:10.1093/oxfimm/iqag003)
Supplement: iqag003_Supplementary_Data [file iqag003_supplementary_data.zip › 10-Mar-2026_061035_ImmUQBench-supplementary.pdf]

Supplementary Materials

Alif Bin Abdul Qayyum, Amir Hossein Rahmati, Xiaoning Qian
and Byung-Jun Yoon

1 Additional ID (In-Distribution) Results

Table 1, 2 and 3 show the evaluation results for in-distribution scenario for models with different protein language model.

2 Additional OOD (Out-of-Distribution) Results

2.1 Evaluation of Models Trained on Immuno-Virus Dataset

Table 4 and 5 show the evaluation of models trained with Immuno-Virus dataset on Immuno-Bacteria and Immuno-Tumor datasets accordingly.

2.2 Evaluation of Models Trained on Immuno-Bacteria Dataset

Table 6 and 7 show the evaluation of models trained with Immuno-Bacteria dataset on Immuno-Virus and Immuno-Tumor datasets accordingly.

2.3 Evaluation of Models Trained on Immuno-Tumor Dataset

Table 8 and 9 show the evaluation of models trained with Immuno-Bacteria dataset on Immuno-Virus and Immuno-Tumor datasets accordingly.

3 Additional Experiments with Hyper-parameters

For DVBLL, LA and SVDKL, we modified the last MLP segment of the original VenusVaccine architecture by adding an extra linear layer and converted this extra linear layer as the probabilistic segment. We experimented with three different dimensions for this extra linear layer: 32, 64, 128. Table 10, 11 and 12 shows the results Immuno-Virus, Immuno-Bacteria and Immuno-Tumor datasets accordingly.

Table 1: In-Distribution immunogenicity prediction evaluation results for Immuno-Virus dataset.

PLM	Model	Accuracy	Precision	Recall	F1 Score	AUC ROC	ECE	NLL	Brier Score
ESMC	Deterministic	0.8975$_{\pm 0.0134}$	0.8859$_{\pm 0.0122}$	0.9161$_{\pm 0.0236}$	0.9006$_{\pm 0.0138}$	0.9638$_{\pm 0.0046}$	0.0344$_{\pm 0.0085}$	0.2559$_{\pm 0.0225}$	0.0746$_{\pm 0.0062}$
	TS	0.8975$_{\pm 0.0134}$	0.8859$_{\pm 0.0122}$	0.9161$_{\pm 0.0236}$	0.9006$_{\pm 0.0138}$	0.9638$_{\pm 0.0046}$	0.0354$_{\pm 0.0087}$	0.2525$_{\pm 0.0209}$	0.0743$_{\pm 0.0061}$
	LA	0.9025$_{\pm 0.0037}$	0.8863$_{\pm 0.0080}$	0.9270$_{\pm 0.0125}$	0.9061$_{\pm 0.0040}$	0.9641$_{\pm 0.0028}$	0.0301$_{\pm 0.0071}$	0.2486$_{\pm 0.0079}$	0.0725$_{\pm 0.0025}$
	DVBLL	0.8990$_{\pm 0.0118}$	0.9004$_{\pm 0.0117}$	0.9007$_{\pm 0.0206}$	0.9004$_{\pm 0.0121}$	0.9613$_{\pm 0.0068}$	0.0514$_{\pm 0.0151}$	0.2915$_{\pm 0.0460}$	0.0775$_{\pm 0.0088}$
	EDL	0.8748$_{\pm 0.0401}$	0.8952$_{\pm 0.0231}$	0.8571$_{\pm 0.1086}$	0.8703$_{\pm 0.0551}$	0.9504$_{\pm 0.0211}$	0.1385$_{\pm 0.0606}$	0.3825$_{\pm 0.1314}$	0.1154$_{\pm 0.0543}$
	SWAG	0.9018$_{\pm 0.0069}$	0.8879$_{\pm 0.0434}$	0.9285$_{\pm 0.0429}$	0.9057$_{\pm 0.0028}$	0.9673$_{\pm 0.0039}$	0.1313$_{\pm 0.0227}$	0.3189$_{\pm 0.0260}$	0.0914$_{\pm 0.0098}$
	MCD	0.8985$_{\pm 0.0130}$	0.8869$_{\pm 0.0117}$	0.9171$_{\pm 0.0224}$	0.9016$_{\pm 0.0133}$	0.9637$_{\pm 0.0046}$	0.0344$_{\pm 0.0094}$	0.2549$_{\pm 0.0224}$	0.0745$_{\pm 0.0062}$
	DKL	0.8829$_{\pm 0.0142}$	0.8691$_{\pm 0.0128}$	0.9057$_{\pm 0.0193}$	0.8869$_{\pm 0.0142}$	0.9402$_{\pm 0.0178}$	0.0398$_{\pm 0.0116}$	0.3039$_{\pm 0.0361}$	0.0892$_{\pm 0.0113}$
	SGLD	0.8781$_{\pm 0.0046}$	0.8683$_{\pm 0.0076}$	0.8958$_{\pm 0.0075}$	0.8818$_{\pm 0.0043}$	0.9520$_{\pm 0.0027}$	0.0341$_{\pm 0.0062}$	0.2855$_{\pm 0.0105}$	0.0852$_{\pm 0.0017}$
ProstT5	Deterministic	0.8872$_{\pm 0.0080}$	0.9008$_{\pm 0.0178}$	0.8744$_{\pm 0.0090}$	0.8873$_{\pm 0.0070}$	0.9557$_{\pm 0.0042}$	0.0457$_{\pm 0.0170}$	0.3066$_{\pm 0.0329}$	0.0852$_{\pm 0.0047}$
	TS	0.8872$_{\pm 0.0080}$	0.9008$_{\pm 0.0178}$	0.8744$_{\pm 0.0090}$	0.8873$_{\pm 0.0070}$	0.9557$_{\pm 0.0042}$	0.0420$_{\pm 0.0123}$	0.2923$_{\pm 0.0197}$	0.0842$_{\pm 0.0044}$
	LA	0.8846$_{\pm 0.0122}$	0.8855$_{\pm 0.0061}$	0.8873$_{\pm 0.0229}$	0.8863$_{\pm 0.0133}$	0.9540$_{\pm 0.0108}$	0.0382$_{\pm 0.0103}$	0.2877$_{\pm 0.0476}$	0.0845$_{\pm 0.0105}$
	DVBLL	0.8889$_{\pm 0.0183}$	0.8872$_{\pm 0.0073}$	0.8948$_{\pm 0.0352}$	0.8907$_{\pm 0.0199}$	0.9520$_{\pm 0.0103}$	0.0550$_{\pm 0.0118}$	0.3207$_{\pm 0.0375}$	0.0876$_{\pm 0.0105}$
	EDL	0.9015$_{\pm 0.0040}$	0.9029$_{\pm 0.0154}$	0.9037$_{\pm 0.0185}$	0.9030$_{\pm 0.0043}$	0.9629$_{\pm 0.0010}$	0.0657$_{\pm 0.0180}$	0.2771$_{\pm 0.0127}$	0.0772$_{\pm 0.0035}$
	SWAG	0.9091$_{\pm 0.0051}$	0.9311$_{\pm 0.0122}$	0.8868$_{\pm 0.0194}$	0.9082$_{\pm 0.0062}$	0.9604$_{\pm 0.0019}$	0.0993$_{\pm 0.0206}$	0.3054$_{\pm 0.0288}$	0.0852$_{\pm 0.0102}$
	MCD	0.8869$_{\pm 0.0081}$	0.9012$_{\pm 0.0176}$	0.8734$_{\pm 0.0103}$	0.8869$_{\pm 0.0072}$	0.9555$_{\pm 0.0041}$	0.0454$_{\pm 0.0171}$	0.3017$_{\pm 0.0291}$	0.0849$_{\pm 0.0047}$
	DKL	0.8456$_{\pm 0.0341}$	0.8213$_{\pm 0.0445}$	0.8928$_{\pm 0.0218}$	0.8550$_{\pm 0.0288}$	0.8944$_{\pm 0.0613}$	0.0820$_{\pm 0.0492}$	0.3984$_{\pm 0.0862}$	0.1232$_{\pm 0.0325}$
	SGLD	0.8816$_{\pm 0.0066}$	0.8760$_{\pm 0.0064}$	0.8933$_{\pm 0.0114}$	0.8845$_{\pm 0.0067}$	0.9565$_{\pm 0.0032}$	0.0362$_{\pm 0.0054}$	0.2737$_{\pm 0.0099}$	0.0836$_{\pm 0.0030}$
Ankh	Deterministic	0.8914$_{\pm 0.0084}$	0.9003$_{\pm 0.0146}$	0.8844$_{\pm 0.0161}$	0.8921$_{\pm 0.0084}$	0.9571$_{\pm 0.0048}$	0.0442$_{\pm 0.0241}$	0.3046$_{\pm 0.0557}$	0.0844$_{\pm 0.0084}$
	TS	0.8914$_{\pm 0.0084}$	0.9003$_{\pm 0.0146}$	0.8844$_{\pm 0.0161}$	0.8921$_{\pm 0.0084}$	0.9571$_{\pm 0.0048}$	0.0390$_{\pm 0.0202}$	0.2889$_{\pm 0.0383}$	0.0833$_{\pm 0.0072}$
	LA	0.8804$_{\pm 0.0180}$	0.8763$_{\pm 0.0350}$	0.8923$_{\pm 0.0130}$	0.8837$_{\pm 0.0144}$	0.9541$_{\pm 0.0063}$	0.0370$_{\pm 0.0106}$	0.2790$_{\pm 0.0217}$	0.0855$_{\pm 0.0092}$
	DVBLL	0.7426$_{\pm 0.1883}$	0.7438$_{\pm 0.1928}$	0.9355$_{\pm 0.0571}$	0.8079$_{\pm 0.1102}$	0.7802$_{\pm 0.2100}$	0.0450$_{\pm 0.0282}$	0.4686$_{\pm 0.1857}$	0.1504$_{\pm 0.0811}$
	EDL	0.9020$_{\pm 0.0042}$	0.8957$_{\pm 0.0087}$	0.9136$_{\pm 0.0185}$	0.9044$_{\pm 0.0053}$	0.9653$_{\pm 0.0014}$	0.1063$_{\pm 0.0077}$	0.3024$_{\pm 0.0112}$	0.0824$_{\pm 0.0037}$
	SWAG	0.8821$_{\pm 0.0519}$	0.8741$_{\pm 0.0881}$	0.9166$_{\pm 0.0507}$	0.8899$_{\pm 0.0382}$	0.9638$_{\pm 0.0060}$	0.1577$_{\pm 0.0280}$	0.3600$_{\pm 0.0432}$	0.1078$_{\pm 0.0204}$
	MCD	0.8912$_{\pm 0.0077}$	0.8998$_{\pm 0.0132}$	0.8844$_{\pm 0.0161}$	0.8919$_{\pm 0.0079}$	0.9570$_{\pm 0.0048}$	0.0449$_{\pm 0.0219}$	0.2995$_{\pm 0.0495}$	0.0842$_{\pm 0.0081}$
	DKL	0.8446$_{\pm 0.0693}$	0.8380$_{\pm 0.0918}$	0.8794$_{\pm 0.0082}$	0.8552$_{\pm 0.0511}$	0.8882$_{\pm 0.1303}$	0.0839$_{\pm 0.0851}$	0.3670$_{\pm 0.1566}$	0.1174$_{\pm 0.0608}$
	SGLD	0.8947$_{\pm 0.0067}$	0.9010$_{\pm 0.0146}$	0.8908$_{\pm 0.0080}$	0.8957$_{\pm 0.0059}$	0.9633$_{\pm 0.0021}$	0.0442$_{\pm 0.0128}$	0.2517$_{\pm 0.0056}$	0.0765$_{\pm 0.0018}$
ESM2	Deterministic	0.8877$_{\pm 0.0100}$	0.8867$_{\pm 0.0153}$	0.8933$_{\pm 0.0213}$	0.8897$_{\pm 0.0103}$	0.9603$_{\pm 0.0034}$	0.0313$_{\pm 0.0084}$	0.2616$_{\pm 0.0135}$	0.0796$_{\pm 0.0047}$
	TS	0.8877$_{\pm 0.0100}$	0.8867$_{\pm 0.0153}$	0.8933$_{\pm 0.0213}$	0.8897$_{\pm 0.0103}$	0.9603$_{\pm 0.0034}$	0.0293$_{\pm 0.0077}$	0.2599$_{\pm 0.0130}$	0.0794$_{\pm 0.0047}$
	LA	0.8806$_{\pm 0.0102}$	0.8575$_{\pm 0.0235}$	0.9186$_{\pm 0.0191}$	0.8866$_{\pm 0.0084}$	0.9594$_{\pm 0.0031}$	0.0578$_{\pm 0.0175}$	0.2951$_{\pm 0.0276}$	0.0852$_{\pm 0.0039}$
	DVBLL	0.8577$_{\pm 0.0448}$	0.8319$_{\pm 0.0692}$	0.9146$_{\pm 0.0281}$	0.8688$_{\pm 0.0326}$	0.9350$_{\pm 0.0390}$	0.0656$_{\pm 0.0412}$	0.3474$_{\pm 0.1118}$	0.1100$_{\pm 0.0419}$
	EDL	0.8033$_{\pm 0.1479}$	0.7915$_{\pm 0.1426}$	0.9221$_{\pm 0.0419}$	0.8401$_{\pm 0.0835}$	0.9501$_{\pm 0.0136}$	0.1539$_{\pm 0.0424}$	0.3794$_{\pm 0.0571}$	0.1125$_{\pm 0.0263}$
	SWAG	0.9000$_{\pm 0.0110}$	0.8839$_{\pm 0.0488}$	0.9310$_{\pm 0.0430}$	0.9045$_{\pm 0.0061}$	0.9693$_{\pm 0.0030}$	0.1439$_{\pm 0.0146}$	0.3304$_{\pm 0.0167}$	0.0962$_{\pm 0.0093}$
	MCD	0.8884$_{\pm 0.0095}$	0.8876$_{\pm 0.0153}$	0.8938$_{\pm 0.0218}$	0.8904$_{\pm 0.0099}$	0.9604$_{\pm 0.0034}$	0.0316$_{\pm 0.0091}$	0.2612$_{\pm 0.0131}$	0.0796$_{\pm 0.0047}$
	DKL	0.8950$_{\pm 0.0171}$	0.8866$_{\pm 0.0367}$	0.9122$_{\pm 0.0152}$	0.8985$_{\pm 0.0135}$	0.9405$_{\pm 0.0341}$	0.0445$_{\pm 0.0285}$	0.2937$_{\pm 0.0698}$	0.0853$_{\pm 0.0208}$
	SGLD	0.8738$_{\pm 0.0039}$	0.8683$_{\pm 0.0085}$	0.8859$_{\pm 0.0068}$	0.8769$_{\pm 0.0034}$	0.9560$_{\pm 0.0008}$	0.0559$_{\pm 0.0084}$	0.2829$_{\pm 0.0046}$	0.0854$_{\pm 0.0013}$
Prot Bert	Deterministic	0.8935$_{\pm 0.0091}$	0.8766$_{\pm 0.0225}$	0.9206$_{\pm 0.0112}$	0.8978$_{\pm 0.0069}$	0.9572$_{\pm 0.0021}$	0.0513$_{\pm 0.0027}$	0.2886$_{\pm 0.0125}$	0.0821$_{\pm 0.0010}$
	TS	0.8935$_{\pm 0.0091}$	0.8766$_{\pm 0.0225}$	0.9206$_{\pm 0.0112}$	0.8978$_{\pm 0.0069}$	0.9572$_{\pm 0.0021}$	0.0497$_{\pm 0.0049}$	0.2819$_{\pm 0.0096}$	0.0822$_{\pm 0.0015}$
	LA	0.8887$_{\pm 0.0086}$	0.8731$_{\pm 0.0115}$	0.9136$_{\pm 0.0138}$	0.8928$_{\pm 0.0084}$	0.9581$_{\pm 0.0053}$	0.0451$_{\pm 0.0148}$	0.2909$_{\pm 0.0233}$	0.0823$_{\pm 0.0063}$
	DVBLL	0.8932$_{\pm 0.0115}$	0.9028$_{\pm 0.0135}$	0.8854$_{\pm 0.0306}$	0.8936$_{\pm 0.0130}$	0.9582$_{\pm 0.0022}$	0.0392$_{\pm 0.0134}$	0.2873$_{\pm 0.0203}$	0.0792$_{\pm 0.0048}$
	EDL	0.9005$_{\pm 0.0046}$	0.8953$_{\pm 0.0109}$	0.9107$_{\pm 0.0092}$	0.9028$_{\pm 0.0041}$	0.9614$_{\pm 0.0028}$	0.1031$_{\pm 0.0189}$	0.3043$_{\pm 0.0222}$	0.0845$_{\pm 0.0065}$
	SWAG	0.9038$_{\pm 0.0116}$	0.9037$_{\pm 0.0397}$	0.9112$_{\pm 0.0364}$	0.9059$_{\pm 0.0091}$	0.9643$_{\pm 0.0038}$	0.1157$_{\pm 0.0326}$	0.3129$_{\pm 0.0312}$	0.0911$_{\pm 0.0106}$
	MCD	0.8912$_{\pm 0.0091}$	0.8744$_{\pm 0.0233}$	0.9186$_{\pm 0.0135}$	0.8956$_{\pm 0.0068}$	0.9572$_{\pm 0.0021}$	0.0492$_{\pm 0.0030}$	0.2868$_{\pm 0.0113}$	0.0821$_{\pm 0.0010}$
	DKL	0.8685$_{\pm 0.0372}$	0.8535$_{\pm 0.0409}$	0.8958$_{\pm 0.0286}$	0.8740$_{\pm 0.0341}$	0.9171$_{\pm 0.0610}$	0.0926$_{\pm 0.0655}$	0.3644$_{\pm 0.1261}$	0.1096$_{\pm 0.0408}$
	SGLD	0.8907$_{\pm 0.0039}$	0.8987$_{\pm 0.0073}$	0.8844$_{\pm 0.0064}$	0.8914$_{\pm 0.0038}$	0.9572$_{\pm 0.0012}$	0.0487$_{\pm 0.0042}$	0.2750$_{\pm 0.0028}$	0.0819$_{\pm 0.0009}$
-	Ensemble	0.9199$_{\pm 0.0049}$	0.9203$_{\pm 0.0044}$	0.9221$_{\pm 0.0126}$	0.9211$_{\pm 0.0055}$	0.9761$_{\pm 0.0018}$	0.0451$_{\pm 0.0120}$	0.2121$_{\pm 0.0102}$	0.0616$_{\pm 0.0028}$

Table 2: In-Distribution immunogenicity prediction evaluation results for Immuno-Bacteria dataset.

PLM	Model	Accuracy	Precision	Recall	F1 Score	AUC ROC	ECE	NLL	Brier Score
ESMC	Deterministic	0.7923$_{\pm 0.0141}$	0.7267$_{\pm 0.0478}$	0.6701$_{\pm 0.0663}$	0.6928$_{\pm 0.0183}$	0.8564$_{\pm 0.0089}$	0.0957$_{\pm 0.0358}$	0.4961$_{\pm 0.0485}$	0.1503$_{\pm 0.0124}$
	TS	0.7923$_{\pm 0.0141}$	0.7267$_{\pm 0.0478}$	0.6701$_{\pm 0.0663}$	0.6928$_{\pm 0.0183}$	0.8564$_{\pm 0.0089}$	0.0862$_{\pm 0.0385}$	0.4784$_{\pm 0.0382}$	0.1484$_{\pm 0.0130}$
	LA	0.8149$_{\pm 0.0106}$	0.7722$_{\pm 0.0253}$	0.6724$_{\pm 0.0385}$	0.7178$_{\pm 0.0200}$	0.8655$_{\pm 0.0069}$	0.1049$_{\pm 0.0251}$	0.5780$_{\pm 0.1031}$	0.1487$_{\pm 0.0073}$
	DVBLL	0.7992$_{\pm 0.0243}$	0.7194$_{\pm 0.0539}$	0.7149$_{\pm 0.0526}$	0.7142$_{\pm 0.0273}$	0.8486$_{\pm 0.0178}$	0.1105$_{\pm 0.0483}$	0.5663$_{\pm 0.1132}$	0.1587$_{\pm 0.0148}$
	EDL	0.8149$_{\pm 0.0081}$	0.7560$_{\pm 0.0367}$	0.7057$_{\pm 0.0639}$	0.7267$_{\pm 0.0217}$	0.8720$_{\pm 0.0087}$	0.0655$_{\pm 0.0140}$	0.4380$_{\pm 0.0145}$	0.1376$_{\pm 0.0055}$
	SWAG	0.8262$_{\pm 0.0089}$	0.7843$_{\pm 0.0566}$	0.7126$_{\pm 0.0744}$	0.7410$_{\pm 0.0145}$	0.8816$_{\pm 0.0048}$	0.1083$_{\pm 0.0325}$	0.4950$_{\pm 0.0613}$	0.1376$_{\pm 0.0111}$
	MCD	0.7927$_{\pm 0.0140}$	0.7263$_{\pm 0.0474}$	0.6724$_{\pm 0.0671}$	0.6939$_{\pm 0.0189}$	0.8567$_{\pm 0.0087}$	0.0956$_{\pm 0.0359}$	0.4932$_{\pm 0.0461}$	0.1502$_{\pm 0.0124}$
	DKL	0.8048$_{\pm 0.0053}$	0.7323$_{\pm 0.0283}$	0.7046$_{\pm 0.0433}$	0.7165$_{\pm 0.0126}$	0.8399$_{\pm 0.0100}$	0.0862$_{\pm 0.0187}$	0.5011$_{\pm 0.0296}$	0.1546$_{\pm 0.0056}$
	SGLD	0.7952$_{\pm 0.0075}$	0.7101$_{\pm 0.0076}$	0.7034$_{\pm 0.0352}$	0.7063$_{\pm 0.0174}$	0.8511$_{\pm 0.0054}$	0.1256$_{\pm 0.0179}$	0.6303$_{\pm 0.0585}$	0.1623$_{\pm 0.0040}$
ProstT5	Deterministic	0.7883$_{\pm 0.0114}$	0.7211$_{\pm 0.0264}$	0.6506$_{\pm 0.0452}$	0.6825$_{\pm 0.0234}$	0.8387$_{\pm 0.0140}$	0.1101$_{\pm 0.0338}$	0.6113$_{\pm 0.1188}$	0.1642$_{\pm 0.0113}$
	TS	0.7883$_{\pm 0.0114}$	0.7211$_{\pm 0.0264}$	0.6506$_{\pm 0.0452}$	0.6825$_{\pm 0.0234}$	0.8387$_{\pm 0.0140}$	0.0966$_{\pm 0.0325}$	0.5545$_{\pm 0.0785}$	0.1601$_{\pm 0.0103}$
	LA	0.7742$_{\pm 0.0244}$	0.6861$_{\pm 0.0435}$	0.6690$_{\pm 0.0904}$	0.6727$_{\pm 0.0471}$	0.8292$_{\pm 0.0219}$	0.0954$_{\pm 0.0312}$	0.5275$_{\pm 0.0489}$	0.1656$_{\pm 0.0141}$
	DVBLL	0.7859$_{\pm 0.0109}$	0.7202$_{\pm 0.0419}$	0.6552$_{\pm 0.1070}$	0.6774$_{\pm 0.0510}$	0.8288$_{\pm 0.0312}$	0.1033$_{\pm 0.0553}$	0.6550$_{\pm 0.1951}$	0.1685$_{\pm 0.0158}$
	EDL	0.7234$_{\pm 0.1864}$	0.6783$_{\pm 0.1645}$	0.7598$_{\pm 0.1284}$	0.6855$_{\pm 0.0852}$	0.8446$_{\pm 0.0259}$	0.0977$_{\pm 0.0732}$	0.4917$_{\pm 0.0786}$	0.1597$_{\pm 0.0348}$
	SWAG	0.8214$_{\pm 0.0063}$	0.7737$_{\pm 0.0207}$	0.6966$_{\pm 0.0480}$	0.7315$_{\pm 0.0195}$	0.8578$_{\pm 0.0062}$	0.0966$_{\pm 0.0393}$	0.5153$_{\pm 0.0792}$	0.1460$_{\pm 0.0074}$
	MCD	0.7887$_{\pm 0.0110}$	0.7217$_{\pm 0.0280}$	0.6517$_{\pm 0.0459}$	0.6833$_{\pm 0.0227}$	0.8389$_{\pm 0.0140}$	0.1086$_{\pm 0.0326}$	0.5968$_{\pm 0.1070}$	0.1636$_{\pm 0.0112}$
	DKL	0.7750$_{\pm 0.0112}$	0.7147$_{\pm 0.0183}$	0.5989$_{\pm 0.0556}$	0.6499$_{\pm 0.0313}$	0.7993$_{\pm 0.0430}$	0.0553$_{\pm 0.0102}$	0.5074$_{\pm 0.0235}$	0.1653$_{\pm 0.0093}$
	SGLD	0.8004$_{\pm 0.0036}$	0.7341$_{\pm 0.0066}$	0.6759$_{\pm 0.0059}$	0.7038$_{\pm 0.0051}$	0.8465$_{\pm 0.0031}$	0.0443$_{\pm 0.0091}$	0.4608$_{\pm 0.0036}$	0.1473$_{\pm 0.0006}$
Ankh	Deterministic	0.8069$_{\pm 0.0124}$	0.7444$_{\pm 0.0495}$	0.6966$_{\pm 0.0536}$	0.7162$_{\pm 0.0173}$	0.8573$_{\pm 0.0099}$	0.1102$_{\pm 0.0109}$	0.5823$_{\pm 0.0598}$	0.1521$_{\pm 0.0028}$
	TS	0.8069$_{\pm 0.0124}$	0.7444$_{\pm 0.0495}$	0.6966$_{\pm 0.0536}$	0.7162$_{\pm 0.0173}$	0.8573$_{\pm 0.0099}$	0.0954$_{\pm 0.0127}$	0.5268$_{\pm 0.0371}$	0.1483$_{\pm 0.0029}$
	LA	0.7819$_{\pm 0.0105}$	0.7182$_{\pm 0.0409}$	0.6379$_{\pm 0.0898}$	0.6690$_{\pm 0.0412}$	0.8504$_{\pm 0.0018}$	0.0976$_{\pm 0.0337}$	0.5599$_{\pm 0.1258}$	0.1565$_{\pm 0.0063}$
	DVBLL	0.7073$_{\pm 0.0688}$	0.4427$_{\pm 0.3630}$	0.2816$_{\pm 0.3359}$	0.2887$_{\pm 0.3358}$	0.6239$_{\pm 0.1697}$	0.0694$_{\pm 0.0334}$	0.6513$_{\pm 0.1136}$	0.2024$_{\pm 0.0378}$
	EDL	0.7746$_{\pm 0.0244}$	0.7619$_{\pm 0.0364}$	0.5356$_{\pm 0.1522}$	0.6129$_{\pm 0.0888}$	0.8379$_{\pm 0.0162}$	0.1202$_{\pm 0.0361}$	0.5189$_{\pm 0.0452}$	0.1696$_{\pm 0.0193}$
	SWAG	0.8198$_{\pm 0.0181}$	0.7488$_{\pm 0.0524}$	0.7460$_{\pm 0.0469}$	0.7442$_{\pm 0.0123}$	0.8662$_{\pm 0.0060}$	0.1250$_{\pm 0.0460}$	0.4980$_{\pm 0.0402}$	0.1488$_{\pm 0.0188}$
	MCD	0.8069$_{\pm 0.0124}$	0.7444$_{\pm 0.0495}$	0.6966$_{\pm 0.0536}$	0.7162$_{\pm 0.0173}$	0.8575$_{\pm 0.0100}$	0.1085$_{\pm 0.0113}$	0.5725$_{\pm 0.0605}$	0.1515$_{\pm 0.0029}$
	DKL	0.7899$_{\pm 0.0260}$	0.7051$_{\pm 0.0485}$	0.6966$_{\pm 0.0194}$	0.7001$_{\pm 0.0291}$	0.8351$_{\pm 0.0182}$	0.1098$_{\pm 0.0136}$	0.5370$_{\pm 0.0317}$	0.1592$_{\pm 0.0117}$
	SGLD	0.8069$_{\pm 0.0087}$	0.7428$_{\pm 0.0109}$	0.6874$_{\pm 0.0234}$	0.7139$_{\pm 0.0159}$	0.8504$_{\pm 0.0016}$	0.0433$_{\pm 0.0085}$	0.4452$_{\pm 0.0020}$	0.1406$_{\pm 0.0015}$
ESM2	Deterministic	0.8016$_{\pm 0.0133}$	0.7443$_{\pm 0.0232}$	0.6644$_{\pm 0.0535}$	0.7005$_{\pm 0.0288}$	0.8722$_{\pm 0.0056}$	0.0696$_{\pm 0.0228}$	0.4501$_{\pm 0.0352}$	0.1403$_{\pm 0.0095}$
	TS	0.8016$_{\pm 0.0133}$	0.7443$_{\pm 0.0232}$	0.6644$_{\pm 0.0535}$	0.7005$_{\pm 0.0288}$	0.8722$_{\pm 0.0056}$	0.0569$_{\pm 0.0224}$	0.4375$_{\pm 0.0250}$	0.1383$_{\pm 0.0081}$
	LA	0.8028$_{\pm 0.0190}$	0.7285$_{\pm 0.0473}$	0.7103$_{\pm 0.0663}$	0.7157$_{\pm 0.0283}$	0.8630$_{\pm 0.0109}$	0.0994$_{\pm 0.0382}$	0.5091$_{\pm 0.0832}$	0.1499$_{\pm 0.0169}$
	DVBLL	0.8129$_{\pm 0.0083}$	0.7522$_{\pm 0.0335}$	0.7023$_{\pm 0.0450}$	0.7244$_{\pm 0.0135}$	0.8492$_{\pm 0.0260}$	0.0963$_{\pm 0.0219}$	0.5986$_{\pm 0.0949}$	0.1480$_{\pm 0.0082}$
	EDL	0.6290$_{\pm 0.2272}$	0.5662$_{\pm 0.1763}$	0.8793$_{\pm 0.1010}$	0.6585$_{\pm 0.1137}$	0.7226$_{\pm 0.1820}$	0.1083$_{\pm 0.0341}$	0.5436$_{\pm 0.1234}$	0.1840$_{\pm 0.0542}$
	SWAG	0.8258$_{\pm 0.0091}$	0.7612$_{\pm 0.0343}$	0.7391$_{\pm 0.0374}$	0.7484$_{\pm 0.0109}$	0.8797$_{\pm 0.0064}$	0.0886$_{\pm 0.0280}$	0.4705$_{\pm 0.0617}$	0.1358$_{\pm 0.0078}$
	MCD	0.8020$_{\pm 0.0131}$	0.7453$_{\pm 0.0236}$	0.6644$_{\pm 0.0535}$	0.7009$_{\pm 0.0285}$	0.8721$_{\pm 0.0055}$	0.0668$_{\pm 0.0222}$	0.4484$_{\pm 0.0324}$	0.1402$_{\pm 0.0093}$
	DKL	0.8093$_{\pm 0.0103}$	0.7631$_{\pm 0.0135}$	0.6621$_{\pm 0.0347}$	0.7085$_{\pm 0.0208}$	0.8159$_{\pm 0.0450}$	0.1139$_{\pm 0.0505}$	0.5357$_{\pm 0.0542}$	0.1601$_{\pm 0.0204}$
	SGLD	0.8060$_{\pm 0.0073}$	0.7285$_{\pm 0.0086}$	0.7126$_{\pm 0.0182}$	0.7204$_{\pm 0.0123}$	0.8655$_{\pm 0.0015}$	0.0383$_{\pm 0.0024}$	0.4329$_{\pm 0.0016}$	0.1373$_{\pm 0.0007}$
Prot Bert	Deterministic	0.7940$_{\pm 0.0059}$	0.7164$_{\pm 0.0209}$	0.6885$_{\pm 0.0670}$	0.6994$_{\pm 0.0260}$	0.8582$_{\pm 0.0118}$	0.0876$_{\pm 0.0342}$	0.5059$_{\pm 0.0734}$	0.1510$_{\pm 0.0055}$
	TS	0.7940$_{\pm 0.0059}$	0.7164$_{\pm 0.0209}$	0.6885$_{\pm 0.0670}$	0.6994$_{\pm 0.0260}$	0.8582$_{\pm 0.0118}$	0.0806$_{\pm 0.0296}$	0.4807$_{\pm 0.0442}$	0.1485$_{\pm 0.0048}$
	LA	0.8028$_{\pm 0.0115}$	0.7405$_{\pm 0.0490}$	0.6897$_{\pm 0.0723}$	0.7093$_{\pm 0.0209}$	0.8531$_{\pm 0.0095}$	0.1002$_{\pm 0.0119}$	0.5550$_{\pm 0.0812}$	0.1509$_{\pm 0.0080}$
	DVBLL	0.8117$_{\pm 0.0091}$	0.7481$_{\pm 0.0255}$	0.7011$_{\pm 0.0262}$	0.7231$_{\pm 0.0114}$	0.8617$_{\pm 0.0114}$	0.0764$_{\pm 0.0159}$	0.5104$_{\pm 0.0439}$	0.1424$_{\pm 0.0034}$
	EDL	0.8000$_{\pm 0.0387}$	0.7736$_{\pm 0.0488}$	0.6287$_{\pm 0.1920}$	0.6693$_{\pm 0.1246}$	0.8673$_{\pm 0.0217}$	0.1016$_{\pm 0.0596}$	0.4599$_{\pm 0.0673}$	0.1472$_{\pm 0.0271}$
	SWAG	0.8206$_{\pm 0.0141}$	0.7502$_{\pm 0.0456}$	0.7414$_{\pm 0.0510}$	0.7432$_{\pm 0.0191}$	0.8715$_{\pm 0.0057}$	0.1127$_{\pm 0.0350}$	0.4697$_{\pm 0.0254}$	0.1447$_{\pm 0.0098}$
	MCD	0.7944$_{\pm 0.0056}$	0.7170$_{\pm 0.0229}$	0.6897$_{\pm 0.0666}$	0.7002$_{\pm 0.0252}$	0.8583$_{\pm 0.0117}$	0.0860$_{\pm 0.0330}$	0.5000$_{\pm 0.0661}$	0.1506$_{\pm 0.0056}$
	DKL	0.8048$_{\pm 0.0125}$	0.7350$_{\pm 0.0347}$	0.7011$_{\pm 0.0473}$	0.7156$_{\pm 0.0179}$	0.8436$_{\pm 0.0139}$	0.0723$_{\pm 0.0225}$	0.4797$_{\pm 0.0234}$	0.1488$_{\pm 0.0065}$
	SGLD	0.8149$_{\pm 0.0043}$	0.7541$_{\pm 0.0080}$	0.7011$_{\pm 0.0081}$	0.7266$_{\pm 0.0063}$	0.8701$_{\pm 0.0018}$	0.0357$_{\pm 0.0062}$	0.4213$_{\pm 0.0030}$	0.1327$_{\pm 0.0013}$
-	Ensemble	0.8210$_{\pm 0.0106}$	0.7669$_{\pm 0.0264}$	0.7057$_{\pm 0.0197}$	0.7345$_{\pm 0.0127}$	0.8791$_{\pm 0.0041}$	0.0503$_{\pm 0.0058}$	0.4210$_{\pm 0.0127}$	0.1304$_{\pm 0.0037}$

Table 3: In-Distribution immunogenicity prediction evaluation results for Immuno-Tumor dataset.

PLM	Model	Accuracy	Precision	Recall	F1 Score	AUC ROC	ECE	NLL	Brier Score
ESMC	Deterministic	$0.7321_{\pm 0.0273}$	$0.6474_{\pm 0.0220}$	$0.7049_{\pm 0.1722}$	$0.6618_{\pm 0.0855}$	$0.8337_{\pm 0.0157}$	$0.0753_{\pm 0.0263}$	$0.4930_{\pm 0.0225}$	$0.1675_{\pm 0.0090}$
	TS	$0.7321_{\pm 0.0273}$	$0.6474_{\pm 0.0220}$	$0.7049_{\pm 0.1722}$	$0.6618_{\pm 0.0855}$	$0.8337_{\pm 0.0157}$	$0.0781_{\pm 0.0124}$	$0.4919_{\pm 0.0217}$	$0.1674_{\pm 0.0090}$
	LA	$0.7474_{\pm 0.0205}$	$0.6722_{\pm 0.0378}$	$0.7115_{\pm 0.1397}$	$0.6814_{\pm 0.0567}$	$0.8465_{\pm 0.0227}$	$0.0979_{\pm 0.0213}$	$0.4886_{\pm 0.0374}$	$0.1632_{\pm 0.0128}$
	DVBLL	$0.7615_{\pm 0.0124}$	$0.6716_{\pm 0.0276}$	$0.7803_{\pm 0.1229}$	$0.7144_{\pm 0.0463}$	$0.8457_{\pm 0.0084}$	$0.1333_{\pm 0.0406}$	$0.5827_{\pm 0.1026}$	$0.1753_{\pm 0.0133}$
	EDL	$0.7654_{\pm 0.0155}$	$0.6902_{\pm 0.0286}$	$0.7311_{\pm 0.0304}$	$0.7091_{\pm 0.0141}$	$0.8437_{\pm 0.0067}$	$0.1219_{\pm 0.0125}$	$0.5244_{\pm 0.0179}$	$0.1727_{\pm 0.0074}$
	SWAG	$0.7705_{\pm 0.0188}$	$0.7235_{\pm 0.0528}$	$0.6984_{\pm 0.1431}$	$0.6970_{\pm 0.0585}$	$0.8556_{\pm 0.0162}$	$0.1275_{\pm 0.0085}$	$0.5070_{\pm 0.0303}$	$0.1652_{\pm 0.0099}$
	MCD	$0.7308_{\pm 0.0263}$	$0.6458_{\pm 0.0224}$	$0.7049_{\pm 0.1722}$	$0.6607_{\pm 0.0847}$	$0.8341_{\pm 0.0152}$	$0.0747_{\pm 0.0241}$	$0.4932_{\pm 0.0208}$	$0.1676_{\pm 0.0086}$
	DKL	$0.7487_{\pm 0.0416}$	$0.6602_{\pm 0.0606}$	$0.7574_{\pm 0.0953}$	$0.7008_{\pm 0.0504}$	$0.8061_{\pm 0.0224}$	$0.1135_{\pm 0.0240}$	$0.5526_{\pm 0.0384}$	$0.1800_{\pm 0.0140}$
	SGLD	$0.6949_{\pm 0.0231}$	$0.5971_{\pm 0.0273}$	$0.6787_{\pm 0.0321}$	$0.6350_{\pm 0.0261}$	$0.7433_{\pm 0.0208}$	$0.2037_{\pm 0.0282}$	$0.8403_{\pm 0.1110}$	$0.2379_{\pm 0.0193}$
ProstT5	Deterministic	$0.7013_{\pm 0.0065}$	$0.6241_{\pm 0.0193}$	$0.6033_{\pm 0.0706}$	$0.6104_{\pm 0.0282}$	$0.7668_{\pm 0.0137}$	$0.1796_{\pm 0.0642}$	$1.0011_{\pm 0.3935}$	$0.2255_{\pm 0.0248}$
	TS	$0.7013_{\pm 0.0065}$	$0.6241_{\pm 0.0193}$	$0.6033_{\pm 0.0706}$	$0.6104_{\pm 0.0282}$	$0.7668_{\pm 0.0137}$	$0.1644_{\pm 0.0499}$	$0.8615_{\pm 0.2806}$	$0.2197_{\pm 0.0222}$
	LA	$0.7000_{\pm 0.0285}$	$0.6559_{\pm 0.0814}$	$0.5279_{\pm 0.1292}$	$0.5716_{\pm 0.0701}$	$0.7619_{\pm 0.0102}$	$0.1689_{\pm 0.0602}$	$0.7930_{\pm 0.2498}$	$0.2221_{\pm 0.0245}$
	DVBLL	$0.6949_{\pm 0.0155}$	$0.6043_{\pm 0.0217}$	$0.6426_{\pm 0.0562}$	$0.6213_{\pm 0.0268}$	$0.7664_{\pm 0.0257}$	$0.2191_{\pm 0.0299}$	$0.9336_{\pm 0.1359}$	$0.2441_{\pm 0.0222}$
	EDL	$0.6795_{\pm 0.0378}$	$0.4922_{\pm 0.2463}$	$0.4787_{\pm 0.2470}$	$0.4842_{\pm 0.2445}$	$0.7373_{\pm 0.0462}$	$0.0717_{\pm 0.0121}$	$0.6015_{\pm 0.0335}$	$0.2069_{\pm 0.0150}$
	SWAG	$0.7192_{\pm 0.0179}$	$0.6234_{\pm 0.0147}$	$0.7115_{\pm 0.0644}$	$0.6633_{\pm 0.0332}$	$0.8028_{\pm 0.0205}$	$0.1196_{\pm 0.0113}$	$0.5694_{\pm 0.0377}$	$0.1880_{\pm 0.0115}$
	MCD	$0.7000_{\pm 0.0094}$	$0.6221_{\pm 0.0223}$	$0.6033_{\pm 0.0721}$	$0.6093_{\pm 0.0299}$	$0.7671_{\pm 0.0128}$	$0.1792_{\pm 0.0611}$	$0.9715_{\pm 0.3642}$	$0.2246_{\pm 0.0243}$
	DKL	$0.6949_{\pm 0.0224}$	$0.6274_{\pm 0.0233}$	$0.5377_{\pm 0.0964}$	$0.5751_{\pm 0.0602}$	$0.7251_{\pm 0.0402}$	$0.1611_{\pm 0.0487}$	$0.6982_{\pm 0.1190}$	$0.2232_{\pm 0.0270}$
	SGLD	$0.7154_{\pm 0.0087}$	$0.6190_{\pm 0.0097}$	$0.7082_{\pm 0.0191}$	$0.6605_{\pm 0.0118}$	$0.7776_{\pm 0.0026}$	$0.0848_{\pm 0.0124}$	$0.5572_{\pm 0.0076}$	$0.1862_{\pm 0.0010}$
Ankh	Deterministic	$0.7615_{\pm 0.0318}$	$0.7046_{\pm 0.0780}$	$0.7311_{\pm 0.1540}$	$0.6987_{\pm 0.0642}$	$0.8451_{\pm 0.0229}$	$0.0994_{\pm 0.0444}$	$0.5210_{\pm 0.0778}$	$0.1687_{\pm 0.0231}$
	TS	$0.7615_{\pm 0.0318}$	$0.7046_{\pm 0.0780}$	$0.7311_{\pm 0.1540}$	$0.6987_{\pm 0.0642}$	$0.8451_{\pm 0.0229}$	$0.1000_{\pm 0.0371}$	$0.5042_{\pm 0.0612}$	$0.1661_{\pm 0.0209}$
	LA	$0.7462_{\pm 0.0333}$	$0.7076_{\pm 0.0309}$	$0.6131_{\pm 0.1810}$	$0.6381_{\pm 0.1081}$	$0.8276_{\pm 0.0144}$	$0.1805_{\pm 0.0228}$	$0.7586_{\pm 0.0333}$	$0.2006_{\pm 0.0173}$
	DVBLL	$0.7282_{\pm 0.0605}$	$0.5144_{\pm 0.2576}$	$0.6885_{\pm 0.3464}$	$0.5882_{\pm 0.2944}$	$0.7661_{\pm 0.1373}$	$0.1314_{\pm 0.0496}$	$0.6086_{\pm 0.1190}$	$0.1925_{\pm 0.0410}$
	EDL	$0.6167_{\pm 0.1183}$	$0.4737_{\pm 0.2591}$	$0.4754_{\pm 0.3274}$	$0.4325_{\pm 0.2227}$	$0.7019_{\pm 0.1259}$	$0.1164_{\pm 0.0184}$	$0.6289_{\pm 0.0464}$	$0.2192_{\pm 0.0220}$
	SWAG	$0.7436_{\pm 0.0177}$	$0.6904_{\pm 0.0432}$	$0.6459_{\pm 0.1176}$	$0.6581_{\pm 0.0526}$	$0.8325_{\pm 0.0064}$	$0.1265_{\pm 0.0276}$	$0.6111_{\pm 0.0713}$	$0.1809_{\pm 0.0139}$
	MCD	$0.7615_{\pm 0.0398}$	$0.7027_{\pm 0.0745}$	$0.7311_{\pm 0.1605}$	$0.6980_{\pm 0.0757}$	$0.8446_{\pm 0.0232}$	$0.1033_{\pm 0.0404}$	$0.5175_{\pm 0.0741}$	$0.1680_{\pm 0.0230}$
	DKL	$0.7167_{\pm 0.0328}$	$0.6349_{\pm 0.0513}$	$0.6787_{\pm 0.0965}$	$0.6497_{\pm 0.0431}$	$0.7703_{\pm 0.0280}$	$0.1194_{\pm 0.0755}$	$0.6275_{\pm 0.0775}$	$0.2052_{\pm 0.0151}$
	SGLD	$0.7615_{\pm 0.0124}$	$0.6779_{\pm 0.0174}$	$0.7443_{\pm 0.0167}$	$0.7094_{\pm 0.0138}$	$0.8506_{\pm 0.0062}$	$0.0628_{\pm 0.0157}$	$0.4611_{\pm 0.0085}$	$0.1530_{\pm 0.0032}$
ESM2	Deterministic	$0.7500_{\pm 0.0266}$	$0.6487_{\pm 0.0278}$	$0.7869_{\pm 0.0427}$	$0.7109_{\pm 0.0323}$	$0.8241_{\pm 0.0107}$	$0.1217_{\pm 0.0110}$	$0.5398_{\pm 0.0154}$	$0.1703_{\pm 0.0065}$
	TS	$0.7500_{\pm 0.0266}$	$0.6487_{\pm 0.0278}$	$0.7869_{\pm 0.0427}$	$0.7109_{\pm 0.0323}$	$0.8241_{\pm 0.0107}$	$0.0952_{\pm 0.0163}$	$0.5196_{\pm 0.0157}$	$0.1672_{\pm 0.0067}$
	LA	$0.7513_{\pm 0.0174}$	$0.6871_{\pm 0.0399}$	$0.6852_{\pm 0.0751}$	$0.6819_{\pm 0.0229}$	$0.8255_{\pm 0.0074}$	$0.1233_{\pm 0.0430}$	$0.6226_{\pm 0.1342}$	$0.1841_{\pm 0.0169}$
	DVBLL	$0.7295_{\pm 0.0296}$	$0.6392_{\pm 0.0349}$	$0.7115_{\pm 0.0998}$	$0.6700_{\pm 0.0511}$	$0.8046_{\pm 0.0191}$	$0.1297_{\pm 0.0470}$	$0.6288_{\pm 0.1167}$	$0.1859_{\pm 0.0220}$
	EDL	$0.6936_{\pm 0.0192}$	$0.6017_{\pm 0.0177}$	$0.6361_{\pm 0.0706}$	$0.6170_{\pm 0.0403}$	$0.7816_{\pm 0.0215}$	$0.0832_{\pm 0.0151}$	$0.5639_{\pm 0.0223}$	$0.1909_{\pm 0.0093}$
	SWAG	$0.7577_{\pm 0.0238}$	$0.6623_{\pm 0.0333}$	$0.7869_{\pm 0.0803}$	$0.7163_{\pm 0.0323}$	$0.8202_{\pm 0.0160}$	$0.1252_{\pm 0.0553}$	$0.5556_{\pm 0.0514}$	$0.1827_{\pm 0.0172}$
	MCD	$0.7526_{\pm 0.0265}$	$0.6524_{\pm 0.0283}$	$0.7869_{\pm 0.0427}$	$0.7130_{\pm 0.0318}$	$0.8226_{\pm 0.0103}$	$0.1140_{\pm 0.0153}$	$0.5311_{\pm 0.0158}$	$0.1698_{\pm 0.0069}$
	DKL	$0.7551_{\pm 0.0143}$	$0.7010_{\pm 0.0646}$	$0.6984_{\pm 0.1671}$	$0.6792_{\pm 0.0769}$	$0.7901_{\pm 0.0301}$	$0.1516_{\pm 0.0636}$	$0.5972_{\pm 0.0541}$	$0.2043_{\pm 0.0241}$
	SGLD	$0.7128_{\pm 0.0148}$	$0.6198_{\pm 0.0190}$	$0.6885_{\pm 0.0147}$	$0.6523_{\pm 0.0158}$	$0.7960_{\pm 0.0058}$	$0.0782_{\pm 0.0156}$	$0.5275_{\pm 0.0061}$	$0.1806_{\pm 0.0029}$
Prot Bert	Deterministic	$0.7295_{\pm 0.0542}$	$0.6407_{\pm 0.0696}$	$0.7672_{\pm 0.0772}$	$0.6911_{\pm 0.0289}$	$0.7945_{\pm 0.0628}$	$0.1532_{\pm 0.0787}$	$0.6798_{\pm 0.2349}$	$0.2040_{\pm 0.0574}$
	TS	$0.7295_{\pm 0.0542}$	$0.6407_{\pm 0.0696}$	$0.7672_{\pm 0.0772}$	$0.6911_{\pm 0.0289}$	$0.7945_{\pm 0.0628}$	$0.1421_{\pm 0.0718}$	$0.6256_{\pm 0.1756}$	$0.1981_{\pm 0.0508}$
	LA	$0.7359_{\pm 0.0248}$	$0.6444_{\pm 0.0400}$	$0.7410_{\pm 0.0512}$	$0.6871_{\pm 0.0203}$	$0.7964_{\pm 0.0264}$	$0.1131_{\pm 0.0375}$	$0.5894_{\pm 0.0641}$	$0.1892_{\pm 0.0146}$
	DVBLL	$0.7333_{\pm 0.0271}$	$0.6381_{\pm 0.0437}$	$0.7508_{\pm 0.0318}$	$0.6883_{\pm 0.0204}$	$0.7947_{\pm 0.0288}$	$0.1420_{\pm 0.0277}$	$0.6521_{\pm 0.1097}$	$0.2014_{\pm 0.0226}$
	EDL	$0.6718_{\pm 0.0307}$	$0.5676_{\pm 0.0289}$	$0.6590_{\pm 0.0786}$	$0.6091_{\pm 0.0497}$	$0.7277_{\pm 0.0231}$	$0.0642_{\pm 0.0177}$	$0.5975_{\pm 0.0156}$	$0.2054_{\pm 0.0071}$
	SWAG	$0.7295_{\pm 0.0254}$	$0.6469_{\pm 0.0336}$	$0.6951_{\pm 0.1289}$	$0.6619_{\pm 0.0614}$	$0.8174_{\pm 0.0243}$	$0.1191_{\pm 0.0194}$	$0.5669_{\pm 0.0406}$	$0.1828_{\pm 0.0070}$
	MCD	$0.7295_{\pm 0.0509}$	$0.6406_{\pm 0.0668}$	$0.7672_{\pm 0.0864}$	$0.6904_{\pm 0.0274}$	$0.7946_{\pm 0.0613}$	$0.1541_{\pm 0.0799}$	$0.6710_{\pm 0.2239}$	$0.2031_{\pm 0.0559}$
	DKL	$0.7731_{\pm 0.0283}$	$0.7086_{\pm 0.0567}$	$0.7311_{\pm 0.0610}$	$0.7159_{\pm 0.0277}$	$0.7983_{\pm 0.0298}$	$0.1341_{\pm 0.0840}$	$0.5727_{\pm 0.0530}$	$0.1931_{\pm 0.0237}$
	SGLD	$0.7244_{\pm 0.0115}$	$0.6199_{\pm 0.0139}$	$0.7639_{\pm 0.0080}$	$0.6844_{\pm 0.0107}$	$0.7975_{\pm 0.0039}$	$0.0871_{\pm 0.0155}$	$0.5389_{\pm 0.0020}$	$0.1833_{\pm 0.0007}$
-	Ensemble	$0.7679_{\pm 0.0137}$	$0.6843_{\pm 0.0247}$	$0.7607_{\pm 0.0610}$	$0.7185_{\pm 0.0239}$	$0.8524_{\pm 0.0136}$	$0.0660_{\pm 0.0126}$	$0.4690_{\pm 0.0196}$	$0.1563_{\pm 0.0081}$

Table 4: Results on Immuno-Bacteria dataset of models trained on Immuno-Virus dataset.

PLM	Model	Accuracy	Precision	Recall	F1 Score	AUC ROC	ECE	NLL	Brier Score
ESMC	Deterministic	$0.6806_{\pm0.0089}$	$0.7662_{\pm0.1509}$	$0.1540_{\pm0.0361}$	$0.2499_{\pm0.0421}$	$0.6684_{\pm0.0463}$	$0.2647_{\pm0.0205}$	$1.4859_{\pm0.4486}$	$0.2753_{\pm0.0153}$
	TS	$0.6806_{\pm0.0089}$	$0.7662_{\pm0.1509}$	$0.1540_{\pm0.0361}$	$0.2499_{\pm0.0421}$	$0.6684_{\pm0.0463}$	$0.2470_{\pm0.0209}$	$1.2157_{\pm0.3334}$	$0.2659_{\pm0.0165}$
	LA	$0.6847_{\pm0.0161}$	$0.6819_{\pm0.0795}$	$0.1954_{\pm0.0262}$	$0.3025_{\pm0.0365}$	$0.6708_{\pm0.0334}$	$0.2432_{\pm0.0183}$	$1.2086_{\pm0.1842}$	$0.2659_{\pm0.0179}$
	DVBLL	$0.6847_{\pm0.0086}$	$0.6851_{\pm0.0532}$	$0.1908_{\pm0.0368}$	$0.2962_{\pm0.0438}$	$0.6465_{\pm0.0102}$	$0.2159_{\pm0.0491}$	$1.1928_{\pm0.5668}$	$0.2586_{\pm0.0211}$
	EDL	$0.6770_{\pm0.0122}$	$0.6240_{\pm0.0658}$	$0.2333_{\pm0.0814}$	$0.3275_{\pm0.0816}$	$0.6402_{\pm0.0287}$	$0.0880_{\pm0.0420}$	$0.6379_{\pm0.0316}$	$0.2216_{\pm0.0133}$
	SWAG	$0.6903_{\pm0.0175}$	$0.7831_{\pm0.0841}$	$0.1736_{\pm0.0862}$	$0.2714_{\pm0.1077}$	$0.6429_{\pm0.0358}$	$0.1395_{\pm0.0617}$	$0.7258_{\pm0.1516}$	$0.2282_{\pm0.0230}$
	MCD	$0.6806_{\pm0.0090}$	$0.7665_{\pm0.1510}$	$0.1540_{\pm0.0364}$	$0.2499_{\pm0.0429}$	$0.6661_{\pm0.0462}$	$0.2621_{\pm0.0187}$	$1.4174_{\pm0.4107}$	$0.2744_{\pm0.0151}$
	DKL	$0.6762_{\pm0.0270}$	$0.6165_{\pm0.1075}$	$0.2552_{\pm0.0232}$	$0.3560_{\pm0.0146}$	$0.6305_{\pm0.0421}$	$0.2130_{\pm0.0295}$	$0.8834_{\pm0.1169}$	$0.2602_{\pm0.0208}$
	SGLD	$0.6714_{\pm0.0167}$	$0.5763_{\pm0.0596}$	$0.2506_{\pm0.0232}$	$0.3484_{\pm0.0280}$	$0.6416_{\pm0.0286}$	$0.1718_{\pm0.0085}$	$0.8008_{\pm0.0312}$	$0.2430_{\pm0.0074}$
ProstT5	Deterministic	$0.6847_{\pm0.0178}$	$0.6534_{\pm0.0283}$	$0.2103_{\pm0.0851}$	$0.3099_{\pm0.0974}$	$0.7107_{\pm0.0263}$	$0.2333_{\pm0.0607}$	$1.1653_{\pm0.3833}$	$0.2625_{\pm0.0364}$
	TS	$0.6847_{\pm0.0178}$	$0.6534_{\pm0.0283}$	$0.2103_{\pm0.0851}$	$0.3099_{\pm0.0974}$	$0.7107_{\pm0.0263}$	$0.2125_{\pm0.0659}$	$0.9764_{\pm0.2722}$	$0.2528_{\pm0.0349}$
	LA	$0.7024_{\pm0.0067}$	$0.7112_{\pm0.0112}$	$0.2552_{\pm0.0273}$	$0.3748_{\pm0.0306}$	$0.7230_{\pm0.0222}$	$0.1861_{\pm0.0433}$	$0.8739_{\pm0.2163}$	$0.2342_{\pm0.0217}$
	DVBLL	$0.6863_{\pm0.0096}$	$0.6456_{\pm0.0314}$	$0.2368_{\pm0.0418}$	$0.3442_{\pm0.0451}$	$0.6994_{\pm0.0150}$	$0.2289_{\pm0.0450}$	$1.1927_{\pm0.3551}$	$0.2581_{\pm0.0211}$
	EDL	$0.7270_{\pm0.0175}$	$0.7917_{\pm0.0335}$	$0.3023_{\pm0.0631}$	$0.4334_{\pm0.0682}$	$0.7670_{\pm0.0312}$	$0.1116_{\pm0.0221}$	$0.5815_{\pm0.0318}$	$0.1949_{\pm0.0117}$
	SWAG	$0.6827_{\pm0.0049}$	$0.7845_{\pm0.0703}$	$0.1368_{\pm0.0307}$	$0.2304_{\pm0.0417}$	$0.7175_{\pm0.0291}$	$0.1894_{\pm0.0391}$	$0.7379_{\pm0.0795}$	$0.2355_{\pm0.0155}$
	MCD	$0.6851_{\pm0.0183}$	$0.6548_{\pm0.0265}$	$0.2115_{\pm0.0864}$	$0.3112_{\pm0.0980}$	$0.7103_{\pm0.0260}$	$0.2322_{\pm0.0604}$	$1.0995_{\pm0.3313}$	$0.2613_{\pm0.0358}$
	DKL	$0.6956_{\pm0.0165}$	$0.6219_{\pm0.0448}$	$0.3563_{\pm0.1206}$	$0.4394_{\pm0.0893}$	$0.6789_{\pm0.0376}$	$0.1770_{\pm0.0327}$	$0.7844_{\pm0.0957}$	$0.2371_{\pm0.0172}$
	SGLD	$0.7121_{\pm0.0109}$	$0.7913_{\pm0.0272}$	$0.2437_{\pm0.0374}$	$0.3710_{\pm0.0452}$	$0.7720_{\pm0.0155}$	$0.1567_{\pm0.0154}$	$0.6243_{\pm0.0366}$	$0.2030_{\pm0.0087}$
Ankh	Deterministic	$0.6907_{\pm0.0329}$	$0.6416_{\pm0.1107}$	$0.2713_{\pm0.0686}$	$0.3778_{\pm0.0820}$	$0.6999_{\pm0.0400}$	$0.2269_{\pm0.0381}$	$1.0845_{\pm0.3676}$	$0.2551_{\pm0.0298}$
	TS	$0.6907_{\pm0.0329}$	$0.6416_{\pm0.1107}$	$0.2713_{\pm0.0686}$	$0.3778_{\pm0.0820}$	$0.6999_{\pm0.0400}$	$0.2065_{\pm0.0392}$	$0.9310_{\pm0.2891}$	$0.2452_{\pm0.0284}$
	LA	$0.6601_{\pm0.0149}$	$0.5419_{\pm0.0627}$	$0.2494_{\pm0.0780}$	$0.3336_{\pm0.0620}$	$0.6459_{\pm0.0380}$	$0.1820_{\pm0.0314}$	$0.8314_{\pm0.0733}$	$0.2502_{\pm0.0127}$
	DVBLL	$0.5327_{\pm0.1453}$	$0.4560_{\pm0.0919}$	$0.5575_{\pm0.3651}$	$0.4062_{\pm0.1290}$	$0.5650_{\pm0.0634}$	$0.2112_{\pm0.0458}$	$0.9380_{\pm0.2341}$	$0.2708_{\pm0.0188}$
	EDL	$0.6843_{\pm0.0068}$	$0.6812_{\pm0.0240}$	$0.1874_{\pm0.0229}$	$0.2933_{\pm0.0289}$	$0.6852_{\pm0.0091}$	$0.1028_{\pm0.0089}$	$0.6261_{\pm0.0072}$	$0.2157_{\pm0.0030}$
	SWAG	$0.6766_{\pm0.0154}$	$0.6065_{\pm0.0799}$	$0.2782_{\pm0.1728}$	$0.3422_{\pm0.1644}$	$0.6822_{\pm0.0288}$	$0.1322_{\pm0.0711}$	$0.6971_{\pm0.0940}$	$0.2277_{\pm0.0161}$
	MCD	$0.6923_{\pm0.0332}$	$0.6487_{\pm0.1128}$	$0.2724_{\pm0.0688}$	$0.3800_{\pm0.0823}$	$0.7011_{\pm0.0388}$	$0.2243_{\pm0.0371}$	$1.0385_{\pm0.3176}$	$0.2538_{\pm0.0293}$
	DKL	$0.6407_{\pm0.0529}$	$0.5257_{\pm0.0653}$	$0.3954_{\pm0.2282}$	$0.4094_{\pm0.0843}$	$0.6596_{\pm0.0213}$	$0.2205_{\pm0.0485}$	$0.8581_{\pm0.1160}$	$0.2655_{\pm0.0262}$
	SGLD	$0.6911_{\pm0.0142}$	$0.6888_{\pm0.0459}$	$0.2172_{\pm0.0537}$	$0.3271_{\pm0.0635}$	$0.6977_{\pm0.0244}$	$0.1160_{\pm0.0207}$	$0.6383_{\pm0.0229}$	$0.2132_{\pm0.0076}$
ESM2	Deterministic	$0.6734_{\pm0.0298}$	$0.5907_{\pm0.1005}$	$0.3069_{\pm0.0847}$	$0.3913_{\pm0.0610}$	$0.6682_{\pm0.0461}$	$0.1870_{\pm0.0359}$	$0.8662_{\pm0.1500}$	$0.2473_{\pm0.0239}$
	TS	$0.6734_{\pm0.0298}$	$0.5907_{\pm0.1005}$	$0.3069_{\pm0.0847}$	$0.3913_{\pm0.0610}$	$0.6682_{\pm0.0461}$	$0.1631_{\pm0.0359}$	$0.7750_{\pm0.1072}$	$0.2383_{\pm0.0219}$
	LA	$0.6609_{\pm0.0376}$	$0.5545_{\pm0.0819}$	$0.3632_{\pm0.1195}$	$0.4187_{\pm0.0708}$	$0.6509_{\pm0.0408}$	$0.1787_{\pm0.0620}$	$1.0042_{\pm0.5591}$	$0.2499_{\pm0.0327}$
	DVBLL	$0.6306_{\pm0.0525}$	$0.5041_{\pm0.0766}$	$0.4736_{\pm0.1551}$	$0.4595_{\pm0.0864}$	$0.6301_{\pm0.0314}$	$0.1898_{\pm0.0422}$	$0.8859_{\pm0.2881}$	$0.2561_{\pm0.0222}$
	EDL	$0.6290_{\pm0.1394}$	$0.6278_{\pm0.1413}$	$0.3989_{\pm0.3016}$	$0.3965_{\pm0.0682}$	$0.6972_{\pm0.0490}$	$0.0949_{\pm0.0469}$	$0.6104_{\pm0.0427}$	$0.2114_{\pm0.0200}$
	SWAG	$0.6895_{\pm0.0122}$	$0.6566_{\pm0.0890}$	$0.3414_{\pm0.1960}$	$0.3990_{\pm0.1579}$	$0.6950_{\pm0.0167}$	$0.1315_{\pm0.0544}$	$0.6677_{\pm0.0832}$	$0.2201_{\pm0.0187}$
	MCD	$0.6738_{\pm0.0297}$	$0.5902_{\pm0.0971}$	$0.3069_{\pm0.0819}$	$0.3920_{\pm0.0595}$	$0.6681_{\pm0.0458}$	$0.1850_{\pm0.0361}$	$0.8538_{\pm0.1408}$	$0.2467_{\pm0.0237}$
	DKL	$0.6617_{\pm0.0191}$	$0.5559_{\pm0.0910}$	$0.3793_{\pm0.1316}$	$0.4259_{\pm0.0832}$	$0.6330_{\pm0.0502}$	$0.2261_{\pm0.0291}$	$0.9247_{\pm0.0999}$	$0.2703_{\pm0.0195}$
	SGLD	$0.7012_{\pm0.0213}$	$0.7344_{\pm0.0598}$	$0.2276_{\pm0.0688}$	$0.3433_{\pm0.0842}$	$0.7037_{\pm0.0306}$	$0.1037_{\pm0.0256}$	$0.6190_{\pm0.0371}$	$0.2085_{\pm0.0126}$
Prot Bert	Deterministic	$0.4306_{\pm0.0534}$	$0.3345_{\pm0.0179}$	$0.6161_{\pm0.0651}$	$0.4313_{\pm0.0104}$	$0.4851_{\pm0.0550}$	$0.4351_{\pm0.0738}$	$1.7388_{\pm0.3853}$	$0.4548_{\pm0.0618}$
	TS	$0.4306_{\pm0.0534}$	$0.3345_{\pm0.0179}$	$0.6161_{\pm0.0651}$	$0.4313_{\pm0.0104}$	$0.4851_{\pm0.0550}$	$0.4056_{\pm0.0750}$	$1.4520_{\pm0.2889}$	$0.4299_{\pm0.0596}$
	LA	$0.4661_{\pm0.0662}$	$0.3564_{\pm0.0241}$	$0.6218_{\pm0.0975}$	$0.4485_{\pm0.0208}$	$0.5079_{\pm0.0575}$	$0.3983_{\pm0.0957}$	$1.6074_{\pm0.5709}$	$0.4215_{\pm0.0821}$
	DVBLL	$0.5194_{\pm0.0516}$	$0.3761_{\pm0.0379}$	$0.5264_{\pm0.0926}$	$0.4325_{\pm0.0268}$	$0.5573_{\pm0.0476}$	$0.3537_{\pm0.0520}$	$1.4864_{\pm0.3260}$	$0.3804_{\pm0.0481}$
	EDL	$0.4444_{\pm0.0354}$	$0.3336_{\pm0.0148}$	$0.5782_{\pm0.0402}$	$0.4219_{\pm0.0071}$	$0.5077_{\pm0.0291}$	$0.3103_{\pm0.0237}$	$0.9232_{\pm0.0293}$	$0.3432_{\pm0.0149}$
	SWAG	$0.5540_{\pm0.0447}$	$0.3970_{\pm0.0330}$	$0.4920_{\pm0.1277}$	$0.4293_{\pm0.0509}$	$0.5654_{\pm0.0291}$	$0.2113_{\pm0.0314}$	$0.7909_{\pm0.0638}$	$0.2775_{\pm0.0245}$
	MCD	$0.4315_{\pm0.0534}$	$0.3349_{\pm0.0179}$	$0.6161_{\pm0.0651}$	$0.4316_{\pm0.0102}$	$0.4850_{\pm0.0544}$	$0.4338_{\pm0.0729}$	$1.6979_{\pm0.3756}$	$0.4526_{\pm0.0620}$
	DKL	$0.5129_{\pm0.0666}$	$0.3779_{\pm0.0385}$	$0.5678_{\pm0.0476}$	$0.4513_{\pm0.0290}$	$0.5427_{\pm0.0338}$	$0.3635_{\pm0.0726}$	$1.3199_{\pm0.2068}$	$0.3916_{\pm0.0546}$
	SGLD	$0.4532_{\pm0.0050}$	$0.3347_{\pm0.0073}$	$0.5667_{\pm0.0305}$	$0.4207_{\pm0.0139}$	$0.5058_{\pm0.0133}$	$0.2973_{\pm0.0122}$	$0.9108_{\pm0.0174}$	$0.3349_{\pm0.0065}$
-	Ensemble	$0.7069_{\pm0.0119}$	$0.8571_{\pm0.0696}$	$0.2069_{\pm0.0671}$	$0.3248_{\pm0.0790}$	$0.6846_{\pm0.0278}$	$0.1017_{\pm0.0208}$	$0.6356_{\pm0.0349}$	$0.2096_{\pm0.0102}$

Table 5: Results on Immuno-Tumor dataset of models trained on Immuno-Virus dataset.

PLM	Model	Accuracy	Precision	Recall	F1 Score	AUC ROC	ECE	NLL	Brier Score
ESMC	Deterministic	$0.5718_{\pm0.0487}$	$0.4439_{\pm0.0857}$	$0.4623_{\pm0.1434}$	$0.4480_{\pm0.1115}$	$0.5758_{\pm0.0763}$	$0.2638_{\pm0.0411}$	$1.0217_{\pm0.1702}$	$0.3031_{\pm0.0291}$
	TS	$0.5718_{\pm0.0487}$	$0.4439_{\pm0.0857}$	$0.4623_{\pm0.1434}$	$0.4480_{\pm0.1115}$	$0.5758_{\pm0.0763}$	$0.2529_{\pm0.0376}$	$0.9683_{\pm0.1416}$	$0.2979_{\pm0.0283}$
	LA	$0.6064_{\pm0.0496}$	$0.4912_{\pm0.0587}$	$0.5639_{\pm0.1378}$	$0.5219_{\pm0.0914}$	$0.6078_{\pm0.0666}$	$0.2440_{\pm0.0579}$	$0.9270_{\pm0.1515}$	$0.2874_{\pm0.0368}$
	DVBLL	$0.6295_{\pm0.0273}$	$0.5264_{\pm0.0415}$	$0.4623_{\pm0.1198}$	$0.4865_{\pm0.0796}$	$0.6572_{\pm0.0296}$	$0.2134_{\pm0.0775}$	$1.0070_{\pm0.3570}$	$0.2729_{\pm0.0421}$
	EDL	$0.6487_{\pm0.0258}$	$0.5457_{\pm0.0296}$	$0.6393_{\pm0.1178}$	$0.5833_{\pm0.0478}$	$0.6884_{\pm0.0444}$	$0.1370_{\pm0.0215}$	$0.6572_{\pm0.0211}$	$0.2311_{\pm0.0084}$
	SWAG	$0.5949_{\pm0.0404}$	$0.4949_{\pm0.0664}$	$0.5311_{\pm0.1564}$	$0.4979_{\pm0.0606}$	$0.6059_{\pm0.0633}$	$0.1865_{\pm0.0414}$	$0.7337_{\pm0.0520}$	$0.2585_{\pm0.0207}$
	MCD	$0.5718_{\pm0.0468}$	$0.4461_{\pm0.0796}$	$0.4656_{\pm0.1366}$	$0.4508_{\pm0.1043}$	$0.5764_{\pm0.0769}$	$0.2589_{\pm0.0395}$	$1.0056_{\pm0.1669}$	$0.3017_{\pm0.0295}$
	DKL	$0.5756_{\pm0.0400}$	$0.4657_{\pm0.0414}$	$0.5869_{\pm0.1373}$	$0.5135_{\pm0.0685}$	$0.5923_{\pm0.0657}$	$0.2619_{\pm0.0427}$	$0.9716_{\pm0.1251}$	$0.3090_{\pm0.0305}$
	SGLD	$0.5936_{\pm0.0445}$	$0.4881_{\pm0.0597}$	$0.5082_{\pm0.0769}$	$0.4932_{\pm0.0461}$	$0.5996_{\pm0.0284}$	$0.1985_{\pm0.0338}$	$0.8034_{\pm0.0383}$	$0.2755_{\pm0.0189}$
ProstT5	Deterministic	$0.5308_{\pm0.0234}$	$0.3204_{\pm0.0748}$	$0.1934_{\pm0.0699}$	$0.2391_{\pm0.0738}$	$0.4763_{\pm0.0227}$	$0.3237_{\pm0.0638}$	$1.3499_{\pm0.3352}$	$0.3524_{\pm0.0397}$
	TS	$0.5308_{\pm0.0234}$	$0.3204_{\pm0.0748}$	$0.1934_{\pm0.0699}$	$0.2391_{\pm0.0738}$	$0.4763_{\pm0.0227}$	$0.3105_{\pm0.0552}$	$1.2184_{\pm0.2389}$	$0.3436_{\pm0.0352}$
	LA	$0.5333_{\pm0.0340}$	$0.3794_{\pm0.0541}$	$0.2984_{\pm0.0553}$	$0.3321_{\pm0.0475}$	$0.4849_{\pm0.0137}$	$0.2933_{\pm0.0340}$	$1.1213_{\pm0.1855}$	$0.3360_{\pm0.0229}$
	DVBLL	$0.5410_{\pm0.0132}$	$0.3670_{\pm0.0322}$	$0.2459_{\pm0.0464}$	$0.2936_{\pm0.0422}$	$0.4773_{\pm0.0207}$	$0.3352_{\pm0.0482}$	$1.4934_{\pm0.3984}$	$0.3649_{\pm0.0306}$
	EDL	$0.5718_{\pm0.0174}$	$0.4310_{\pm0.0246}$	$0.2918_{\pm0.0523}$	$0.3450_{\pm0.0416}$	$0.5353_{\pm0.0202}$	$0.2163_{\pm0.0294}$	$0.8148_{\pm0.0503}$	$0.2903_{\pm0.0173}$
	SWAG	$0.5577_{\pm0.0260}$	$0.3441_{\pm0.0710}$	$0.1377_{\pm0.0447}$	$0.1924_{\pm0.0476}$	$0.4810_{\pm0.0220}$	$0.2444_{\pm0.0137}$	$0.9230_{\pm0.0495}$	$0.3068_{\pm0.0059}$
	MCD	$0.5346_{\pm0.0277}$	$0.3285_{\pm0.0818}$	$0.1967_{\pm0.0711}$	$0.2439_{\pm0.0765}$	$0.4757_{\pm0.0229}$	$0.3269_{\pm0.0614}$	$1.2952_{\pm0.2964}$	$0.3512_{\pm0.0391}$
	DKL	$0.5410_{\pm0.0519}$	$0.4135_{\pm0.0671}$	$0.3934_{\pm0.1000}$	$0.3972_{\pm0.0674}$	$0.5189_{\pm0.0595}$	$0.2918_{\pm0.0594}$	$1.0654_{\pm0.1979}$	$0.3385_{\pm0.0475}$
	SGLD	$0.5410_{\pm0.0305}$	$0.3664_{\pm0.0580}$	$0.2361_{\pm0.0353}$	$0.2869_{\pm0.0434}$	$0.4876_{\pm0.0233}$	$0.2439_{\pm0.0280}$	$0.9138_{\pm0.0498}$	$0.3138_{\pm0.0168}$
Ankh	Deterministic	$0.5192_{\pm0.0311}$	$0.4107_{\pm0.0259}$	$0.5148_{\pm0.0396}$	$0.4556_{\pm0.0205}$	$0.5193_{\pm0.0278}$	$0.3356_{\pm0.0261}$	$1.3002_{\pm0.1929}$	$0.3672_{\pm0.0137}$
	TS	$0.5192_{\pm0.0311}$	$0.4107_{\pm0.0259}$	$0.5148_{\pm0.0396}$	$0.4556_{\pm0.0205}$	$0.5193_{\pm0.0278}$	$0.3171_{\pm0.0229}$	$1.1768_{\pm0.1292}$	$0.3563_{\pm0.0134}$
	LA	$0.5064_{\pm0.0226}$	$0.4120_{\pm0.0196}$	$0.6197_{\pm0.0906}$	$0.4930_{\pm0.0387}$	$0.5230_{\pm0.0313}$	$0.3036_{\pm0.0229}$	$1.0624_{\pm0.0609}$	$0.3489_{\pm0.0118}$
	DVBLL	$0.4885_{\pm0.0841}$	$0.4232_{\pm0.0338}$	$0.7311_{\pm0.2228}$	$0.5185_{\pm0.0502}$	$0.5386_{\pm0.0515}$	$0.2456_{\pm0.0942}$	$0.9751_{\pm0.2538}$	$0.3083_{\pm0.0447}$
	EDL	$0.5756_{\pm0.0110}$	$0.4675_{\pm0.0108}$	$0.6131_{\pm0.0338}$	$0.5302_{\pm0.0164}$	$0.5836_{\pm0.0093}$	$0.1917_{\pm0.0259}$	$0.7523_{\pm0.0214}$	$0.2712_{\pm0.0085}$
	SWAG	$0.5205_{\pm0.0640}$	$0.4337_{\pm0.0337}$	$0.6295_{\pm0.1911}$	$0.4960_{\pm0.0566}$	$0.5649_{\pm0.0237}$	$0.2116_{\pm0.0743}$	$0.8459_{\pm0.1085}$	$0.2949_{\pm0.0370}$
	MCD	$0.5205_{\pm0.0288}$	$0.4112_{\pm0.0252}$	$0.5148_{\pm0.0493}$	$0.4558_{\pm0.0255}$	$0.5188_{\pm0.0286}$	$0.3320_{\pm0.0247}$	$1.2626_{\pm0.1734}$	$0.3655_{\pm0.0136}$
	DKL	$0.5218_{\pm0.0693}$	$0.4370_{\pm0.0350}$	$0.6623_{\pm0.1571}$	$0.5156_{\pm0.0343}$	$0.5564_{\pm0.0553}$	$0.2901_{\pm0.0378}$	$0.9949_{\pm0.0259}$	$0.3324_{\pm0.0226}$
	SGLD	$0.5641_{\pm0.0363}$	$0.4625_{\pm0.0279}$	$0.6721_{\pm0.0577}$	$0.5464_{\pm0.0276}$	$0.6074_{\pm0.0450}$	$0.1851_{\pm0.0288}$	$0.7685_{\pm0.0606}$	$0.2736_{\pm0.0234}$
ESM2	Deterministic	$0.5705_{\pm0.0194}$	$0.4648_{\pm0.0141}$	$0.6066_{\pm0.1150}$	$0.5201_{\pm0.0405}$	$0.5929_{\pm0.0234}$	$0.2363_{\pm0.0249}$	$0.9195_{\pm0.1060}$	$0.2968_{\pm0.0193}$
	TS	$0.5705_{\pm0.0194}$	$0.4648_{\pm0.0141}$	$0.6066_{\pm0.1150}$	$0.5201_{\pm0.0405}$	$0.5929_{\pm0.0234}$	$0.2268_{\pm0.0261}$	$0.8894_{\pm0.0872}$	$0.2929_{\pm0.0189}$
	LA	$0.5449_{\pm0.0339}$	$0.4423_{\pm0.0323}$	$0.6393_{\pm0.1248}$	$0.5190_{\pm0.0569}$	$0.5863_{\pm0.0703}$	$0.2661_{\pm0.0872}$	$1.1039_{\pm0.5269}$	$0.3147_{\pm0.0562}$
	DVBLL	$0.5167_{\pm0.0454}$	$0.4344_{\pm0.0158}$	$0.7148_{\pm0.2278}$	$0.5197_{\pm0.0811}$	$0.5886_{\pm0.0561}$	$0.2875_{\pm0.0213}$	$0.9562_{\pm0.1018}$	$0.3172_{\pm0.0106}$
	EDL	$0.5744_{\pm0.0941}$	$0.4900_{\pm0.0560}$	$0.7016_{\pm0.1539}$	$0.5632_{\pm0.0082}$	$0.6697_{\pm0.0077}$	$0.1638_{\pm0.0350}$	$0.6713_{\pm0.0344}$	$0.2385_{\pm0.0163}$
	SWAG	$0.5487_{\pm0.0514}$	$0.4365_{\pm0.0364}$	$0.4656_{\pm0.1674}$	$0.4304_{\pm0.0920}$	$0.5470_{\pm0.0297}$	$0.1921_{\pm0.0354}$	$0.8066_{\pm0.0720}$	$0.2806_{\pm0.0228}$
	MCD	$0.5692_{\pm0.0188}$	$0.4632_{\pm0.0144}$	$0.6066_{\pm0.1150}$	$0.5194_{\pm0.0418}$	$0.5935_{\pm0.0229}$	$0.2332_{\pm0.0258}$	$0.9136_{\pm0.1026}$	$0.2963_{\pm0.0191}$
	DKL	$0.5744_{\pm0.0419}$	$0.4674_{\pm0.0281}$	$0.5246_{\pm0.2042}$	$0.4740_{\pm0.0821}$	$0.5883_{\pm0.0569}$	$0.2936_{\pm0.0594}$	$1.0369_{\pm0.1757}$	$0.3215_{\pm0.0416}$
	SGLD	$0.6026_{\pm0.0167}$	$0.4944_{\pm0.0165}$	$0.6426_{\pm0.0191}$	$0.5585_{\pm0.0099}$	$0.6484_{\pm0.0086}$	$0.1587_{\pm0.0134}$	$0.6874_{\pm0.0069}$	$0.2427_{\pm0.0029}$
Prot Bert	Deterministic	$0.5436_{\pm0.0048}$	$0.4536_{\pm0.0091}$	$0.8393_{\pm0.1133}$	$0.5870_{\pm0.0375}$	$0.6548_{\pm0.0490}$	$0.3254_{\pm0.0215}$	$1.0835_{\pm0.0517}$	$0.3313_{\pm0.0076}$
	TS	$0.5436_{\pm0.0048}$	$0.4536_{\pm0.0091}$	$0.8393_{\pm0.1133}$	$0.5870_{\pm0.0375}$	$0.6548_{\pm0.0490}$	$0.3161_{\pm0.0168}$	$1.0031_{\pm0.0319}$	$0.3219_{\pm0.0058}$
	LA	$0.5526_{\pm0.0500}$	$0.4556_{\pm0.0427}$	$0.7934_{\pm0.1255}$	$0.5780_{\pm0.0673}$	$0.6100_{\pm0.0648}$	$0.3120_{\pm0.0244}$	$1.1458_{\pm0.2463}$	$0.3386_{\pm0.0212}$
	DVBLL	$0.5641_{\pm0.0278}$	$0.4590_{\pm0.0267}$	$0.6689_{\pm0.1064}$	$0.5422_{\pm0.0515}$	$0.5902_{\pm0.0433}$	$0.2932_{\pm0.0599}$	$1.2059_{\pm0.2588}$	$0.3367_{\pm0.0303}$
	EDL	$0.5705_{\pm0.0177}$	$0.4675_{\pm0.0142}$	$0.6721_{\pm0.0874}$	$0.5481_{\pm0.0280}$	$0.6000_{\pm0.0170}$	$0.1816_{\pm0.0050}$	$0.7535_{\pm0.0060}$	$0.2718_{\pm0.0023}$
	SWAG	$0.5731_{\pm0.0530}$	$0.4758_{\pm0.0404}$	$0.6328_{\pm0.1958}$	$0.5262_{\pm0.0634}$	$0.6321_{\pm0.0238}$	$0.1785_{\pm0.0802}$	$0.7349_{\pm0.0773}$	$0.2596_{\pm0.0297}$
	MCD	$0.5449_{\pm0.0041}$	$0.4549_{\pm0.0071}$	$0.8426_{\pm0.1075}$	$0.5890_{\pm0.0341}$	$0.6540_{\pm0.0502}$	$0.3231_{\pm0.0226}$	$1.0696_{\pm0.0487}$	$0.3304_{\pm0.0074}$
	DKL	$0.5321_{\pm0.0508}$	$0.4328_{\pm0.0471}$	$0.6393_{\pm0.1042}$	$0.5147_{\pm0.0611}$	$0.5582_{\pm0.0828}$	$0.3273_{\pm0.0636}$	$1.1504_{\pm0.1948}$	$0.3553_{\pm0.0504}$
	SGLD	$0.5808_{\pm0.0188}$	$0.4752_{\pm0.0153}$	$0.6820_{\pm0.0368}$	$0.5597_{\pm0.0184}$	$0.6061_{\pm0.0099}$	$0.1918_{\pm0.0107}$	$0.7884_{\pm0.0105}$	$0.2771_{\pm0.0044}$
-	Ensemble	$0.5987_{\pm0.0231}$	$0.4934_{\pm0.0226}$	$0.5475_{\pm0.1171}$	$0.5105_{\pm0.0458}$	$0.6108_{\pm0.0386}$	$0.1303_{\pm0.0330}$	$0.6925_{\pm0.0173}$	$0.2458_{\pm0.0075}$

Table 6: Results on Immuno-Virus dataset of models trained with Immuno-Bacteria dataset.

PLM	Model	Accuracy	Precision	Recall	F1 Score	AUC ROC	ECE	NLL	Brier Score
ESMC	Deterministic	0.5607$_{\pm 0.0462}$	0.5570$_{\pm 0.0415}$	0.7127$_{\pm 0.0523}$	0.6227$_{\pm 0.0205}$	0.6285$_{\pm 0.0599}$	0.1920$_{\pm 0.0352}$	0.8018$_{\pm 0.0861}$	0.2734$_{\pm 0.0258}$
	TS	0.5607$_{\pm 0.0462}$	0.5570$_{\pm 0.0415}$	0.7127$_{\pm 0.0523}$	0.6227$_{\pm 0.0205}$	0.6285$_{\pm 0.0599}$	0.1724$_{\pm 0.0273}$	0.7631$_{\pm 0.0622}$	0.2650$_{\pm 0.0209}$
	LA	0.5776$_{\pm 0.0191}$	0.5696$_{\pm 0.0170}$	0.6908$_{\pm 0.0516}$	0.6234$_{\pm 0.0226}$	0.6132$_{\pm 0.0367}$	0.2246$_{\pm 0.0822}$	1.0709$_{\pm 0.3643}$	0.3018$_{\pm 0.0458}$
	DVBLL	0.5655$_{\pm 0.0538}$	0.5647$_{\pm 0.0627}$	0.7404$_{\pm 0.0838}$	0.6340$_{\pm 0.0196}$	0.6247$_{\pm 0.0615}$	0.1896$_{\pm 0.0839}$	0.8598$_{\pm 0.2359}$	0.2772$_{\pm 0.0532}$
	EDL	0.5962$_{\pm 0.0711}$	0.6000$_{\pm 0.1048}$	0.7553$_{\pm 0.1051}$	0.6560$_{\pm 0.0378}$	0.6894$_{\pm 0.0535}$	0.1857$_{\pm 0.0415}$	0.7158$_{\pm 0.0637}$	0.2547$_{\pm 0.0289}$
	SWAG	0.5562$_{\pm 0.0357}$	0.5488$_{\pm 0.0267}$	0.7166$_{\pm 0.0731}$	0.6199$_{\pm 0.0362}$	0.6375$_{\pm 0.0514}$	0.2311$_{\pm 0.0524}$	0.8844$_{\pm 0.0701}$	0.2926$_{\pm 0.0157}$
	MCD	0.5612$_{\pm 0.0469}$	0.5575$_{\pm 0.0421}$	0.7136$_{\pm 0.0529}$	0.6233$_{\pm 0.0211}$	0.6285$_{\pm 0.0599}$	0.1893$_{\pm 0.0347}$	0.7973$_{\pm 0.0828}$	0.2728$_{\pm 0.0254}$
	DKL	0.5705$_{\pm 0.0348}$	0.5699$_{\pm 0.0523}$	0.7191$_{\pm 0.0979}$	0.6277$_{\pm 0.0213}$	0.6073$_{\pm 0.0403}$	0.2682$_{\pm 0.0557}$	0.9599$_{\pm 0.1491}$	0.3219$_{\pm 0.0383}$
	SGLD	0.5584$_{\pm 0.0120}$	0.5496$_{\pm 0.0091}$	0.7221$_{\pm 0.0449}$	0.6235$_{\pm 0.0187}$	0.5939$_{\pm 0.0200}$	0.2628$_{\pm 0.0298}$	1.0546$_{\pm 0.1528}$	0.3217$_{\pm 0.0236}$
ProstT5	Deterministic	0.5194$_{\pm 0.0297}$	0.5173$_{\pm 0.0182}$	0.7251$_{\pm 0.1116}$	0.6016$_{\pm 0.0487}$	0.5017$_{\pm 0.0201}$	0.3452$_{\pm 0.0495}$	1.4428$_{\pm 0.3252}$	0.3870$_{\pm 0.0360}$
	TS	0.5194$_{\pm 0.0297}$	0.5173$_{\pm 0.0182}$	0.7251$_{\pm 0.1116}$	0.6016$_{\pm 0.0487}$	0.5017$_{\pm 0.0201}$	0.3155$_{\pm 0.0517}$	1.2261$_{\pm 0.2370}$	0.3681$_{\pm 0.0347}$
	LA	0.5383$_{\pm 0.0152}$	0.5338$_{\pm 0.0144}$	0.7454$_{\pm 0.1089}$	0.6177$_{\pm 0.0378}$	0.5128$_{\pm 0.0344}$	0.2402$_{\pm 0.0576}$	1.0077$_{\pm 0.1656}$	0.3264$_{\pm 0.0354}$
	DVBLL	0.5116$_{\pm 0.0156}$	0.5175$_{\pm 0.0163}$	0.6645$_{\pm 0.1376}$	0.5731$_{\pm 0.0567}$	0.5271$_{\pm 0.0279}$	0.3214$_{\pm 0.0937}$	1.4097$_{\pm 0.5987}$	0.3725$_{\pm 0.0604}$
	EDL	0.5330$_{\pm 0.0163}$	0.5303$_{\pm 0.0139}$	0.7533$_{\pm 0.1269}$	0.6175$_{\pm 0.0306}$	0.6131$_{\pm 0.0224}$	0.2029$_{\pm 0.0473}$	0.7807$_{\pm 0.0406}$	0.2843$_{\pm 0.0147}$
	SWAG	0.5232$_{\pm 0.0309}$	0.5289$_{\pm 0.0353}$	0.6566$_{\pm 0.0739}$	0.5817$_{\pm 0.0205}$	0.5347$_{\pm 0.0571}$	0.2519$_{\pm 0.0550}$	0.9333$_{\pm 0.1240}$	0.3189$_{\pm 0.0264}$
	MCD	0.5199$_{\pm 0.0308}$	0.5175$_{\pm 0.0189}$	0.7251$_{\pm 0.1136}$	0.6017$_{\pm 0.0499}$	0.5015$_{\pm 0.0199}$	0.3435$_{\pm 0.0488}$	1.3980$_{\pm 0.2957}$	0.3850$_{\pm 0.0354}$
	DKL	0.5338$_{\pm 0.0121}$	0.5270$_{\pm 0.0083}$	0.7955$_{\pm 0.0419}$	0.6336$_{\pm 0.0148}$	0.5346$_{\pm 0.0291}$	0.2470$_{\pm 0.0259}$	0.8842$_{\pm 0.0459}$	0.3160$_{\pm 0.0120}$
	SGLD	0.5343$_{\pm 0.0090}$	0.5288$_{\pm 0.0057}$	0.7524$_{\pm 0.0280}$	0.6210$_{\pm 0.0131}$	0.5435$_{\pm 0.0424}$	0.2071$_{\pm 0.0149}$	0.8039$_{\pm 0.0372}$	0.2926$_{\pm 0.0118}$
Ankh	Deterministic	0.5025$_{\pm 0.0330}$	0.5270$_{\pm 0.0607}$	0.5990$_{\pm 0.1759}$	0.5353$_{\pm 0.0828}$	0.4955$_{\pm 0.0591}$	0.2765$_{\pm 0.0293}$	1.0302$_{\pm 0.0873}$	0.3443$_{\pm 0.0190}$
	TS	0.5025$_{\pm 0.0330}$	0.5270$_{\pm 0.0607}$	0.5990$_{\pm 0.1759}$	0.5353$_{\pm 0.0828}$	0.4955$_{\pm 0.0591}$	0.2471$_{\pm 0.0249}$	0.9340$_{\pm 0.0611}$	0.3258$_{\pm 0.0166}$
	LA	0.4922$_{\pm 0.0491}$	0.5218$_{\pm 0.0811}$	0.5509$_{\pm 0.2357}$	0.4931$_{\pm 0.1421}$	0.4801$_{\pm 0.0896}$	0.2666$_{\pm 0.1096}$	1.0850$_{\pm 0.4920}$	0.3413$_{\pm 0.0790}$
	DVBLL	0.5020$_{\pm 0.0151}$	0.3083$_{\pm 0.2520}$	0.2774$_{\pm 0.3424}$	0.2396$_{\pm 0.2845}$	0.5034$_{\pm 0.0167}$	0.1396$_{\pm 0.0729}$	0.8091$_{\pm 0.1824}$	0.2781$_{\pm 0.0327}$
	EDL	0.5033$_{\pm 0.0341}$	0.5090$_{\pm 0.0263}$	0.6998$_{\pm 0.0414}$	0.5885$_{\pm 0.0228}$	0.5273$_{\pm 0.0676}$	0.0981$_{\pm 0.0491}$	0.7306$_{\pm 0.0355}$	0.2626$_{\pm 0.0095}$
	SWAG	0.5290$_{\pm 0.0362}$	0.5338$_{\pm 0.0323}$	0.6020$_{\pm 0.1634}$	0.5535$_{\pm 0.0851}$	0.5560$_{\pm 0.0565}$	0.2007$_{\pm 0.0329}$	0.8372$_{\pm 0.0577}$	0.2912$_{\pm 0.0128}$
	MCD	0.5015$_{\pm 0.0340}$	0.5268$_{\pm 0.0627}$	0.5985$_{\pm 0.1761}$	0.5346$_{\pm 0.0824}$	0.4945$_{\pm 0.0596}$	0.2729$_{\pm 0.0298}$	1.0211$_{\pm 0.0905}$	0.3427$_{\pm 0.0192}$
	DKL	0.5146$_{\pm 0.0265}$	0.5341$_{\pm 0.0496}$	0.5290$_{\pm 0.1280}$	0.5173$_{\pm 0.0552}$	0.5257$_{\pm 0.0365}$	0.3006$_{\pm 0.0406}$	1.1126$_{\pm 0.1461}$	0.3533$_{\pm 0.0268}$
	SGLD	0.4907$_{\pm 0.0153}$	0.4973$_{\pm 0.0129}$	0.5449$_{\pm 0.0702}$	0.5186$_{\pm 0.0383}$	0.5169$_{\pm 0.0100}$	0.1761$_{\pm 0.0119}$	0.7514$_{\pm 0.0088}$	0.2747$_{\pm 0.0042}$
ESM2	Deterministic	0.5673$_{\pm 0.0326}$	0.5866$_{\pm 0.0698}$	0.5836$_{\pm 0.0903}$	0.5752$_{\pm 0.0291}$	0.6084$_{\pm 0.0265}$	0.1606$_{\pm 0.0335}$	0.7789$_{\pm 0.0474}$	0.2689$_{\pm 0.0137}$
	TS	0.5673$_{\pm 0.0326}$	0.5866$_{\pm 0.0698}$	0.5836$_{\pm 0.0903}$	0.5752$_{\pm 0.0291}$	0.6084$_{\pm 0.0265}$	0.1458$_{\pm 0.0278}$	0.7491$_{\pm 0.0331}$	0.2629$_{\pm 0.0108}$
	LA	0.5355$_{\pm 0.0250}$	0.5316$_{\pm 0.0188}$	0.7206$_{\pm 0.1346}$	0.6059$_{\pm 0.0579}$	0.5685$_{\pm 0.0574}$	0.2257$_{\pm 0.0915}$	0.9459$_{\pm 0.2227}$	0.3110$_{\pm 0.0511}$
	DVBLL	0.5295$_{\pm 0.0251}$	0.5272$_{\pm 0.0193}$	0.7350$_{\pm 0.1186}$	0.6095$_{\pm 0.0438}$	0.5239$_{\pm 0.0292}$	0.3276$_{\pm 0.0532}$	1.4240$_{\pm 0.3545}$	0.3696$_{\pm 0.0364}$
	EDL	0.5254$_{\pm 0.0175}$	0.5202$_{\pm 0.0124}$	0.8978$_{\pm 0.0862}$	0.6566$_{\pm 0.0142}$	0.5390$_{\pm 0.0531}$	0.1185$_{\pm 0.1015}$	0.7536$_{\pm 0.0764}$	0.2729$_{\pm 0.0288}$
	SWAG	0.5652$_{\pm 0.0137}$	0.5529$_{\pm 0.0095}$	0.7519$_{\pm 0.0732}$	0.6357$_{\pm 0.0267}$	0.5742$_{\pm 0.0329}$	0.2370$_{\pm 0.0562}$	0.9374$_{\pm 0.1943}$	0.3067$_{\pm 0.0338}$
	MCD	0.5680$_{\pm 0.0337}$	0.5876$_{\pm 0.0718}$	0.5851$_{\pm 0.0889}$	0.5764$_{\pm 0.0275}$	0.6086$_{\pm 0.0266}$	0.1604$_{\pm 0.0328}$	0.7766$_{\pm 0.0464}$	0.2686$_{\pm 0.0136}$
	DKL	0.5393$_{\pm 0.0367}$	0.5544$_{\pm 0.0613}$	0.5990$_{\pm 0.1000}$	0.5657$_{\pm 0.0327}$	0.5596$_{\pm 0.0691}$	0.2626$_{\pm 0.0776}$	0.9900$_{\pm 0.2066}$	0.3236$_{\pm 0.0475}$
	SGLD	0.5683$_{\pm 0.0093}$	0.5535$_{\pm 0.0058}$	0.7712$_{\pm 0.0247}$	0.6444$_{\pm 0.0121}$	0.5736$_{\pm 0.0138}$	0.1452$_{\pm 0.0037}$	0.7294$_{\pm 0.0104}$	0.2634$_{\pm 0.0041}$
Prot Bert	Deterministic	0.3914$_{\pm 0.1150}$	0.4207$_{\pm 0.1297}$	0.2605$_{\pm 0.0609}$	0.3012$_{\pm 0.0178}$	0.2929$_{\pm 0.0637}$	0.4371$_{\pm 0.0969}$	1.4532$_{\pm 0.4295}$	0.4482$_{\pm 0.0700}$
	TS	0.3914$_{\pm 0.1150}$	0.4207$_{\pm 0.1297}$	0.2605$_{\pm 0.0609}$	0.3012$_{\pm 0.0178}$	0.2929$_{\pm 0.0637}$	0.4038$_{\pm 0.0993}$	1.2577$_{\pm 0.3179}$	0.4201$_{\pm 0.0648}$
	LA	0.3199$_{\pm 0.0510}$	0.3210$_{\pm 0.0349}$	0.2988$_{\pm 0.0548}$	0.3068$_{\pm 0.0355}$	0.2702$_{\pm 0.0410}$	0.4593$_{\pm 0.0498}$	1.6288$_{\pm 0.4973}$	0.4665$_{\pm 0.0518}$
	DVBLL	0.3108$_{\pm 0.0402}$	0.3088$_{\pm 0.0169}$	0.2854$_{\pm 0.0429}$	0.2944$_{\pm 0.0223}$	0.2579$_{\pm 0.0247}$	0.4320$_{\pm 0.0540}$	1.6157$_{\pm 0.5023}$	0.4475$_{\pm 0.0579}$
	EDL	0.3630$_{\pm 0.0683}$	0.3842$_{\pm 0.1482}$	0.2447$_{\pm 0.1524}$	0.2479$_{\pm 0.1344}$	0.2381$_{\pm 0.0319}$	0.3697$_{\pm 0.0554}$	1.0027$_{\pm 0.1108}$	0.3792$_{\pm 0.0427}$
	SWAG	0.3547$_{\pm 0.0601}$	0.3525$_{\pm 0.0489}$	0.3136$_{\pm 0.0800}$	0.3261$_{\pm 0.0551}$	0.2891$_{\pm 0.0483}$	0.3950$_{\pm 0.0599}$	1.1951$_{\pm 0.2666}$	0.4118$_{\pm 0.0523}$
	MCD	0.3912$_{\pm 0.1138}$	0.4182$_{\pm 0.1264}$	0.2600$_{\pm 0.0610}$	0.3007$_{\pm 0.0184}$	0.2924$_{\pm 0.0637}$	0.4363$_{\pm 0.0965}$	1.4300$_{\pm 0.4050}$	0.4464$_{\pm 0.0695}$
	DKL	0.3091$_{\pm 0.0284}$	0.3215$_{\pm 0.0222}$	0.3270$_{\pm 0.0501}$	0.3230$_{\pm 0.0326}$	0.2587$_{\pm 0.0252}$	0.4994$_{\pm 0.0350}$	1.4579$_{\pm 0.2055}$	0.4922$_{\pm 0.0375}$
	SGLD	0.3222$_{\pm 0.0203}$	0.3306$_{\pm 0.0142}$	0.3261$_{\pm 0.0098}$	0.3282$_{\pm 0.0093}$	0.2786$_{\pm 0.0089}$	0.4166$_{\pm 0.0121}$	1.1247$_{\pm 0.0376}$	0.4096$_{\pm 0.0102}$
-	Ensemble	0.4861$_{\pm 0.0321}$	0.4977$_{\pm 0.0281}$	0.5960$_{\pm 0.0462}$	0.5405$_{\pm 0.0138}$	0.4821$_{\pm 0.0575}$	0.1953$_{\pm 0.0342}$	0.7888$_{\pm 0.0244}$	0.2889$_{\pm 0.0144}$

Table 7: Results on Immuno-Tumor dataset of models trained with Immuno-Bacteria dataset.

PLM	Model	Accuracy	Precision	Recall	F1 Score	AUC ROC	ECE	NLL	Brier Score
ESMC	Deterministic	$0.5641_{\pm0.0226}$	$0.4174_{\pm0.0460}$	$0.2951_{\pm0.0803}$	$0.3414_{\pm0.0602}$	$0.4903_{\pm0.0397}$	$0.1942_{\pm0.0633}$	$0.8803_{\pm0.1677}$	$0.2890_{\pm0.0310}$
	TS	$0.5641_{\pm0.0226}$	$0.4174_{\pm0.0460}$	$0.2951_{\pm0.0803}$	$0.3414_{\pm0.0602}$	$0.4903_{\pm0.0397}$	$0.1962_{\pm0.0623}$	$0.8434_{\pm0.1262}$	$0.2848_{\pm0.0269}$
	LA	$0.5410_{\pm0.0065}$	$0.3581_{\pm0.0763}$	$0.2885_{\pm0.1439}$	$0.3099_{\pm0.1212}$	$0.4792_{\pm0.0483}$	$0.2798_{\pm0.0786}$	$1.2244_{\pm0.3474}$	$0.3338_{\pm0.0386}$
	DVBLL	$0.5321_{\pm0.0608}$	$0.4176_{\pm0.0528}$	$0.4525_{\pm0.1401}$	$0.4219_{\pm0.0718}$	$0.4992_{\pm0.0526}$	$0.2439_{\pm0.0987}$	$1.0103_{\pm0.3318}$	$0.3150_{\pm0.0583}$
	EDL	$0.5654_{\pm0.0338}$	$0.4461_{\pm0.0366}$	$0.4557_{\pm0.1850}$	$0.4315_{\pm0.0991}$	$0.5553_{\pm0.0492}$	$0.2204_{\pm0.0431}$	$0.7967_{\pm0.0485}$	$0.2846_{\pm0.0168}$
	SWAG	$0.5333_{\pm0.0502}$	$0.4134_{\pm0.0404}$	$0.4230_{\pm0.1250}$	$0.4079_{\pm0.0509}$	$0.5193_{\pm0.0220}$	$0.2582_{\pm0.0744}$	$0.9776_{\pm0.2139}$	$0.3133_{\pm0.0384}$
	MCD	$0.5641_{\pm0.0226}$	$0.4184_{\pm0.0456}$	$0.2984_{\pm0.0792}$	$0.3441_{\pm0.0592}$	$0.4918_{\pm0.0386}$	$0.1957_{\pm0.0634}$	$0.8742_{\pm0.1625}$	$0.2881_{\pm0.0308}$
	DKL	$0.5013_{\pm0.0366}$	$0.3310_{\pm0.0658}$	$0.2787_{\pm0.0933}$	$0.2976_{\pm0.0747}$	$0.4558_{\pm0.0520}$	$0.3366_{\pm0.0487}$	$1.0725_{\pm0.1522}$	$0.3616_{\pm0.0402}$
	SGLD	$0.5462_{\pm0.0333}$	$0.4130_{\pm0.0535}$	$0.3967_{\pm0.0751}$	$0.4038_{\pm0.0630}$	$0.5169_{\pm0.0360}$	$0.2963_{\pm0.0365}$	$1.2480_{\pm0.1691}$	$0.3434_{\pm0.0255}$
ProstT5	Deterministic	$0.5962_{\pm0.0295}$	$0.4821_{\pm0.0374}$	$0.3967_{\pm0.1206}$	$0.4241_{\pm0.0838}$	$0.5935_{\pm0.0237}$	$0.2578_{\pm0.0289}$	$1.0435_{\pm0.1574}$	$0.2976_{\pm0.0074}$
	TS	$0.5962_{\pm0.0295}$	$0.4821_{\pm0.0374}$	$0.3967_{\pm0.1206}$	$0.4241_{\pm0.0838}$	$0.5935_{\pm0.0237}$	$0.2423_{\pm0.0209}$	$0.9491_{\pm0.0973}$	$0.2895_{\pm0.0036}$
	LA	$0.5603_{\pm0.0294}$	$0.4394_{\pm0.0326}$	$0.4230_{\pm0.1123}$	$0.4218_{\pm0.0615}$	$0.5756_{\pm0.0322}$	$0.2141_{\pm0.0441}$	$0.8586_{\pm0.1183}$	$0.2829_{\pm0.0251}$
	DVBLL	$0.5910_{\pm0.0196}$	$0.4868_{\pm0.0397}$	$0.3934_{\pm0.1360}$	$0.4132_{\pm0.0946}$	$0.5901_{\pm0.0356}$	$0.2564_{\pm0.0626}$	$1.1379_{\pm0.3927}$	$0.3104_{\pm0.0354}$
	EDL	$0.5731_{\pm0.0916}$	$0.4917_{\pm0.0527}$	$0.5475_{\pm0.2439}$	$0.4835_{\pm0.0717}$	$0.6003_{\pm0.0233}$	$0.1833_{\pm0.0454}$	$0.7476_{\pm0.0562}$	$0.2623_{\pm0.0177}$
	SWAG	$0.5910_{\pm0.0208}$	$0.4747_{\pm0.0308}$	$0.3934_{\pm0.1067}$	$0.4218_{\pm0.0635}$	$0.5822_{\pm0.0315}$	$0.2133_{\pm0.0717}$	$0.8919_{\pm0.2031}$	$0.2835_{\pm0.0374}$
	MCD	$0.5962_{\pm0.0306}$	$0.4831_{\pm0.0375}$	$0.3934_{\pm0.1168}$	$0.4226_{\pm0.0809}$	$0.5940_{\pm0.0234}$	$0.2590_{\pm0.0239}$	$1.0219_{\pm0.1435}$	$0.2963_{\pm0.0071}$
	DKL	$0.5821_{\pm0.0159}$	$0.4545_{\pm0.0280}$	$0.3508_{\pm0.0338}$	$0.3958_{\pm0.0317}$	$0.5286_{\pm0.0328}$	$0.2003_{\pm0.0126}$	$0.7991_{\pm0.0305}$	$0.2823_{\pm0.0071}$
	SGLD	$0.6385_{\pm0.0138}$	$0.5411_{\pm0.0204}$	$0.5049_{\pm0.0241}$	$0.5219_{\pm0.0165}$	$0.6085_{\pm0.0089}$	$0.1422_{\pm0.0135}$	$0.7144_{\pm0.0215}$	$0.2468_{\pm0.0062}$
Ankh	Deterministic	$0.5872_{\pm0.0258}$	$0.4487_{\pm0.0859}$	$0.2754_{\pm0.1206}$	$0.3287_{\pm0.1039}$	$0.5369_{\pm0.0416}$	$0.2566_{\pm0.0450}$	$1.0967_{\pm0.1971}$	$0.3083_{\pm0.0280}$
	TS	$0.5872_{\pm0.0258}$	$0.4487_{\pm0.0859}$	$0.2754_{\pm0.1206}$	$0.3287_{\pm0.1039}$	$0.5369_{\pm0.0416}$	$0.2319_{\pm0.0524}$	$0.9907_{\pm0.1490}$	$0.2986_{\pm0.0257}$
	LA	$0.5795_{\pm0.0313}$	$0.4501_{\pm0.0626}$	$0.3016_{\pm0.1424}$	$0.3384_{\pm0.1224}$	$0.5452_{\pm0.0645}$	$0.2209_{\pm0.0617}$	$0.9416_{\pm0.2189}$	$0.2888_{\pm0.0210}$
	DVBLL	$0.5821_{\pm0.0477}$	$0.1753_{\pm0.2182}$	$0.1443_{\pm0.1910}$	$0.1500_{\pm0.1859}$	$0.5014_{\pm0.0318}$	$0.1210_{\pm0.1076}$	$0.8654_{\pm0.3357}$	$0.2666_{\pm0.0357}$
	EDL	$0.5590_{\pm0.0273}$	$0.3042_{\pm0.0979}$	$0.1574_{\pm0.1378}$	$0.1897_{\pm0.1358}$	$0.4835_{\pm0.0440}$	$0.1548_{\pm0.0601}$	$0.7492_{\pm0.0742}$	$0.2660_{\pm0.0198}$
	SWAG	$0.5705_{\pm0.0521}$	$0.4605_{\pm0.0768}$	$0.3443_{\pm0.1410}$	$0.3733_{\pm0.0618}$	$0.5460_{\pm0.0444}$	$0.1965_{\pm0.0974}$	$0.8679_{\pm0.1854}$	$0.2879_{\pm0.0416}$
	MCD	$0.5859_{\pm0.0258}$	$0.4428_{\pm0.0923}$	$0.2721_{\pm0.1255}$	$0.3236_{\pm0.1105}$	$0.5367_{\pm0.0414}$	$0.2557_{\pm0.0467}$	$1.0828_{\pm0.1957}$	$0.3076_{\pm0.0281}$
	DKL	$0.5795_{\pm0.0252}$	$0.4421_{\pm0.0414}$	$0.2689_{\pm0.0627}$	$0.3297_{\pm0.0466}$	$0.5595_{\pm0.0463}$	$0.2635_{\pm0.0408}$	$1.0181_{\pm0.1498}$	$0.3141_{\pm0.0268}$
	SGLD	$0.5936_{\pm0.0119}$	$0.4474_{\pm0.0388}$	$0.1639_{\pm0.0232}$	$0.2392_{\pm0.0284}$	$0.5408_{\pm0.0108}$	$0.1529_{\pm0.0125}$	$0.7455_{\pm0.0076}$	$0.2598_{\pm0.0031}$
ESM2	Deterministic	$0.6013_{\pm0.0234}$	$0.4729_{\pm0.0860}$	$0.1967_{\pm0.0647}$	$0.2735_{\pm0.0749}$	$0.5603_{\pm0.0556}$	$0.1959_{\pm0.0629}$	$0.8330_{\pm0.0929}$	$0.2771_{\pm0.0253}$
	TS	$0.6013_{\pm0.0234}$	$0.4729_{\pm0.0860}$	$0.1967_{\pm0.0647}$	$0.2735_{\pm0.0749}$	$0.5603_{\pm0.0556}$	$0.1940_{\pm0.0506}$	$0.8030_{\pm0.0701}$	$0.2726_{\pm0.0213}$
	LA	$0.5718_{\pm0.0717}$	$0.4921_{\pm0.1098}$	$0.5115_{\pm0.0997}$	$0.4814_{\pm0.0227}$	$0.5652_{\pm0.0481}$	$0.2368_{\pm0.0974}$	$0.9164_{\pm0.2232}$	$0.2996_{\pm0.0603}$
	DVBLL	$0.5218_{\pm0.0422}$	$0.4046_{\pm0.0232}$	$0.4525_{\pm0.1487}$	$0.4153_{\pm0.0583}$	$0.5364_{\pm0.0448}$	$0.3103_{\pm0.0459}$	$1.2020_{\pm0.1811}$	$0.3545_{\pm0.0310}$
	EDL	$0.5000_{\pm0.0924}$	$0.4351_{\pm0.0457}$	$0.7311_{\pm0.2422}$	$0.5228_{\pm0.0521}$	$0.5493_{\pm0.0490}$	$0.1562_{\pm0.0593}$	$0.7332_{\pm0.0611}$	$0.2640_{\pm0.0229}$
	SWAG	$0.5538_{\pm0.0481}$	$0.4484_{\pm0.0540}$	$0.5246_{\pm0.0518}$	$0.4795_{\pm0.0260}$	$0.5679_{\pm0.0410}$	$0.2260_{\pm0.0870}$	$0.9231_{\pm0.2300}$	$0.3046_{\pm0.0524}$
	MCD	$0.6026_{\pm0.0256}$	$0.4791_{\pm0.0968}$	$0.1967_{\pm0.0647}$	$0.2743_{\pm0.0754}$	$0.5593_{\pm0.0555}$	$0.1939_{\pm0.0620}$	$0.8295_{\pm0.0910}$	$0.2765_{\pm0.0250}$
	DKL	$0.5551_{\pm0.0419}$	$0.4290_{\pm0.0470}$	$0.4328_{\pm0.1221}$	$0.4253_{\pm0.0763}$	$0.5432_{\pm0.0486}$	$0.2492_{\pm0.0751}$	$0.9320_{\pm0.1744}$	$0.3091_{\pm0.0428}$
	SGLD	$0.6167_{\pm0.0143}$	$0.5111_{\pm0.0207}$	$0.4623_{\pm0.0997}$	$0.4794_{\pm0.0618}$	$0.5971_{\pm0.0212}$	$0.1232_{\pm0.0104}$	$0.6976_{\pm0.0118}$	$0.2456_{\pm0.0043}$
Prot Bert	Deterministic	$0.5410_{\pm0.0534}$	$0.3911_{\pm0.0484}$	$0.2426_{\pm0.0941}$	$0.2807_{\pm0.0658}$	$0.4679_{\pm0.0568}$	$0.2808_{\pm0.0562}$	$1.0681_{\pm0.2285}$	$0.3304_{\pm0.0347}$
	TS	$0.5410_{\pm0.0534}$	$0.3911_{\pm0.0484}$	$0.2426_{\pm0.0941}$	$0.2807_{\pm0.0658}$	$0.4679_{\pm0.0568}$	$0.2684_{\pm0.0471}$	$0.9990_{\pm0.1562}$	$0.3242_{\pm0.0300}$
	LA	$0.5346_{\pm0.0371}$	$0.3752_{\pm0.0561}$	$0.2721_{\pm0.0644}$	$0.3108_{\pm0.0471}$	$0.4610_{\pm0.0262}$	$0.2618_{\pm0.0535}$	$1.0941_{\pm0.2567}$	$0.3210_{\pm0.0276}$
	DVBLL	$0.5603_{\pm0.0209}$	$0.3982_{\pm0.0762}$	$0.2951_{\pm0.1032}$	$0.3344_{\pm0.0979}$	$0.4919_{\pm0.0520}$	$0.2479_{\pm0.0505}$	$1.0520_{\pm0.2117}$	$0.3097_{\pm0.0243}$
	EDL	$0.5667_{\pm0.0371}$	$0.3269_{\pm0.1655}$	$0.2689_{\pm0.2131}$	$0.2695_{\pm0.1898}$	$0.5160_{\pm0.0309}$	$0.1626_{\pm0.0670}$	$0.7454_{\pm0.0435}$	$0.2669_{\pm0.0160}$
	SWAG	$0.5487_{\pm0.0434}$	$0.4027_{\pm0.0451}$	$0.3213_{\pm0.1143}$	$0.3455_{\pm0.0925}$	$0.5121_{\pm0.0361}$	$0.2072_{\pm0.0546}$	$0.8506_{\pm0.1021}$	$0.2899_{\pm0.0167}$
	MCD	$0.5372_{\pm0.0549}$	$0.3846_{\pm0.0459}$	$0.2426_{\pm0.0941}$	$0.2790_{\pm0.0654}$	$0.4688_{\pm0.0573}$	$0.2808_{\pm0.0582}$	$1.0547_{\pm0.2158}$	$0.3295_{\pm0.0344}$
	DKL	$0.5321_{\pm0.0508}$	$0.3925_{\pm0.0699}$	$0.3541_{\pm0.1110}$	$0.3657_{\pm0.0751}$	$0.4948_{\pm0.0457}$	$0.2987_{\pm0.0481}$	$1.0003_{\pm0.1596}$	$0.3319_{\pm0.0350}$
	SGLD	$0.5654_{\pm0.0103}$	$0.3996_{\pm0.0258}$	$0.2230_{\pm0.0286}$	$0.2855_{\pm0.0277}$	$0.4857_{\pm0.0027}$	$0.2086_{\pm0.0222}$	$0.8494_{\pm0.0096}$	$0.2876_{\pm0.0021}$
-	Ensemble	$0.6051_{\pm0.0305}$	$0.5082_{\pm0.0871}$	$0.2230_{\pm0.1070}$	$0.2938_{\pm0.0896}$	$0.5516_{\pm0.0395}$	$0.1489_{\pm0.0271}$	$0.7549_{\pm0.0523}$	$0.2590_{\pm0.0131}$

Table 8: Results on Immuno-Virus dataset of models trained with Immuno-Tumor dataset.

PLM	Model	Accuracy	Precision	Recall	F1 Score	AUC ROC	ECE	NLL	Brier Score
ESMC	Deterministic	$0.5302_{\pm0.0131}$	$0.7656_{\pm0.0975}$	$0.1176_{\pm0.0377}$	$0.1997_{\pm0.0565}$	$0.4863_{\pm0.0276}$	$0.3658_{\pm0.0476}$	$1.3742_{\pm0.3087}$	$0.3926_{\pm0.0349}$
	TS	$0.5302_{\pm0.0131}$	$0.7656_{\pm0.0975}$	$0.1176_{\pm0.0377}$	$0.1997_{\pm0.0565}$	$0.4863_{\pm0.0276}$	$0.3398_{\pm0.0487}$	$1.1751_{\pm0.2181}$	$0.3730_{\pm0.0352}$
	LA	$0.5214_{\pm0.0138}$	$0.6488_{\pm0.1239}$	$0.2675_{\pm0.2272}$	$0.3149_{\pm0.1471}$	$0.4894_{\pm0.0251}$	$0.3386_{\pm0.0174}$	$1.1512_{\pm0.1057}$	$0.3712_{\pm0.0090}$
	DVBLL	$0.5202_{\pm0.0250}$	$0.6106_{\pm0.1010}$	$0.2457_{\pm0.2552}$	$0.2828_{\pm0.1806}$	$0.5245_{\pm0.0365}$	$0.3053_{\pm0.0488}$	$1.1467_{\pm0.4022}$	$0.3469_{\pm0.0326}$
	EDL	$0.5335_{\pm0.0026}$	$0.8998_{\pm0.0495}$	$0.0918_{\pm0.0075}$	$0.1664_{\pm0.0117}$	$0.5327_{\pm0.0218}$	$0.1901_{\pm0.0146}$	$0.7604_{\pm0.0125}$	$0.2794_{\pm0.0052}$
	SWAG	$0.5151_{\pm0.0216}$	$0.5180_{\pm0.0173}$	$0.6228_{\pm0.0741}$	$0.5640_{\pm0.0381}$	$0.5446_{\pm0.0312}$	$0.2216_{\pm0.0397}$	$0.8729_{\pm0.0726}$	$0.3066_{\pm0.0187}$
	MCD	$0.5297_{\pm0.0114}$	$0.7587_{\pm0.0889}$	$0.1171_{\pm0.0373}$	$0.1989_{\pm0.0561}$	$0.4864_{\pm0.0286}$	$0.3613_{\pm0.0465}$	$1.3082_{\pm0.2571}$	$0.3887_{\pm0.0333}$
	DKL	$0.5025_{\pm0.0050}$	$0.5175_{\pm0.0192}$	$0.4526_{\pm0.1936}$	$0.4536_{\pm0.1311}$	$0.5204_{\pm0.0342}$	$0.2312_{\pm0.0239}$	$0.8579_{\pm0.0733}$	$0.3075_{\pm0.0198}$
	SGLD	$0.5295_{\pm0.0165}$	$0.5355_{\pm0.0154}$	$0.5663_{\pm0.0869}$	$0.5464_{\pm0.0448}$	$0.5556_{\pm0.0118}$	$0.2843_{\pm0.0259}$	$1.0994_{\pm0.0896}$	$0.3382_{\pm0.0132}$
ProstT5	Deterministic	$0.5295_{\pm0.0218}$	$0.6510_{\pm0.0773}$	$0.1727_{\pm0.0483}$	$0.2678_{\pm0.0601}$	$0.5355_{\pm0.0241}$	$0.3278_{\pm0.0836}$	$1.9635_{\pm0.9470}$	$0.3783_{\pm0.0539}$
	TS	$0.5295_{\pm0.0218}$	$0.6510_{\pm0.0773}$	$0.1727_{\pm0.0483}$	$0.2678_{\pm0.0601}$	$0.5355_{\pm0.0241}$	$0.3067_{\pm0.0858}$	$1.6212_{\pm0.7043}$	$0.3648_{\pm0.0546}$
	LA	$0.4965_{\pm0.0277}$	$0.5143_{\pm0.0553}$	$0.1737_{\pm0.1084}$	$0.2396_{\pm0.1332}$	$0.4992_{\pm0.0243}$	$0.3094_{\pm0.0522}$	$1.4220_{\pm0.5568}$	$0.3661_{\pm0.0357}$
	DVBLL	$0.5131_{\pm0.0477}$	$0.5489_{\pm0.0992}$	$0.2432_{\pm0.0728}$	$0.3323_{\pm0.0824}$	$0.5001_{\pm0.0517}$	$0.3589_{\pm0.0691}$	$1.8423_{\pm0.3574}$	$0.4033_{\pm0.0478}$
	EDL	$0.5025_{\pm0.0130}$	$0.4585_{\pm0.2557}$	$0.0640_{\pm0.0452}$	$0.1106_{\pm0.0763}$	$0.5770_{\pm0.0363}$	$0.1322_{\pm0.0315}$	$0.7293_{\pm0.0236}$	$0.2664_{\pm0.0103}$
	SWAG	$0.4879_{\pm0.0351}$	$0.5043_{\pm0.0840}$	$0.2159_{\pm0.0654}$	$0.2951_{\pm0.0623}$	$0.4711_{\pm0.0239}$	$0.2619_{\pm0.0500}$	$1.0543_{\pm0.1345}$	$0.3447_{\pm0.0272}$
	MCD	$0.5292_{\pm0.0219}$	$0.6494_{\pm0.0776}$	$0.1727_{\pm0.0483}$	$0.2678_{\pm0.0602}$	$0.5361_{\pm0.0238}$	$0.3268_{\pm0.0845}$	$1.8868_{\pm0.8771}$	$0.3769_{\pm0.0536}$
	DKL	$0.5118_{\pm0.0231}$	$0.5314_{\pm0.0479}$	$0.2824_{\pm0.1093}$	$0.3568_{\pm0.1075}$	$0.5240_{\pm0.0391}$	$0.1911_{\pm0.1471}$	$0.9252_{\pm0.2077}$	$0.3149_{\pm0.0574}$
	SGLD	$0.4678_{\pm0.0110}$	$0.4050_{\pm0.0363}$	$0.0998_{\pm0.0033}$	$0.1599_{\pm0.0055}$	$0.5206_{\pm0.0191}$	$0.2839_{\pm0.0115}$	$0.9406_{\pm0.0109}$	$0.3295_{\pm0.0030}$
Ankh	Deterministic	$0.5290_{\pm0.0572}$	$0.6970_{\pm0.1879}$	$0.1712_{\pm0.0701}$	$0.2647_{\pm0.0949}$	$0.5391_{\pm0.0878}$	$0.3062_{\pm0.0893}$	$1.0368_{\pm0.1907}$	$0.3417_{\pm0.0465}$
	TS	$0.5290_{\pm0.0572}$	$0.6970_{\pm0.1879}$	$0.1712_{\pm0.0701}$	$0.2647_{\pm0.0949}$	$0.5391_{\pm0.0878}$	$0.2734_{\pm0.0820}$	$0.9315_{\pm0.1319}$	$0.3235_{\pm0.0386}$
	LA	$0.5227_{\pm0.0292}$	$0.5760_{\pm0.0666}$	$0.2437_{\pm0.1616}$	$0.3119_{\pm0.1563}$	$0.5430_{\pm0.0431}$	$0.3623_{\pm0.0515}$	$1.5703_{\pm0.0827}$	$0.3949_{\pm0.0340}$
	DVBLL	$0.5103_{\pm0.0168}$	$0.4120_{\pm0.2084}$	$0.2918_{\pm0.2632}$	$0.3025_{\pm0.2395}$	$0.5297_{\pm0.0341}$	$0.2840_{\pm0.0792}$	$1.2431_{\pm0.5063}$	$0.3448_{\pm0.0535}$
	EDL	$0.5151_{\pm0.0165}$	$0.5790_{\pm0.3120}$	$0.2511_{\pm0.3764}$	$0.2265_{\pm0.2335}$	$0.5782_{\pm0.0594}$	$0.1371_{\pm0.0675}$	$0.7259_{\pm0.0307}$	$0.2651_{\pm0.0142}$
	SWAG	$0.5395_{\pm0.0290}$	$0.6565_{\pm0.0648}$	$0.1906_{\pm0.0719}$	$0.2894_{\pm0.0888}$	$0.6073_{\pm0.0502}$	$0.3376_{\pm0.0455}$	$1.4291_{\pm0.3676}$	$0.3661_{\pm0.0399}$
	MCD	$0.5282_{\pm0.0592}$	$0.6946_{\pm0.1938}$	$0.1707_{\pm0.0708}$	$0.2640_{\pm0.0961}$	$0.5394_{\pm0.0880}$	$0.2997_{\pm0.0882}$	$1.0202_{\pm0.1809}$	$0.3392_{\pm0.0452}$
	DKL	$0.5463_{\pm0.0315}$	$0.7031_{\pm0.0750}$	$0.1836_{\pm0.1008}$	$0.2762_{\pm0.1323}$	$0.5517_{\pm0.0338}$	$0.2883_{\pm0.0641}$	$1.0927_{\pm0.2233}$	$0.3441_{\pm0.0402}$
	SGLD	$0.5554_{\pm0.0032}$	$0.7339_{\pm0.0170}$	$0.1950_{\pm0.0075}$	$0.3080_{\pm0.0088}$	$0.5722_{\pm0.0176}$	$0.2256_{\pm0.0239}$	$0.8436_{\pm0.0329}$	$0.2990_{\pm0.0099}$
ESM2	Deterministic	$0.5486_{\pm0.0217}$	$0.6937_{\pm0.0744}$	$0.2050_{\pm0.0395}$	$0.3137_{\pm0.0486}$	$0.5246_{\pm0.0294}$	$0.3441_{\pm0.0422}$	$1.2609_{\pm0.0627}$	$0.3664_{\pm0.0196}$
	TS	$0.5486_{\pm0.0217}$	$0.6937_{\pm0.0744}$	$0.2050_{\pm0.0395}$	$0.3137_{\pm0.0486}$	$0.5246_{\pm0.0294}$	$0.3347_{\pm0.0299}$	$1.0901_{\pm0.0477}$	$0.3497_{\pm0.0170}$
	LA	$0.5204_{\pm0.0441}$	$0.6619_{\pm0.1800}$	$0.2208_{\pm0.1376}$	$0.2966_{\pm0.1134}$	$0.4866_{\pm0.0192}$	$0.3284_{\pm0.0451}$	$1.2468_{\pm0.3638}$	$0.3694_{\pm0.0360}$
	DVBLL	$0.4899_{\pm0.0447}$	$0.5385_{\pm0.1540}$	$0.3692_{\pm0.1752}$	$0.3999_{\pm0.1208}$	$0.4827_{\pm0.0174}$	$0.3065_{\pm0.0437}$	$1.0635_{\pm0.1095}$	$0.3487_{\pm0.0272}$
	EDL	$0.5471_{\pm0.0196}$	$0.7834_{\pm0.0492}$	$0.1504_{\pm0.0487}$	$0.2485_{\pm0.0705}$	$0.5253_{\pm0.0080}$	$0.1925_{\pm0.0267}$	$0.7558_{\pm0.0107}$	$0.2772_{\pm0.0043}$
	SWAG	$0.5486_{\pm0.0556}$	$0.5832_{\pm0.0902}$	$0.5692_{\pm0.1890}$	$0.5443_{\pm0.1032}$	$0.5968_{\pm0.0547}$	$0.1840_{\pm0.0259}$	$0.7784_{\pm0.0389}$	$0.2742_{\pm0.0131}$
	MCD	$0.5496_{\pm0.0228}$	$0.6964_{\pm0.0750}$	$0.2060_{\pm0.0411}$	$0.3151_{\pm0.0509}$	$0.5249_{\pm0.0289}$	$0.3381_{\pm0.0411}$	$1.1911_{\pm0.0770}$	$0.3614_{\pm0.0206}$
	DKL	$0.5433_{\pm0.0235}$	$0.7057_{\pm0.0982}$	$0.1931_{\pm0.0630}$	$0.2946_{\pm0.0722}$	$0.5365_{\pm0.0597}$	$0.1784_{\pm0.0704}$	$0.7783_{\pm0.0675}$	$0.2819_{\pm0.0250}$
	SGLD	$0.5300_{\pm0.0416}$	$0.6065_{\pm0.1090}$	$0.2754_{\pm0.0430}$	$0.3718_{\pm0.0265}$	$0.5503_{\pm0.0128}$	$0.2211_{\pm0.0308}$	$0.7845_{\pm0.0114}$	$0.2809_{\pm0.0044}$
Prot Bert	Deterministic	$0.5965_{\pm0.0300}$	$0.5977_{\pm0.0230}$	$0.6238_{\pm0.0849}$	$0.6083_{\pm0.0457}$	$0.6969_{\pm0.0289}$	$0.2829_{\pm0.0177}$	$1.0319_{\pm0.1978}$	$0.2922_{\pm0.0214}$
	TS	$0.5965_{\pm0.0300}$	$0.5977_{\pm0.0230}$	$0.6238_{\pm0.0849}$	$0.6083_{\pm0.0457}$	$0.6969_{\pm0.0289}$	$0.2592_{\pm0.0159}$	$0.9066_{\pm0.1444}$	$0.2770_{\pm0.0198}$
	LA	$0.6302_{\pm0.0318}$	$0.6212_{\pm0.0256}$	$0.6943_{\pm0.0837}$	$0.6537_{\pm0.0452}$	$0.7103_{\pm0.0153}$	$0.2324_{\pm0.0372}$	$0.8433_{\pm0.1143}$	$0.2644_{\pm0.0212}$
	DVBLL	$0.6038_{\pm0.0248}$	$0.6058_{\pm0.0252}$	$0.6318_{\pm0.0413}$	$0.6177_{\pm0.0258}$	$0.6484_{\pm0.0544}$	$0.2453_{\pm0.0631}$	$0.9467_{\pm0.2036}$	$0.2889_{\pm0.0387}$
	EDL	$0.6244_{\pm0.0164}$	$0.6561_{\pm0.0285}$	$0.5509_{\pm0.0075}$	$0.5984_{\pm0.0075}$	$0.7319_{\pm0.0037}$	$0.1997_{\pm0.0269}$	$0.6450_{\pm0.0082}$	$0.2263_{\pm0.0039}$
	SWAG	$0.6479_{\pm0.0472}$	$0.6765_{\pm0.1177}$	$0.6566_{\pm0.0821}$	$0.6543_{\pm0.0274}$	$0.6559_{\pm0.0523}$	$0.2282_{\pm0.0424}$	$0.9076_{\pm0.1277}$	$0.2756_{\pm0.0289}$
	MCD	$0.5960_{\pm0.0299}$	$0.5973_{\pm0.0230}$	$0.6233_{\pm0.0852}$	$0.6078_{\pm0.0457}$	$0.6971_{\pm0.0290}$	$0.2802_{\pm0.0166}$	$1.0139_{\pm0.1855}$	$0.2908_{\pm0.0207}$
	DKL	$0.5809_{\pm0.0237}$	$0.5945_{\pm0.0190}$	$0.5464_{\pm0.0860}$	$0.5661_{\pm0.0521}$	$0.6253_{\pm0.0319}$	$0.1035_{\pm0.0696}$	$0.7163_{\pm0.0563}$	$0.2536_{\pm0.0150}$
	SGLD	$0.6118_{\pm0.0077}$	$0.6247_{\pm0.0104}$	$0.5901_{\pm0.0069}$	$0.6068_{\pm0.0051}$	$0.7305_{\pm0.0111}$	$0.2335_{\pm0.0101}$	$0.6865_{\pm0.0185}$	$0.2301_{\pm0.0077}$
-	Ensemble	$0.5332_{\pm0.0240}$	$0.8183_{\pm0.0446}$	$0.1017_{\pm0.0527}$	$0.1769_{\pm0.0814}$	$0.6099_{\pm0.0218}$	$0.2503_{\pm0.0200}$	$0.8390_{\pm0.0491}$	$0.2971_{\pm0.0123}$

Table 9: Results on Immuno-Bacteria dataset of models trained with Immuno-Tumor dataset.

PLM	Model	Accuracy	Precision	Recall	F1 Score	AUC ROC	ECE	NLL	Brier Score
ESMC	Deterministic	$0.4028_{\pm0.0407}$	$0.2686_{\pm0.0464}$	$0.4529_{\pm0.1796}$	$0.3308_{\pm0.0928}$	$0.3723_{\pm0.0252}$	$0.2935_{\pm0.0438}$	$0.9049_{\pm0.0603}$	$0.3263_{\pm0.0296}$
	TS	$0.4028_{\pm0.0407}$	$0.2686_{\pm0.0464}$	$0.4529_{\pm0.1796}$	$0.3308_{\pm0.0928}$	$0.3723_{\pm0.0252}$	$0.2688_{\pm0.0351}$	$0.8502_{\pm0.0467}$	$0.3111_{\pm0.0235}$
	LA	$0.3835_{\pm0.0306}$	$0.2512_{\pm0.0487}$	$0.4207_{\pm0.1894}$	$0.3097_{\pm0.0903}$	$0.3538_{\pm0.0346}$	$0.3742_{\pm0.0426}$	$1.1051_{\pm0.1698}$	$0.3874_{\pm0.0408}$
	DVBLL	$0.3887_{\pm0.0517}$	$0.2938_{\pm0.0303}$	$0.5230_{\pm0.0611}$	$0.3750_{\pm0.0336}$	$0.3830_{\pm0.0546}$	$0.4161_{\pm0.1455}$	$1.6906_{\pm0.7694}$	$0.4460_{\pm0.1199}$
	EDL	$0.5879_{\pm0.0304}$	$0.2753_{\pm0.0260}$	$0.1046_{\pm0.0508}$	$0.1443_{\pm0.0531}$	$0.4137_{\pm0.0290}$	$0.1372_{\pm0.0338}$	$0.6943_{\pm0.0119}$	$0.2495_{\pm0.0059}$
	SWAG	$0.3605_{\pm0.0233}$	$0.2828_{\pm0.0257}$	$0.5563_{\pm0.1456}$	$0.3722_{\pm0.0572}$	$0.3634_{\pm0.0196}$	$0.3829_{\pm0.0820}$	$1.2083_{\pm0.2861}$	$0.4096_{\pm0.0716}$
	MCD	$0.4044_{\pm0.0400}$	$0.2700_{\pm0.0472}$	$0.4563_{\pm0.1821}$	$0.3328_{\pm0.0941}$	$0.3723_{\pm0.0253}$	$0.2920_{\pm0.0423}$	$0.8960_{\pm0.0625}$	$0.3252_{\pm0.0296}$
	DKL	$0.3665_{\pm0.0229}$	$0.3002_{\pm0.0188}$	$0.6103_{\pm0.0824}$	$0.4017_{\pm0.0332}$	$0.4238_{\pm0.0343}$	$0.4142_{\pm0.0516}$	$1.1664_{\pm0.2163}$	$0.4129_{\pm0.0476}$
	SGLD	$0.4927_{\pm0.0388}$	$0.3610_{\pm0.0185}$	$0.5678_{\pm0.0623}$	$0.4391_{\pm0.0176}$	$0.5110_{\pm0.0373}$	$0.3592_{\pm0.0698}$	$1.4161_{\pm0.3192}$	$0.3931_{\pm0.0577}$
ProstT5	Deterministic	$0.4625_{\pm0.0520}$	$0.3355_{\pm0.0275}$	$0.5310_{\pm0.0909}$	$0.4075_{\pm0.0312}$	$0.4732_{\pm0.0296}$	$0.3698_{\pm0.1177}$	$1.6462_{\pm0.8287}$	$0.3949_{\pm0.0947}$
	TS	$0.4625_{\pm0.0520}$	$0.3355_{\pm0.0275}$	$0.5310_{\pm0.0909}$	$0.4075_{\pm0.0312}$	$0.4732_{\pm0.0296}$	$0.3484_{\pm0.1139}$	$1.3929_{\pm0.6126}$	$0.3782_{\pm0.0882}$
	LA	$0.4625_{\pm0.0635}$	$0.3074_{\pm0.0336}$	$0.4368_{\pm0.1547}$	$0.3527_{\pm0.0690}$	$0.4446_{\pm0.0522}$	$0.3276_{\pm0.1483}$	$1.3793_{\pm0.8247}$	$0.3734_{\pm0.1156}$
	DVBLL	$0.4883_{\pm0.0720}$	$0.3673_{\pm0.0548}$	$0.5920_{\pm0.0323}$	$0.4507_{\pm0.0389}$	$0.5049_{\pm0.0557}$	$0.3854_{\pm0.0773}$	$1.6432_{\pm0.4019}$	$0.4114_{\pm0.0697}$
	EDL	$0.4948_{\pm0.0807}$	$0.2675_{\pm0.1343}$	$0.4425_{\pm0.2254}$	$0.3327_{\pm0.1671}$	$0.4621_{\pm0.0310}$	$0.1689_{\pm0.0449}$	$0.7136_{\pm0.0275}$	$0.2597_{\pm0.0133}$
	SWAG	$0.4169_{\pm0.0247}$	$0.3212_{\pm0.0098}$	$0.5977_{\pm0.0850}$	$0.4164_{\pm0.0264}$	$0.4484_{\pm0.0180}$	$0.3289_{\pm0.0347}$	$1.0170_{\pm0.0767}$	$0.3582_{\pm0.0266}$
	MCD	$0.4637_{\pm0.0536}$	$0.3362_{\pm0.0288}$	$0.5299_{\pm0.0909}$	$0.4076_{\pm0.0315}$	$0.4732_{\pm0.0295}$	$0.3681_{\pm0.1174}$	$1.6033_{\pm0.7782}$	$0.3936_{\pm0.0940}$
	DKL	$0.4903_{\pm0.0456}$	$0.3407_{\pm0.0364}$	$0.4793_{\pm0.0634}$	$0.3971_{\pm0.0418}$	$0.4794_{\pm0.0502}$	$0.3080_{\pm0.0468}$	$1.0132_{\pm0.1211}$	$0.3498_{\pm0.0362}$
	SGLD	$0.4181_{\pm0.0198}$	$0.3257_{\pm0.0095}$	$0.6138_{\pm0.0099}$	$0.4255_{\pm0.0083}$	$0.4567_{\pm0.0213}$	$0.3059_{\pm0.0185}$	$0.8994_{\pm0.0294}$	$0.3308_{\pm0.0121}$
Ankh	Deterministic	$0.4246_{\pm0.0659}$	$0.3238_{\pm0.0163}$	$0.6092_{\pm0.2045}$	$0.4107_{\pm0.0781}$	$0.4260_{\pm0.0292}$	$0.3357_{\pm0.0884}$	$1.0949_{\pm0.3797}$	$0.3663_{\pm0.0810}$
	TS	$0.4246_{\pm0.0659}$	$0.3238_{\pm0.0163}$	$0.6092_{\pm0.2045}$	$0.4107_{\pm0.0781}$	$0.4260_{\pm0.0292}$	$0.3157_{\pm0.0842}$	$0.9926_{\pm0.2806}$	$0.3482_{\pm0.0741}$
	LA	$0.4581_{\pm0.0751}$	$0.3111_{\pm0.0173}$	$0.4609_{\pm0.2122}$	$0.3486_{\pm0.0987}$	$0.4513_{\pm0.0469}$	$0.4072_{\pm0.0972}$	$1.6827_{\pm0.4374}$	$0.4303_{\pm0.0823}$
	DVBLL	$0.4327_{\pm0.1095}$	$0.2687_{\pm0.1347}$	$0.6322_{\pm0.3193}$	$0.3769_{\pm0.1891}$	$0.4430_{\pm0.0414}$	$0.3499_{\pm0.1500}$	$1.2469_{\pm0.5561}$	$0.3832_{\pm0.1082}$
	EDL	$0.4992_{\pm0.0952}$	$0.2472_{\pm0.1261}$	$0.3874_{\pm0.3322}$	$0.2851_{\pm0.1675}$	$0.4366_{\pm0.0438}$	$0.1320_{\pm0.0527}$	$0.6891_{\pm0.0182}$	$0.2478_{\pm0.0090}$
	SWAG	$0.4226_{\pm0.0162}$	$0.3330_{\pm0.0058}$	$0.6460_{\pm0.0641}$	$0.4387_{\pm0.0192}$	$0.4679_{\pm0.0163}$	$0.3846_{\pm0.0314}$	$1.3006_{\pm0.1645}$	$0.4024_{\pm0.0291}$
	MCD	$0.4226_{\pm0.0662}$	$0.3233_{\pm0.0140}$	$0.6092_{\pm0.2019}$	$0.4103_{\pm0.0754}$	$0.4270_{\pm0.0288}$	$0.3310_{\pm0.0891}$	$1.0750_{\pm0.3582}$	$0.3635_{\pm0.0797}$
	DKL	$0.4117_{\pm0.0433}$	$0.3128_{\pm0.0129}$	$0.5736_{\pm0.1325}$	$0.4004_{\pm0.0455}$	$0.4445_{\pm0.0202}$	$0.2796_{\pm0.1089}$	$0.9653_{\pm0.3755}$	$0.3277_{\pm0.0799}$
	SGLD	$0.3887_{\pm0.0119}$	$0.3373_{\pm0.0060}$	$0.7701_{\pm0.0315}$	$0.4690_{\pm0.0101}$	$0.4858_{\pm0.0119}$	$0.3372_{\pm0.0210}$	$0.9406_{\pm0.0366}$	$0.3521_{\pm0.0139}$
ESM2	Deterministic	$0.4444_{\pm0.0676}$	$0.3540_{\pm0.0302}$	$0.6701_{\pm0.1004}$	$0.4572_{\pm0.0136}$	$0.5288_{\pm0.0171}$	$0.3452_{\pm0.0611}$	$1.0148_{\pm0.1059}$	$0.3560_{\pm0.0429}$
	TS	$0.4444_{\pm0.0676}$	$0.3540_{\pm0.0302}$	$0.6701_{\pm0.1004}$	$0.4572_{\pm0.0136}$	$0.5288_{\pm0.0171}$	$0.3175_{\pm0.0576}$	$0.9310_{\pm0.0810}$	$0.3356_{\pm0.0354}$
	LA	$0.4520_{\pm0.0449}$	$0.3439_{\pm0.0186}$	$0.6115_{\pm0.1055}$	$0.4367_{\pm0.0275}$	$0.4856_{\pm0.0283}$	$0.3487_{\pm0.0917}$	$1.2261_{\pm0.3594}$	$0.3794_{\pm0.0782}$
	DVBLL	$0.3976_{\pm0.0414}$	$0.3359_{\pm0.0149}$	$0.7299_{\pm0.0690}$	$0.4589_{\pm0.0202}$	$0.4924_{\pm0.0494}$	$0.4056_{\pm0.0769}$	$1.2507_{\pm0.2525}$	$0.4065_{\pm0.0613}$
	EDL	$0.5879_{\pm0.0302}$	$0.4135_{\pm0.0503}$	$0.4299_{\pm0.0862}$	$0.4193_{\pm0.0637}$	$0.5266_{\pm0.0519}$	$0.1496_{\pm0.0097}$	$0.6906_{\pm0.0105}$	$0.2480_{\pm0.0052}$
	SWAG	$0.3996_{\pm0.0259}$	$0.3415_{\pm0.0054}$	$0.7667_{\pm0.0810}$	$0.4714_{\pm0.0171}$	$0.4853_{\pm0.0294}$	$0.3627_{\pm0.0720}$	$1.0931_{\pm0.2055}$	$0.3865_{\pm0.0601}$
	MCD	$0.4419_{\pm0.0660}$	$0.3521_{\pm0.0284}$	$0.6690_{\pm0.1004}$	$0.4556_{\pm0.0152}$	$0.5286_{\pm0.0175}$	$0.3398_{\pm0.0639}$	$0.9928_{\pm0.1017}$	$0.3529_{\pm0.0422}$
	DKL	$0.4411_{\pm0.0807}$	$0.3377_{\pm0.0367}$	$0.5816_{\pm0.1364}$	$0.4180_{\pm0.0371}$	$0.4539_{\pm0.0502}$	$0.2041_{\pm0.0683}$	$0.7578_{\pm0.0777}$	$0.2789_{\pm0.0347}$
	SGLD	$0.6008_{\pm0.0241}$	$0.4454_{\pm0.0276}$	$0.5644_{\pm0.0438}$	$0.4977_{\pm0.0331}$	$0.5887_{\pm0.0330}$	$0.1709_{\pm0.0115}$	$0.7143_{\pm0.0154}$	$0.2522_{\pm0.0072}$
Prot Bert	Deterministic	$0.3915_{\pm0.0215}$	$0.3070_{\pm0.0062}$	$0.5862_{\pm0.0691}$	$0.4020_{\pm0.0207}$	$0.3967_{\pm0.0121}$	$0.4447_{\pm0.0592}$	$1.4657_{\pm0.3094}$	$0.4496_{\pm0.0479}$
	TS	$0.3915_{\pm0.0215}$	$0.3070_{\pm0.0062}$	$0.5862_{\pm0.0691}$	$0.4020_{\pm0.0207}$	$0.3967_{\pm0.0121}$	$0.4070_{\pm0.0567}$	$1.2647_{\pm0.2320}$	$0.4182_{\pm0.0433}$
	LA	$0.3839_{\pm0.0278}$	$0.3141_{\pm0.0105}$	$0.6425_{\pm0.0911}$	$0.4206_{\pm0.0267}$	$0.4331_{\pm0.0319}$	$0.4185_{\pm0.0418}$	$1.2856_{\pm0.2437}$	$0.4243_{\pm0.0450}$
	DVBLL	$0.3819_{\pm0.0220}$	$0.3058_{\pm0.0058}$	$0.6000_{\pm0.0461}$	$0.4046_{\pm0.0121}$	$0.3937_{\pm0.0185}$	$0.4270_{\pm0.1057}$	$1.5204_{\pm0.5202}$	$0.4460_{\pm0.0946}$
	EDL	$0.4367_{\pm0.0098}$	$0.3009_{\pm0.0086}$	$0.4586_{\pm0.0324}$	$0.3632_{\pm0.0153}$	$0.4155_{\pm0.0084}$	$0.2259_{\pm0.0086}$	$0.7645_{\pm0.0146}$	$0.2832_{\pm0.0066}$
	SWAG	$0.4097_{\pm0.0439}$	$0.3236_{\pm0.0125}$	$0.6184_{\pm0.0617}$	$0.4231_{\pm0.0092}$	$0.4183_{\pm0.0406}$	$0.4010_{\pm0.0787}$	$1.3130_{\pm0.4075}$	$0.4196_{\pm0.0707}$
	MCD	$0.3907_{\pm0.0210}$	$0.3063_{\pm0.0064}$	$0.5851_{\pm0.0694}$	$0.4012_{\pm0.0210}$	$0.3958_{\pm0.0127}$	$0.4419_{\pm0.0583}$	$1.4449_{\pm0.2928}$	$0.4474_{\pm0.0466}$
	DKL	$0.3758_{\pm0.0408}$	$0.2979_{\pm0.0096}$	$0.5747_{\pm0.0839}$	$0.3906_{\pm0.0242}$	$0.4173_{\pm0.0110}$	$0.2595_{\pm0.0572}$	$0.8045_{\pm0.0818}$	$0.2993_{\pm0.0338}$
	SGLD	$0.4355_{\pm0.0218}$	$0.3046_{\pm0.0091}$	$0.4736_{\pm0.0301}$	$0.3703_{\pm0.0108}$	$0.4304_{\pm0.0119}$	$0.3051_{\pm0.0160}$	$0.9336_{\pm0.0201}$	$0.3398_{\pm0.0101}$
-	Ensemble	$0.3839_{\pm0.0256}$	$0.3020_{\pm0.0074}$	$0.5782_{\pm0.0681}$	$0.3958_{\pm0.0197}$	$0.4227_{\pm0.0123}$	$0.3122_{\pm0.0403}$	$0.9219_{\pm0.0682}$	$0.3394_{\pm0.0281}$

Table 10: Results on Immuno-Virus dataset of LA, DVBLL and DKL models with varying dimensions for extra linear layer.

PLM	Model	Hidden Layer	Accuracy	Precision	Recall	F1 Score	AUC ROC	ECE	NLL	Brier Score
ESMC	LA	64	$0.9025_{\pm0.0037}$	$0.8863_{\pm0.0080}$	$0.9270_{\pm0.0125}$	$0.9061_{\pm0.0040}$	$0.9641_{\pm0.0028}$	$0.0301_{\pm0.0071}$	$0.2486_{\pm0.0079}$	$0.0725_{\pm0.0025}$
		32	$0.8955_{\pm0.0045}$	$0.8864_{\pm0.0124}$	$0.9112_{\pm0.0127}$	$0.8985_{\pm0.0041}$	$0.9640_{\pm0.0016}$	$0.0327_{\pm0.0071}$	$0.2487_{\pm0.0075}$	$0.0751_{\pm0.0025}$
		128	$0.8942_{\pm0.0076}$	$0.8872_{\pm0.0141}$	$0.9072_{\pm0.0046}$	$0.8970_{\pm0.0065}$	$0.9635_{\pm0.0013}$	$0.0302_{\pm0.0088}$	$0.2504_{\pm0.0071}$	$0.0752_{\pm0.0031}$
	DVBLL	64	$0.8990_{\pm0.0118}$	$0.9004_{\pm0.0117}$	$0.9007_{\pm0.0206}$	$0.9004_{\pm0.0121}$	$0.9613_{\pm0.0068}$	$0.0514_{\pm0.0151}$	$0.2915_{\pm0.0460}$	$0.0775_{\pm0.0088}$
		32	$0.9023_{\pm0.0068}$	$0.8890_{\pm0.0088}$	$0.9231_{\pm0.0237}$	$0.9054_{\pm0.0080}$	$0.9660_{\pm0.0028}$	$0.0380_{\pm0.0056}$	$0.2470_{\pm0.0103}$	$0.0730_{\pm0.0036}$
		128	$0.8990_{\pm0.0065}$	$0.8971_{\pm0.0275}$	$0.9072_{\pm0.0410}$	$0.9009_{\pm0.0095}$	$0.9659_{\pm0.0026}$	$0.0498_{\pm0.0164}$	$0.2552_{\pm0.0144}$	$0.0751_{\pm0.0041}$
	DKL	64	$0.8829_{\pm0.0142}$	$0.8691_{\pm0.0128}$	$0.9057_{\pm0.0193}$	$0.8869_{\pm0.0142}$	$0.9402_{\pm0.0178}$	$0.0398_{\pm0.0116}$	$0.3039_{\pm0.0361}$	$0.0892_{\pm0.0113}$
		32	$0.8917_{\pm0.0082}$	$0.8836_{\pm0.0128}$	$0.9062_{\pm0.0119}$	$0.8947_{\pm0.0078}$	$0.9424_{\pm0.0069}$	$0.1980_{\pm0.0709}$	$0.4309_{\pm0.0723}$	$0.1291_{\pm0.0250}$
		128	$0.9063_{\pm0.0054}$	$0.9015_{\pm0.0211}$	$0.9166_{\pm0.0228}$	$0.9085_{\pm0.0050}$	$0.9487_{\pm0.0085}$	$0.1023_{\pm0.0693}$	$0.3230_{\pm0.0464}$	$0.0889_{\pm0.0145}$
ProstT5	LA	64	$0.8846_{\pm0.0122}$	$0.8855_{\pm0.0061}$	$0.8873_{\pm0.0229}$	$0.8863_{\pm0.0133}$	$0.9540_{\pm0.0108}$	$0.0382_{\pm0.0103}$	$0.2877_{\pm0.0476}$	$0.0845_{\pm0.0105}$
		32	$0.8657_{\pm0.0103}$	$0.8498_{\pm0.0146}$	$0.8938_{\pm0.0069}$	$0.8712_{\pm0.0091}$	$0.9342_{\pm0.0073}$	$0.0591_{\pm0.0106}$	$0.3415_{\pm0.0220}$	$0.1035_{\pm0.0066}$
		128	$0.8690_{\pm0.0127}$	$0.8529_{\pm0.0199}$	$0.8973_{\pm0.0025}$	$0.8744_{\pm0.0106}$	$0.9367_{\pm0.0083}$	$0.0581_{\pm0.0167}$	$0.3349_{\pm0.0291}$	$0.1016_{\pm0.0092}$
	DVBLL	64	$0.8889_{\pm0.0183}$	$0.8872_{\pm0.0073}$	$0.8948_{\pm0.0352}$	$0.8907_{\pm0.0199}$	$0.9520_{\pm0.0103}$	$0.0550_{\pm0.0118}$	$0.3207_{\pm0.0375}$	$0.0876_{\pm0.0105}$
		32	$0.8884_{\pm0.0107}$	$0.9013_{\pm0.0287}$	$0.8789_{\pm0.0495}$	$0.8883_{\pm0.0153}$	$0.9622_{\pm0.0032}$	$0.0512_{\pm0.0241}$	$0.3086_{\pm0.1145}$	$0.0799_{\pm0.0109}$
		128	$0.8937_{\pm0.0165}$	$0.8879_{\pm0.0353}$	$0.9082_{\pm0.0390}$	$0.8966_{\pm0.0157}$	$0.9612_{\pm0.0059}$	$0.0540_{\pm0.0253}$	$0.2865_{\pm0.0497}$	$0.0802_{\pm0.0109}$
	DKL	64	$0.8456_{\pm0.0341}$	$0.8213_{\pm0.0445}$	$0.8928_{\pm0.0218}$	$0.8550_{\pm0.0288}$	$0.8944_{\pm0.0613}$	$0.0820_{\pm0.0492}$	$0.3984_{\pm0.0862}$	$0.1232_{\pm0.0325}$
		32	$0.8894_{\pm0.0071}$	$0.8844_{\pm0.0130}$	$0.9002_{\pm0.0181}$	$0.8920_{\pm0.0074}$	$0.9389_{\pm0.0108}$	$0.2060_{\pm0.1171}$	$0.4522_{\pm0.1383}$	$0.1422_{\pm0.0588}$
		128	$0.8942_{\pm0.0386}$	$0.8850_{\pm0.0506}$	$0.9127_{\pm0.0183}$	$0.8982_{\pm0.0344}$	$0.9354_{\pm0.0237}$	$0.1556_{\pm0.1093}$	$0.4028_{\pm0.1465}$	$0.1230_{\pm0.0575}$
Ankh	LA	64	$0.8804_{\pm0.0180}$	$0.8763_{\pm0.0350}$	$0.8923_{\pm0.0130}$	$0.8837_{\pm0.0144}$	$0.9541_{\pm0.0063}$	$0.0370_{\pm0.0106}$	$0.2790_{\pm0.0217}$	$0.0855_{\pm0.0092}$
		32	$0.8526_{\pm0.0050}$	$0.8518_{\pm0.0120}$	$0.8596_{\pm0.0078}$	$0.8555_{\pm0.0038}$	$0.9462_{\pm0.0011}$	$0.0619_{\pm0.0031}$	$0.3137_{\pm0.0031}$	$0.0965_{\pm0.0011}$
		128	$0.8584_{\pm0.0037}$	$0.8585_{\pm0.0145}$	$0.8640_{\pm0.0120}$	$0.8610_{\pm0.0017}$	$0.9496_{\pm0.0007}$	$0.0614_{\pm0.0041}$	$0.3036_{\pm0.0022}$	$0.0932_{\pm0.0008}$
	DVBLL	64	$0.7426_{\pm0.1883}$	$0.7438_{\pm0.1928}$	$0.9355_{\pm0.0571}$	$0.8079_{\pm0.1102}$	$0.7802_{\pm0.2100}$	$0.0450_{\pm0.0282}$	$0.4686_{\pm0.1857}$	$0.1504_{\pm0.0811}$
		32	$0.8967_{\pm0.0033}$	$0.8940_{\pm0.0183}$	$0.9047_{\pm0.0244}$	$0.8989_{\pm0.0042}$	$0.9628_{\pm0.0013}$	$0.0382_{\pm0.0069}$	$0.2518_{\pm0.0039}$	$0.0748_{\pm0.0007}$
		128	$0.8995_{\pm0.0057}$	$0.8892_{\pm0.0191}$	$0.9171_{\pm0.0151}$	$0.9026_{\pm0.0041}$	$0.9634_{\pm0.0007}$	$0.0339_{\pm0.0087}$	$0.2505_{\pm0.0059}$	$0.0745_{\pm0.0024}$
	DKL	64	$0.8446_{\pm0.0693}$	$0.8380_{\pm0.0918}$	$0.8794_{\pm0.0082}$	$0.8552_{\pm0.0511}$	$0.8882_{\pm0.1303}$	$0.0839_{\pm0.0851}$	$0.3670_{\pm0.1566}$	$0.1174_{\pm0.0608}$
		32	$0.8912_{\pm0.0179}$	$0.8896_{\pm0.0459}$	$0.9022_{\pm0.0322}$	$0.8942_{\pm0.0129}$	$0.9300_{\pm0.0372}$	$0.2119_{\pm0.0614}$	$0.4423_{\pm0.1012}$	$0.1336_{\pm0.0453}$
		128	$0.8831_{\pm0.0556}$	$0.8810_{\pm0.0738}$	$0.8973_{\pm0.0255}$	$0.8879_{\pm0.0477}$	$0.9154_{\pm0.0714}$	$0.0949_{\pm0.0864}$	$0.3545_{\pm0.1652}$	$0.1079_{\pm0.0688}$
ESM2	LA	64	$0.8806_{\pm0.0102}$	$0.8575_{\pm0.0235}$	$0.9186_{\pm0.0191}$	$0.8866_{\pm0.0084}$	$0.9594_{\pm0.0031}$	$0.0578_{\pm0.0175}$	$0.2951_{\pm0.0276}$	$0.0852_{\pm0.0039}$
		32	$0.8587_{\pm0.0042}$	$0.8363_{\pm0.0112}$	$0.8978_{\pm0.0174}$	$0.8657_{\pm0.0047}$	$0.9423_{\pm0.0052}$	$0.0564_{\pm0.0060}$	$0.3213_{\pm0.0160}$	$0.0987_{\pm0.0049}$
		128	$0.8615_{\pm0.0026}$	$0.8373_{\pm0.0088}$	$0.9027_{\pm0.0152}$	$0.8686_{\pm0.0034}$	$0.9455_{\pm0.0025}$	$0.0542_{\pm0.0066}$	$0.3121_{\pm0.0081}$	$0.0957_{\pm0.0030}$
	DVBLL	64	$0.8577_{\pm0.0448}$	$0.8319_{\pm0.0692}$	$0.9146_{\pm0.0281}$	$0.8688_{\pm0.0326}$	$0.9350_{\pm0.0390}$	$0.0656_{\pm0.0412}$	$0.3474_{\pm0.1118}$	$0.1100_{\pm0.0419}$
		32	$0.8935_{\pm0.0106}$	$0.8853_{\pm0.0228}$	$0.9092_{\pm0.0306}$	$0.8964_{\pm0.0110}$	$0.9666_{\pm0.0049}$	$0.0485_{\pm0.0096}$	$0.2462_{\pm0.0197}$	$0.0739_{\pm0.0059}$
		128	$0.8945_{\pm0.0110}$	$0.8830_{\pm0.0127}$	$0.9132_{\pm0.0128}$	$0.8978_{\pm0.0107}$	$0.9658_{\pm0.0035}$	$0.0409_{\pm0.0114}$	$0.2431_{\pm0.0142}$	$0.0730_{\pm0.0046}$
	DKL	64	$0.8950_{\pm0.0171}$	$0.8866_{\pm0.0367}$	$0.9122_{\pm0.0152}$	$0.8985_{\pm0.0135}$	$0.9405_{\pm0.0341}$	$0.0445_{\pm0.0285}$	$0.2937_{\pm0.0698}$	$0.0853_{\pm0.0208}$
		32	$0.8151_{\pm0.1683}$	$0.8084_{\pm0.1621}$	$0.8288_{\pm0.1767}$	$0.8181_{\pm0.1685}$	$0.8521_{\pm0.1833}$	$0.0765_{\pm0.0585}$	$0.3947_{\pm0.1531}$	$0.1211_{\pm0.0651}$
		128	$0.9091_{\pm0.0045}$	$0.8918_{\pm0.0107}$	$0.9345_{\pm0.0109}$	$0.9125_{\pm0.0041}$	$0.9585_{\pm0.0037}$	$0.0903_{\pm0.0744}$	$0.3047_{\pm0.0689}$	$0.0851_{\pm0.0226}$
Prot Bert	LA	64	$0.8887_{\pm0.0086}$	$0.8731_{\pm0.0115}$	$0.9136_{\pm0.0138}$	$0.8928_{\pm0.0084}$	$0.9581_{\pm0.0053}$	$0.0451_{\pm0.0148}$	$0.2909_{\pm0.0233}$	$0.0823_{\pm0.0063}$
		32	$0.8627_{\pm0.0055}$	$0.8971_{\pm0.0083}$	$0.8243_{\pm0.0172}$	$0.8590_{\pm0.0071}$	$0.9421_{\pm0.0021}$	$0.0506_{\pm0.0026}$	$0.3225_{\pm0.0051}$	$0.0978_{\pm0.0018}$
		128	$0.8766_{\pm0.0038}$	$0.8971_{\pm0.0086}$	$0.8551_{\pm0.0137}$	$0.8755_{\pm0.0048}$	$0.9468_{\pm0.0008}$	$0.0497_{\pm0.0055}$	$0.3084_{\pm0.0035}$	$0.0931_{\pm0.0011}$
	DVBLL	64	$0.8932_{\pm0.0115}$	$0.9028_{\pm0.0135}$	$0.8854_{\pm0.0306}$	$0.8936_{\pm0.0130}$	$0.9582_{\pm0.0022}$	$0.0392_{\pm0.0134}$	$0.2873_{\pm0.0203}$	$0.0792_{\pm0.0048}$
		32	$0.8781_{\pm0.0198}$	$0.8418_{\pm0.0443}$	$0.9414_{\pm0.0267}$	$0.8874_{\pm0.0136}$	$0.9651_{\pm0.0037}$	$0.0737_{\pm0.0297}$	$0.2758_{\pm0.0184}$	$0.0845_{\pm0.0078}$
		128	$0.8814_{\pm0.0187}$	$0.8616_{\pm0.0543}$	$0.9216_{\pm0.0417}$	$0.8880_{\pm0.0117}$	$0.9641_{\pm0.0026}$	$0.0681_{\pm0.0335}$	$0.2778_{\pm0.0278}$	$0.0838_{\pm0.0100}$
	DKL	64	$0.8685_{\pm0.0372}$	$0.8535_{\pm0.0409}$	$0.8958_{\pm0.0286}$	$0.8740_{\pm0.0341}$	$0.9171_{\pm0.0610}$	$0.0926_{\pm0.0655}$	$0.3644_{\pm0.1261}$	$0.1096_{\pm0.0408}$
		32	$0.8159_{\pm0.1555}$	$0.8182_{\pm0.1542}$	$0.8268_{\pm0.1398}$	$0.8221_{\pm0.1465}$	$0.8583_{\pm0.1784}$	$0.0592_{\pm0.0445}$	$0.3867_{\pm0.1551}$	$0.1195_{\pm0.0655}$
		128	$0.9111_{\pm0.0077}$	$0.9110_{\pm0.0165}$	$0.9146_{\pm0.0123}$	$0.9126_{\pm0.0071}$	$0.9569_{\pm0.0076}$	$0.0489_{\pm0.0286}$	$0.2679_{\pm0.0242}$	$0.0727_{\pm0.0064}$

Table 11: Results on Immuno-Bacteria dataset of LA, DVBLL and DKL models with varying dimensions for extra linear layer.

PLM	Model	Hidden Layer	Accuracy	Precision	Recall	F1 Score	AUC ROC	ECE	NLL	Brier Score
ESMC	LA	64	$0.8149_{\pm0.0106}$	$0.7722_{\pm0.0253}$	$0.6724_{\pm0.0385}$	$0.7178_{\pm0.0200}$	$0.8655_{\pm0.0069}$	$0.1049_{\pm0.0251}$	$0.5780_{\pm0.1031}$	$0.1487_{\pm0.0073}$
		32	$0.7980_{\pm0.0082}$	$0.7315_{\pm0.0246}$	$0.6759_{\pm0.0662}$	$0.6997_{\pm0.0274}$	$0.8621_{\pm0.0031}$	$0.0520_{\pm0.0086}$	$0.4371_{\pm0.0051}$	$0.1393_{\pm0.0017}$
		128	$0.7968_{\pm0.0098}$	$0.7303_{\pm0.0342}$	$0.6747_{\pm0.0649}$	$0.6983_{\pm0.0255}$	$0.8658_{\pm0.0037}$	$0.0564_{\pm0.0070}$	$0.4296_{\pm0.0048}$	$0.1367_{\pm0.0022}$
	DVBLL	64	$0.7992_{\pm0.0243}$	$0.7194_{\pm0.0539}$	$0.7149_{\pm0.0526}$	$0.7142_{\pm0.0273}$	$0.8486_{\pm0.0178}$	$0.1105_{\pm0.0483}$	$0.5663_{\pm0.1132}$	$0.1587_{\pm0.0148}$
		32	$0.7980_{\pm0.0176}$	$0.7124_{\pm0.0585}$	$0.7402_{\pm0.0854}$	$0.7191_{\pm0.0147}$	$0.8738_{\pm0.0095}$	$0.0890_{\pm0.0372}$	$0.4842_{\pm0.0550}$	$0.1444_{\pm0.0103}$
		128	$0.8185_{\pm0.0088}$	$0.7640_{\pm0.0388}$	$0.7057_{\pm0.0516}$	$0.7312_{\pm0.0171}$	$0.8747_{\pm0.0065}$	$0.0784_{\pm0.0222}$	$0.4744_{\pm0.0537}$	$0.1370_{\pm0.0053}$
	DKL	64	$0.8048_{\pm0.0053}$	$0.7323_{\pm0.0283}$	$0.7046_{\pm0.0433}$	$0.7165_{\pm0.0126}$	$0.8399_{\pm0.0100}$	$0.0862_{\pm0.0187}$	$0.5011_{\pm0.0296}$	$0.1546_{\pm0.0056}$
		32	$0.8238_{\pm0.0078}$	$0.7732_{\pm0.0329}$	$0.7103_{\pm0.0512}$	$0.7382_{\pm0.0154}$	$0.8474_{\pm0.0045}$	$0.2381_{\pm0.0558}$	$0.5909_{\pm0.0652}$	$0.2005_{\pm0.0303}$
		128	$0.8169_{\pm0.0104}$	$0.7529_{\pm0.0311}$	$0.7172_{\pm0.0528}$	$0.7325_{\pm0.0216}$	$0.8488_{\pm0.0142}$	$0.1542_{\pm0.0862}$	$0.5258_{\pm0.0768}$	$0.1722_{\pm0.0322}$
ProstT5	LA	64	$0.7742_{\pm0.0244}$	$0.6861_{\pm0.0435}$	$0.6690_{\pm0.0904}$	$0.6727_{\pm0.0471}$	$0.8292_{\pm0.0219}$	$0.0954_{\pm0.0312}$	$0.5275_{\pm0.0489}$	$0.1656_{\pm0.0141}$
		32	$0.7863_{\pm0.0029}$	$0.7212_{\pm0.0107}$	$0.6379_{\pm0.0221}$	$0.6767_{\pm0.0090}$	$0.8421_{\pm0.0018}$	$0.0437_{\pm0.0050}$	$0.4669_{\pm0.0027}$	$0.1516_{\pm0.0009}$
		128	$0.7855_{\pm0.0027}$	$0.7193_{\pm0.0079}$	$0.6379_{\pm0.0241}$	$0.6758_{\pm0.0108}$	$0.8445_{\pm0.0022}$	$0.0350_{\pm0.0059}$	$0.4644_{\pm0.0041}$	$0.1508_{\pm0.0012}$
	DVBLL	64	$0.7859_{\pm0.0109}$	$0.7202_{\pm0.0419}$	$0.6552_{\pm0.1070}$	$0.6774_{\pm0.0510}$	$0.8288_{\pm0.0312}$	$0.1033_{\pm0.0553}$	$0.6550_{\pm0.1951}$	$0.1685_{\pm0.0158}$
		32	$0.7750_{\pm0.0636}$	$0.6019_{\pm0.3018}$	$0.5368_{\pm0.2702}$	$0.5667_{\pm0.2838}$	$0.8437_{\pm0.0185}$	$0.1091_{\pm0.0705}$	$0.5534_{\pm0.1100}$	$0.1683_{\pm0.0429}$
		128	$0.7677_{\pm0.0373}$	$0.7349_{\pm0.1075}$	$0.6230_{\pm0.2200}$	$0.6287_{\pm0.1392}$	$0.8521_{\pm0.0055}$	$0.1063_{\pm0.0699}$	$0.5013_{\pm0.0593}$	$0.1645_{\pm0.0235}$
	DKL	64	$0.7750_{\pm0.0112}$	$0.7147_{\pm0.0183}$	$0.5989_{\pm0.0556}$	$0.6499_{\pm0.0313}$	$0.7993_{\pm0.0430}$	$0.0553_{\pm0.0102}$	$0.5074_{\pm0.0235}$	$0.1653_{\pm0.0093}$
		32	$0.8048_{\pm0.0101}$	$0.7659_{\pm0.0149}$	$0.6391_{\pm0.0266}$	$0.6965_{\pm0.0190}$	$0.8009_{\pm0.0279}$	$0.2074_{\pm0.0450}$	$0.5866_{\pm0.0519}$	$0.1982_{\pm0.0243}$
		128	$0.7968_{\pm0.0170}$	$0.7285_{\pm0.0226}$	$0.6701_{\pm0.0412}$	$0.6977_{\pm0.0305}$	$0.8117_{\pm0.0442}$	$0.1189_{\pm0.0676}$	$0.5241_{\pm0.0711}$	$0.1702_{\pm0.0325}$
Ankh	LA	64	$0.7819_{\pm0.0105}$	$0.7182_{\pm0.0409}$	$0.6379_{\pm0.0898}$	$0.6690_{\pm0.0412}$	$0.8504_{\pm0.0018}$	$0.0976_{\pm0.0337}$	$0.5599_{\pm0.1258}$	$0.1565_{\pm0.0063}$
		32	$0.7927_{\pm0.0071}$	$0.7427_{\pm0.0279}$	$0.6299_{\pm0.0314}$	$0.6805_{\pm0.0109}$	$0.8458_{\pm0.0050}$	$0.0545_{\pm0.0078}$	$0.4628_{\pm0.0129}$	$0.1488_{\pm0.0043}$
		128	$0.7927_{\pm0.0027}$	$0.7329_{\pm0.0217}$	$0.6471_{\pm0.0334}$	$0.6862_{\pm0.0096}$	$0.8502_{\pm0.0019}$	$0.0411_{\pm0.0108}$	$0.4514_{\pm0.0051}$	$0.1448_{\pm0.0021}$
	DVBLL	64	$0.7073_{\pm0.0688}$	$0.4427_{\pm0.3630}$	$0.2816_{\pm0.3359}$	$0.2887_{\pm0.3358}$	$0.6239_{\pm0.1697}$	$0.0694_{\pm0.0334}$	$0.6513_{\pm0.1136}$	$0.2024_{\pm0.0378}$
		32	$0.8040_{\pm0.0103}$	$0.7537_{\pm0.0547}$	$0.6747_{\pm0.0895}$	$0.7047_{\pm0.0316}$	$0.8558_{\pm0.0029}$	$0.0806_{\pm0.0155}$	$0.4719_{\pm0.0172}$	$0.1444_{\pm0.0038}$
		128	$0.8028_{\pm0.0080}$	$0.7460_{\pm0.0524}$	$0.6816_{\pm0.0853}$	$0.7058_{\pm0.0279}$	$0.8566_{\pm0.0014}$	$0.0732_{\pm0.0125}$	$0.4641_{\pm0.0118}$	$0.1439_{\pm0.0042}$
	DKL	64	$0.7899_{\pm0.0260}$	$0.7051_{\pm0.0485}$	$0.6966_{\pm0.0194}$	$0.7001_{\pm0.0291}$	$0.8351_{\pm0.0182}$	$0.1098_{\pm0.0136}$	$0.5370_{\pm0.0317}$	$0.1592_{\pm0.0117}$
		32	$0.8173_{\pm0.0114}$	$0.7661_{\pm0.0283}$	$0.6920_{\pm0.0187}$	$0.7267_{\pm0.0147}$	$0.8343_{\pm0.0105}$	$0.1869_{\pm0.0838}$	$0.5511_{\pm0.0752}$	$0.1831_{\pm0.0329}$
		128	$0.8032_{\pm0.0171}$	$0.7485_{\pm0.0285}$	$0.6678_{\pm0.0950}$	$0.7007_{\pm0.0477}$	$0.8302_{\pm0.0192}$	$0.1581_{\pm0.0807}$	$0.5368_{\pm0.0788}$	$0.1773_{\pm0.0340}$
ESM2	LA	64	$0.8028_{\pm0.0190}$	$0.7285_{\pm0.0473}$	$0.7103_{\pm0.0663}$	$0.7157_{\pm0.0283}$	$0.8630_{\pm0.0109}$	$0.0994_{\pm0.0382}$	$0.5091_{\pm0.0832}$	$0.1499_{\pm0.0169}$
		32	$0.7935_{\pm0.0101}$	$0.7152_{\pm0.0063}$	$0.6839_{\pm0.0453}$	$0.6984_{\pm0.0241}$	$0.8546_{\pm0.0029}$	$0.0498_{\pm0.0095}$	$0.4497_{\pm0.0041}$	$0.1447_{\pm0.0016}$
		128	$0.7956_{\pm0.0107}$	$0.7197_{\pm0.0107}$	$0.6839_{\pm0.0475}$	$0.7004_{\pm0.0248}$	$0.8555_{\pm0.0030}$	$0.0518_{\pm0.0096}$	$0.4491_{\pm0.0053}$	$0.1443_{\pm0.0021}$
	DVBLL	64	$0.8129_{\pm0.0083}$	$0.7522_{\pm0.0335}$	$0.7023_{\pm0.0450}$	$0.7244_{\pm0.0135}$	$0.8492_{\pm0.0260}$	$0.0963_{\pm0.0219}$	$0.5986_{\pm0.0949}$	$0.1480_{\pm0.0082}$
		32	$0.8121_{\pm0.0112}$	$0.7503_{\pm0.0431}$	$0.7103_{\pm0.0913}$	$0.7235_{\pm0.0349}$	$0.8697_{\pm0.0043}$	$0.0751_{\pm0.0207}$	$0.4586_{\pm0.0163}$	$0.1389_{\pm0.0052}$
		128	$0.8125_{\pm0.0034}$	$0.7537_{\pm0.0186}$	$0.6943_{\pm0.0420}$	$0.7215_{\pm0.0152}$	$0.8688_{\pm0.0014}$	$0.0483_{\pm0.0137}$	$0.4423_{\pm0.0097}$	$0.1354_{\pm0.0016}$
	DKL	64	$0.8093_{\pm0.0103}$	$0.7631_{\pm0.0135}$	$0.6621_{\pm0.0347}$	$0.7085_{\pm0.0208}$	$0.8159_{\pm0.0450}$	$0.1139_{\pm0.0505}$	$0.5357_{\pm0.0542}$	$0.1601_{\pm0.0204}$
		32	$0.8077_{\pm0.0079}$	$0.7333_{\pm0.0105}$	$0.7103_{\pm0.0316}$	$0.7212_{\pm0.0162}$	$0.8203_{\pm0.0178}$	$0.2735_{\pm0.0196}$	$0.6498_{\pm0.0232}$	$0.2284_{\pm0.0115}$
		128	$0.8190_{\pm0.0106}$	$0.7411_{\pm0.0192}$	$0.7448_{\pm0.0232}$	$0.7426_{\pm0.0151}$	$0.8495_{\pm0.0217}$	$0.0781_{\pm0.0364}$	$0.4599_{\pm0.0173}$	$0.1434_{\pm0.0068}$
Prot Bert	LA	64	$0.8028_{\pm0.0115}$	$0.7405_{\pm0.0490}$	$0.6897_{\pm0.0723}$	$0.7093_{\pm0.0209}$	$0.8531_{\pm0.0095}$	$0.1002_{\pm0.0119}$	$0.5550_{\pm0.0812}$	$0.1509_{\pm0.0080}$
		32	$0.8065_{\pm0.0013}$	$0.7450_{\pm0.0174}$	$0.6839_{\pm0.0345}$	$0.7122_{\pm0.0118}$	$0.8568_{\pm0.0026}$	$0.0395_{\pm0.0039}$	$0.4449_{\pm0.0038}$	$0.1417_{\pm0.0014}$
		128	$0.8069_{\pm0.0045}$	$0.7456_{\pm0.0201}$	$0.6851_{\pm0.0387}$	$0.7129_{\pm0.0144}$	$0.8601_{\pm0.0014}$	$0.0413_{\pm0.0132}$	$0.4397_{\pm0.0025}$	$0.1398_{\pm0.0010}$
	DVBLL	64	$0.8117_{\pm0.0091}$	$0.7481_{\pm0.0255}$	$0.7011_{\pm0.0262}$	$0.7231_{\pm0.0114}$	$0.8617_{\pm0.0114}$	$0.0764_{\pm0.0159}$	$0.5104_{\pm0.0439}$	$0.1424_{\pm0.0034}$
		32	$0.7931_{\pm0.0207}$	$0.7087_{\pm0.0690}$	$0.7402_{\pm0.1345}$	$0.7103_{\pm0.0466}$	$0.8671_{\pm0.0082}$	$0.0997_{\pm0.0464}$	$0.4646_{\pm0.0331}$	$0.1496_{\pm0.0131}$
		128	$0.8056_{\pm0.0141}$	$0.7352_{\pm0.0714}$	$0.7310_{\pm0.0967}$	$0.7235_{\pm0.0187}$	$0.8701_{\pm0.0088}$	$0.0907_{\pm0.0428}$	$0.4563_{\pm0.0243}$	$0.1450_{\pm0.0104}$
	DKL	64	$0.8048_{\pm0.0125}$	$0.7350_{\pm0.0347}$	$0.7011_{\pm0.0473}$	$0.7156_{\pm0.0179}$	$0.8436_{\pm0.0139}$	$0.0723_{\pm0.0225}$	$0.4797_{\pm0.0234}$	$0.1488_{\pm0.0065}$
		32	$0.8331_{\pm0.0067}$	$0.7681_{\pm0.0148}$	$0.7517_{\pm0.0250}$	$0.7594_{\pm0.0116}$	$0.8553_{\pm0.0085}$	$0.1941_{\pm0.0709}$	$0.5363_{\pm0.0740}$	$0.1757_{\pm0.0328}$
		128	$0.8351_{\pm0.0087}$	$0.7687_{\pm0.0236}$	$0.7598_{\pm0.0123}$	$0.7638_{\pm0.0087}$	$0.8476_{\pm0.0249}$	$0.0860_{\pm0.0430}$	$0.4479_{\pm0.0226}$	$0.1379_{\pm0.0082}$

Table 12: Results on Immuno-Tumor dataset of LA, DVBLL and DKL models with varying dimensions for extra linear layer.

PLM	Model	Hidden Layer	Accuracy	Precision	Recall	F1 Score	AUC ROC	ECE	NLL	Brier Score
ESMC	LA	64	$0.7474_{\pm 0.0205}$	$0.6722_{\pm 0.0378}$	$0.7115_{\pm 0.1397}$	$0.6814_{\pm 0.0567}$	$0.8465_{\pm 0.0227}$	$0.0979_{\pm 0.0213}$	$0.4886_{\pm 0.0374}$	$0.1632_{\pm 0.0128}$
		32	$0.7474_{\pm 0.0096}$	$0.6733_{\pm 0.0219}$	$0.6918_{\pm 0.0334}$	$0.6815_{\pm 0.0122}$	$0.8259_{\pm 0.0079}$	$0.0858_{\pm 0.0106}$	$0.5161_{\pm 0.0089}$	$0.1733_{\pm 0.0032}$
		128	$0.7397_{\pm 0.0119}$	$0.6594_{\pm 0.0315}$	$0.7049_{\pm 0.0622}$	$0.6784_{\pm 0.0181}$	$0.8275_{\pm 0.0066}$	$0.0886_{\pm 0.0101}$	$0.5119_{\pm 0.0119}$	$0.1726_{\pm 0.0042}$
	DVBLL	64	$0.7615_{\pm 0.0124}$	$0.6716_{\pm 0.0276}$	$0.7803_{\pm 0.1229}$	$0.7144_{\pm 0.0463}$	$0.8457_{\pm 0.0084}$	$0.1333_{\pm 0.0406}$	$0.5827_{\pm 0.1026}$	$0.1753_{\pm 0.0133}$
		32	$0.7359_{\pm 0.0591}$	$0.6487_{\pm 0.0815}$	$0.7803_{\pm 0.0700}$	$0.7012_{\pm 0.0320}$	$0.8418_{\pm 0.0083}$	$0.1326_{\pm 0.0759}$	$0.5470_{\pm 0.1329}$	$0.1802_{\pm 0.0404}$
		128	$0.7423_{\pm 0.0227}$	$0.6674_{\pm 0.0535}$	$0.7082_{\pm 0.1023}$	$0.6794_{\pm 0.0411}$	$0.8374_{\pm 0.0194}$	$0.0915_{\pm 0.0131}$	$0.5078_{\pm 0.0289}$	$0.1673_{\pm 0.0088}$
	DKL	64	$0.7487_{\pm 0.0416}$	$0.6602_{\pm 0.0606}$	$0.7574_{\pm 0.0953}$	$0.7008_{\pm 0.0504}$	$0.8061_{\pm 0.0224}$	$0.1135_{\pm 0.0240}$	$0.5526_{\pm 0.0384}$	$0.1800_{\pm 0.0140}$
		32	$0.5872_{\pm 0.1062}$	$0.5312_{\pm 0.1512}$	$0.4885_{\pm 0.1673}$	$0.4703_{\pm 0.1092}$	$0.5841_{\pm 0.1127}$	$0.1409_{\pm 0.0423}$	$0.6890_{\pm 0.0068}$	$0.2479_{\pm 0.0034}$
		128	$0.6603_{\pm 0.0966}$	$0.5518_{\pm 0.1055}$	$0.7049_{\pm 0.1579}$	$0.6155_{\pm 0.1210}$	$0.6927_{\pm 0.1209}$	$0.1781_{\pm 0.0572}$	$0.6843_{\pm 0.0065}$	$0.2456_{\pm 0.0032}$
ProstT5	LA	64	$0.7000_{\pm 0.0285}$	$0.6559_{\pm 0.0814}$	$0.5279_{\pm 0.1292}$	$0.5716_{\pm 0.0701}$	$0.7619_{\pm 0.0102}$	$0.1689_{\pm 0.0602}$	$0.7930_{\pm 0.2498}$	$0.2221_{\pm 0.0245}$
		32	$0.6821_{\pm 0.0065}$	$0.6332_{\pm 0.0154}$	$0.4459_{\pm 0.0262}$	$0.5227_{\pm 0.0168}$	$0.7303_{\pm 0.0035}$	$0.0651_{\pm 0.0129}$	$0.5959_{\pm 0.0012}$	$0.2057_{\pm 0.0006}$
		128	$0.6782_{\pm 0.0063}$	$0.6202_{\pm 0.0179}$	$0.4623_{\pm 0.0523}$	$0.5275_{\pm 0.0299}$	$0.7365_{\pm 0.0101}$	$0.0538_{\pm 0.0183}$	$0.5904_{\pm 0.0071}$	$0.2035_{\pm 0.0030}$
	DVBLL	64	$0.6949_{\pm 0.0155}$	$0.6043_{\pm 0.0217}$	$0.6426_{\pm 0.0562}$	$0.6213_{\pm 0.0268}$	$0.7664_{\pm 0.0257}$	$0.2191_{\pm 0.0299}$	$0.9336_{\pm 0.1359}$	$0.2441_{\pm 0.0222}$
		32	$0.6821_{\pm 0.0386}$	$0.5046_{\pm 0.2524}$	$0.4525_{\pm 0.2411}$	$0.4738_{\pm 0.2410}$	$0.6959_{\pm 0.1193}$	$0.0745_{\pm 0.0181}$	$0.5962_{\pm 0.0474}$	$0.2062_{\pm 0.0208}$
		128	$0.6756_{\pm 0.0336}$	$0.4838_{\pm 0.2431}$	$0.5213_{\pm 0.2924}$	$0.4930_{\pm 0.2509}$	$0.7118_{\pm 0.0891}$	$0.0934_{\pm 0.0479}$	$0.6098_{\pm 0.0454}$	$0.2109_{\pm 0.0197}$
	DKL	64	$0.6949_{\pm 0.0224}$	$0.6274_{\pm 0.0233}$	$0.5377_{\pm 0.0964}$	$0.5751_{\pm 0.0602}$	$0.7251_{\pm 0.0402}$	$0.1611_{\pm 0.0487}$	$0.6982_{\pm 0.1190}$	$0.2232_{\pm 0.0270}$
		32	$0.5397_{\pm 0.0771}$	$0.4429_{\pm 0.1109}$	$0.3934_{\pm 0.0796}$	$0.3997_{\pm 0.0299}$	$0.5379_{\pm 0.0886}$	$0.1175_{\pm 0.0191}$	$0.6920_{\pm 0.0018}$	$0.2494_{\pm 0.0009}$
		128	$0.7064_{\pm 0.0439}$	$0.6160_{\pm 0.0291}$	$0.6295_{\pm 0.2528}$	$0.5895_{\pm 0.1887}$	$0.7511_{\pm 0.0663}$	$0.1702_{\pm 0.0493}$	$0.6522_{\pm 0.0406}$	$0.2306_{\pm 0.0184}$
Ankh	LA	64	$0.7462_{\pm 0.0333}$	$0.7076_{\pm 0.0309}$	$0.6131_{\pm 0.1810}$	$0.6381_{\pm 0.1081}$	$0.8276_{\pm 0.0144}$	$0.1805_{\pm 0.0228}$	$0.7586_{\pm 0.0333}$	$0.2006_{\pm 0.0173}$
		32	$0.6474_{\pm 0.0301}$	$0.6722_{\pm 0.3717}$	$0.2295_{\pm 0.2909}$	$0.2508_{\pm 0.2402}$	$0.7244_{\pm 0.0598}$	$0.0900_{\pm 0.0319}$	$0.6205_{\pm 0.0213}$	$0.2159_{\pm 0.0097}$
		128	$0.6551_{\pm 0.0296}$	$0.5947_{\pm 0.3154}$	$0.2885_{\pm 0.3322}$	$0.3055_{\pm 0.2340}$	$0.7491_{\pm 0.0577}$	$0.0976_{\pm 0.0511}$	$0.6117_{\pm 0.0250}$	$0.2115_{\pm 0.0121}$
	DVBLL	64	$0.7282_{\pm 0.0605}$	$0.5144_{\pm 0.2576}$	$0.6885_{\pm 0.3464}$	$0.5882_{\pm 0.2944}$	$0.7661_{\pm 0.1373}$	$0.1314_{\pm 0.0496}$	$0.6086_{\pm 0.1190}$	$0.1925_{\pm 0.0410}$
		32	$0.7179_{\pm 0.0272}$	$0.7196_{\pm 0.0693}$	$0.5115_{\pm 0.1971}$	$0.5676_{\pm 0.1021}$	$0.8198_{\pm 0.0127}$	$0.1012_{\pm 0.0043}$	$0.5253_{\pm 0.0219}$	$0.1794_{\pm 0.0078}$
		128	$0.7038_{\pm 0.0124}$	$0.6740_{\pm 0.0532}$	$0.5180_{\pm 0.1718}$	$0.5651_{\pm 0.0689}$	$0.7939_{\pm 0.0110}$	$0.0935_{\pm 0.0134}$	$0.5527_{\pm 0.0222}$	$0.1892_{\pm 0.0080}$
	DKL	64	$0.7167_{\pm 0.0328}$	$0.6349_{\pm 0.0513}$	$0.6787_{\pm 0.0965}$	$0.6497_{\pm 0.0431}$	$0.7703_{\pm 0.0280}$	$0.1194_{\pm 0.0755}$	$0.6275_{\pm 0.0775}$	$0.2052_{\pm 0.0151}$
		32	$0.5551_{\pm 0.0686}$	$0.4298_{\pm 0.0886}$	$0.5213_{\pm 0.1739}$	$0.4665_{\pm 0.1271}$	$0.5530_{\pm 0.0941}$	$0.1069_{\pm 0.0030}$	$0.6796_{\pm 0.0262}$	$0.2434_{\pm 0.0128}$
		128	$0.5372_{\pm 0.0426}$	$0.3969_{\pm 0.0611}$	$0.3475_{\pm 0.0457}$	$0.3704_{\pm 0.0522}$	$0.5191_{\pm 0.0673}$	$0.1087_{\pm 0.0017}$	$0.6923_{\pm 0.0008}$	$0.2496_{\pm 0.0004}$
ESM2	LA	64	$0.7513_{\pm 0.0174}$	$0.6871_{\pm 0.0399}$	$0.6852_{\pm 0.0751}$	$0.6819_{\pm 0.0229}$	$0.8255_{\pm 0.0074}$	$0.1233_{\pm 0.0430}$	$0.6226_{\pm 0.1342}$	$0.1841_{\pm 0.0169}$
		32	$0.6372_{\pm 0.0104}$	$0.5547_{\pm 0.0237}$	$0.3738_{\pm 0.0191}$	$0.4460_{\pm 0.0131}$	$0.7007_{\pm 0.0069}$	$0.0523_{\pm 0.0058}$	$0.5991_{\pm 0.0046}$	$0.2084_{\pm 0.0019}$
		128	$0.6333_{\pm 0.0085}$	$0.5474_{\pm 0.0184}$	$0.3639_{\pm 0.0191}$	$0.4368_{\pm 0.0148}$	$0.6972_{\pm 0.0122}$	$0.0594_{\pm 0.0037}$	$0.6018_{\pm 0.0081}$	$0.2096_{\pm 0.0033}$
	DVBLL	64	$0.7295_{\pm 0.0296}$	$0.6392_{\pm 0.0349}$	$0.7115_{\pm 0.0998}$	$0.6700_{\pm 0.0511}$	$0.8046_{\pm 0.0191}$	$0.1297_{\pm 0.0470}$	$0.6288_{\pm 0.1167}$	$0.1859_{\pm 0.0220}$
		32	$0.6936_{\pm 0.0169}$	$0.6631_{\pm 0.1342}$	$0.5934_{\pm 0.1971}$	$0.5797_{\pm 0.1191}$	$0.7733_{\pm 0.0161}$	$0.0867_{\pm 0.0210}$	$0.5553_{\pm 0.0200}$	$0.1912_{\pm 0.0081}$
		128	$0.6987_{\pm 0.0081}$	$0.6429_{\pm 0.0644}$	$0.5738_{\pm 0.1364}$	$0.5887_{\pm 0.0691}$	$0.7839_{\pm 0.0075}$	$0.0600_{\pm 0.0182}$	$0.5389_{\pm 0.0092}$	$0.1847_{\pm 0.0033}$
	DKL	64	$0.7551_{\pm 0.0143}$	$0.7010_{\pm 0.0646}$	$0.6984_{\pm 0.1671}$	$0.6792_{\pm 0.0769}$	$0.7901_{\pm 0.0301}$	$0.1516_{\pm 0.0636}$	$0.5972_{\pm 0.0541}$	$0.2043_{\pm 0.0241}$
		32	$0.6513_{\pm 0.0746}$	$0.5562_{\pm 0.1021}$	$0.5574_{\pm 0.1340}$	$0.5520_{\pm 0.1043}$	$0.6702_{\pm 0.1189}$	$0.1608_{\pm 0.0547}$	$0.6867_{\pm 0.0048}$	$0.2468_{\pm 0.0024}$
		128	$0.7013_{\pm 0.1146}$	$0.6462_{\pm 0.1510}$	$0.5934_{\pm 0.1349}$	$0.6109_{\pm 0.1250}$	$0.7190_{\pm 0.1239}$	$0.2031_{\pm 0.0561}$	$0.6836_{\pm 0.0131}$	$0.2356_{\pm 0.0167}$
Prot Bert	LA	64	$0.7359_{\pm 0.0248}$	$0.6444_{\pm 0.0400}$	$0.7410_{\pm 0.0512}$	$0.6871_{\pm 0.0203}$	$0.7964_{\pm 0.0264}$	$0.1131_{\pm 0.0375}$	$0.5894_{\pm 0.0641}$	$0.1892_{\pm 0.0146}$
		32	$0.6231_{\pm 0.0124}$	$0.5170_{\pm 0.0145}$	$0.5344_{\pm 0.0396}$	$0.5252_{\pm 0.0254}$	$0.6873_{\pm 0.0061}$	$0.0498_{\pm 0.0037}$	$0.6132_{\pm 0.0015}$	$0.2145_{\pm 0.0007}$
		128	$0.6372_{\pm 0.0096}$	$0.5316_{\pm 0.0113}$	$0.6098_{\pm 0.0123}$	$0.5680_{\pm 0.0092}$	$0.6970_{\pm 0.0068}$	$0.0603_{\pm 0.0070}$	$0.6107_{\pm 0.0041}$	$0.2134_{\pm 0.0019}$
	DVBLL	64	$0.7333_{\pm 0.0271}$	$0.6381_{\pm 0.0437}$	$0.7508_{\pm 0.0318}$	$0.6883_{\pm 0.0204}$	$0.7947_{\pm 0.0288}$	$0.1420_{\pm 0.0277}$	$0.6521_{\pm 0.1097}$	$0.2014_{\pm 0.0226}$
		32	$0.6603_{\pm 0.0332}$	$0.5514_{\pm 0.0350}$	$0.7803_{\pm 0.0948}$	$0.6413_{\pm 0.0234}$	$0.7454_{\pm 0.0301}$	$0.1259_{\pm 0.0674}$	$0.6235_{\pm 0.0487}$	$0.2169_{\pm 0.0203}$
		128	$0.6385_{\pm 0.0302}$	$0.4974_{\pm 0.0854}$	$0.6197_{\pm 0.3002}$	$0.5160_{\pm 0.2288}$	$0.7254_{\pm 0.0317}$	$0.1225_{\pm 0.0685}$	$0.6350_{\pm 0.0434}$	$0.2224_{\pm 0.0164}$
	DKL	64	$0.7731_{\pm 0.0283}$	$0.7086_{\pm 0.0567}$	$0.7311_{\pm 0.0610}$	$0.7159_{\pm 0.0277}$	$0.7983_{\pm 0.0298}$	$0.1341_{\pm 0.0840}$	$0.5727_{\pm 0.0530}$	$0.1931_{\pm 0.0237}$
		32	$0.5603_{\pm 0.0462}$	$0.4323_{\pm 0.0682}$	$0.4820_{\pm 0.1497}$	$0.4518_{\pm 0.1060}$	$0.5686_{\pm 0.0772}$	$0.1111_{\pm 0.0061}$	$0.6917_{\pm 0.0012}$	$0.2493_{\pm 0.0006}$
		128	$0.6628_{\pm 0.0853}$	$0.5826_{\pm 0.1219}$	$0.4852_{\pm 0.1658}$	$0.5174_{\pm 0.1499}$	$0.6604_{\pm 0.1356}$	$0.1750_{\pm 0.0618}$	$0.6883_{\pm 0.0045}$	$0.2476_{\pm 0.0022}$
